# Time-resolved relaxation and fragmentation of polycyclic aromatic hydrocarbons investigated in the ultrafast XUV-IR regime

**DOI:** 10.1038/s41467-021-26193-z

**Published:** 2021-10-20

**Authors:** J. W. L. Lee, D. S. Tikhonov, P. Chopra, S. Maclot, A. L. Steber, S. Gruet, F. Allum, R. Boll, X. Cheng, S. Düsterer, B. Erk, D. Garg, L. He, D. Heathcote, M. Johny, M. M. Kazemi, H. Köckert, J. Lahl, A. K. Lemmens, D. Loru, R. Mason, E. Müller, T. Mullins, P. Olshin, C. Passow, J. Peschel, D. Ramm, D. Rompotis, N. Schirmel, S. Trippel, J. Wiese, F. Ziaee, S. Bari, M. Burt, J. Küpper, A. M. Rijs, D. Rolles, S. Techert, P. Eng-Johnsson, M. Brouard, C. Vallance, B. Manschwetus, M. Schnell

**Affiliations:** 1 Deutsches Elektronen-Synchrotron DESY, Hamburg, Germany; 2 The Chemistry Research Laboratory, University of Oxford, Oxford, United Kingdom; 3 Institute of Physical Chemistry, Christian-Albrechts-Universität zu Kiel, Kiel, Germany; 4 Department of Physics, Lund University, Lund, Sweden; 5 Physics Department, University of Gothenburg, Gothenburg, Sweden; 6 Center for Ultrafast Imaging, Universität Hamburg, Hamburg, Germany; 7 European XFEL, Schenefeld, Germany; 8 Department of Physics, Universität Hamburg, Hamburg, Germany; 9 Center for Free-Electron Laser Science, Deutsches Elektronen-Synchrotron DESY, Hamburg, Germany; 10 Radboud University, FELIX Laboratory, Nijmegen, The Netherlands; 11 Van’t Hoff Institute for Molecular Sciences, University of Amsterdam, Amsterdam, The Netherlands; 12 Saint Petersburg State University, Saint Petersburg, Russia; 13 Department of Chemistry, Universität Hamburg, Hamburg, Germany; 14 J.R. Macdonald Laboratory, Department of Physics, Kansas State University, Manhattan, KS USA; 15 Division of BioAnalytical Chemistry, Vrije Universiteit Amsterdam, Amsterdam, The Netherlands; 16 Institute for X-Ray Physics, Georg-August-Universität, Göttingen, Germany

**Keywords:** Chemical physics, Reaction kinetics and dynamics, Atomic and molecular interactions with photons

## Abstract

Polycyclic aromatic hydrocarbons (PAHs) play an important role in interstellar chemistry and are subject to high energy photons that can induce excitation, ionization, and fragmentation. Previous studies have demonstrated electronic relaxation of parent PAH monocations over 10–100 femtoseconds as a result of beyond-Born-Oppenheimer coupling between the electronic and nuclear dynamics. Here, we investigate three PAH molecules: fluorene, phenanthrene, and pyrene, using ultrafast XUV and IR laser pulses. Simultaneous measurements of the ion yields, ion momenta, and electron momenta as a function of laser pulse delay allow a detailed insight into the various molecular processes. We report relaxation times for the electronically excited PAH^*^, PAH^+*^ and PAH^2+*^ states, and show the time-dependent conversion between fragmentation pathways. Additionally, using recoil-frame covariance analysis between ion images, we demonstrate that the dissociation of the PAH^2+^ ions favors reaction pathways involving two-body breakup and/or loss of neutral fragments totaling an even number of carbon atoms.

## Abstract (web summary)

Polycyclic aromatic hydrocarbons play an important role in interstellar chemistry, where interaction with high energy photons can induce ionization and fragmentation reactions. Here the authors, with XUV-IR pump-probe experiments, investigate the ultrafast photoinduced dynamics of fluorene, phenanthrene and pyrene, providing insight into their preferred reaction channels.

## Introduction

Polycyclic aromatic hydrocarbons (PAHs) are abundant molecules in the interstellar medium (ISM), accounting for ~10% of the total galactic carbon, according to infrared (IR) emission spectra measured by the Spitzer Space Telescope^1,2^. PAHs undergo a variety of processes upon irradiation, including ionization, dehydrogenation, fragmentation, and isomerization, and the influence of PAHs on the thermodynamics and chemistry of the ISM has motivated laboratory research for many decades^3,4^. The conditions of low temperature, low density, and strong photon radiation mean that these unimolecular reactions become more important compared to a terrestrial environment and are key to the structure and evolution of the ISM^5–7^.

Previous studies into the ionization and fragmentation of PAHs have employed synchrotron and rare gas lamps as photon sources up to the extreme ultraviolet (XUV) and X-ray wavelengths^8–12^. Photoelectron photoion photoion coincidence (PEPIPICO) experiments demonstrated that the interaction of PAHs with XUV photons yielded a PAH^2+^/PAH^+^ ratio of ~0.25 for naphthalene at the highest photon energy of 40.8 eV^13^. These studies also investigated the decay mechanisms of doubly charged PAHs (naphthalene-d_8_, naphthalene-h_8_, and azulene), revealing prominent dissociation channels into two monocations where zero, two, and four carbon atoms were lost in neutral co-fragments^14,15^. Such experiments provide important information on the photofragmentation products and give an insight into molecules that may exist in the ISM but have not yet been assigned by spectral features.

PAHs, with their extended electron system, also provide a framework to explore ultrafast dynamics and beyond-Born-Oppenheimer effects. Within the Born-Oppenheimer approximation, electrons are assumed to move much faster than the nuclei, and the electronic and nuclear frameworks can therefore be treated separately. If the electronic and nuclear motion are instead strongly coupled, this can result in the ultrafast energy transfer from electronic excitation into nuclear motion, and the Born-Oppenheimer approximation breaks down^16–20^. Non-adiabatic electronic relaxation has been demonstrated in recent ultrafast time-resolved studies on a series of small PAHs revealing electronic lifetimes of the monocation in the range 30–55 fs^21^. Theoretical calculations suggest the presence of conical intersections facilitating rapid electronic relaxation. Further experiments indicate that the electronic lifetimes increase with both the molecular size and the cationic excitation energy, and that multi-electronic and non-Born-Oppenheimer effects must be accounted for to provide accurate theoretical calculations^22–24^.

**Fig. 1:**
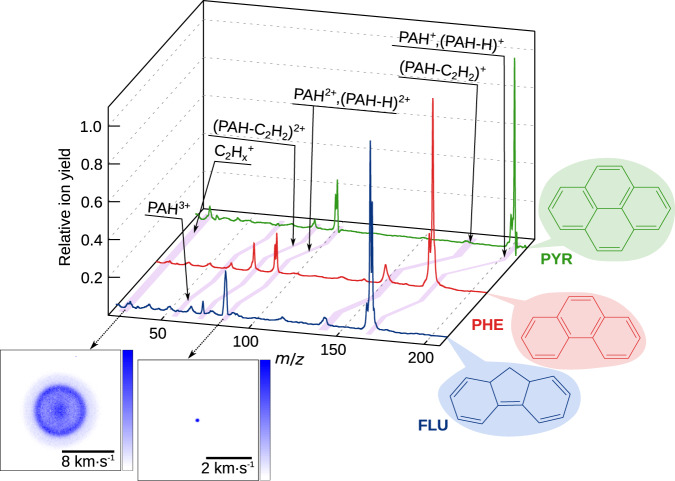
XUV pulse only data. Ion mass spectra for the three PAH molecules, pyrene (PYR), phenanthrene (PHE), and fluorene (FLU), after irradiation with the XUV pulse at 30.3 nm, in green, red, and blue respectively. Intensities are normalized on the height of the respective parent ion PAH^+^. Insets show the velocity-map images for the PAH^2+^ dication and C_2_H${}_{x}^{+}$ for FLU (four-fold symmetrization about the laser polarization and vertical axis applied).

Here, we present femtosecond XUV-IR pump-probe experiments from the free-electron laser (FEL) FLASH in Hamburg^25^, studying the ultrafast photoinduced dynamics of fluorene (FLU), phenanthrene (PHE), and pyrene (PYR). The structures of these molecules are shown in Fig. 1. This comparative study using 30.3 nm (40.9 eV) XUV photons, corresponding to the He II emission line, a dominant spectral line in the interstellar environment, provides valuable insight into the stability and the accessible reaction pathways of these complex molecules^26^. The time-resolved photodynamics of the PAHs are investigated by concurrently recording the photoelectrons using velocity-map imaging (VMI) and the photoions by VMI and time-of-flight (TOF) mass spectrometry. In addition to determining PAH^+*^ excited state lifetimes similar to those reported in the high-harmonic generation (HHG) study by Marciniak et al.^21^, our measurements demonstrate ultrafast relaxation for the PAH^*^ and PAH^2+*^ species. Furthermore, the application of recoil-frame covariance analysis to the ion images provides a detailed mechanistic insight into the fragmentation pathways of the PAH^2+^ ions, demonstrating a strong preference for dissociation into two monocations, where neutral co-fragments contain an even number of carbon atoms^27–31^. Theoretical calculations, reported in Supplementary Notes 1 and 8, support both the electronic relaxation times and the fragmentation channels observed.

## Results

### Time-independent results

The mass spectra obtained following irradiation of FLU, PHE, and PYR with 30.3 nm FEL light are shown in Fig. 1. The spectra are dominated by the cations (PAH^+^) and dications (PAH^2+^) of the respective parents, with small signals from triply ionized molecules also visible. Multiple fragment ions are also seen. The FEL power was set to minimize the formation of PAH^2+^ and PAH^3+^ ions whilst maximizing the PAH^+^ ion yield to avoid multi-photon interactions. In line with the results of previous studies, fragmentation is more pronounced for the two smaller PAHs, FLU and PHE, than for PYR^32^. The parent ion peaks are generally much sharper than the fragment peaks as a result of peak broadening associated with kinetic energy release during the fragmentation process. To illustrate this further, the insets to Fig. 1 show the symmetrized velocity-map ion images for the PAH^2+^ dication and one of the fragmentation products, C_2_H${}_{x}^{+}$, for FLU. The velocity distribution of the dication reflects that of the neutral parent molecule within the molecular beam, which is essentially unaffected by the loss of two electrons, while the fragment ion (C_2_H${}_{x}^{+}$) possesses significant recoil velocity as a result of the kinetic energy released during the parent ion dissociation. The nomenclature of, for example, C_2_H${}_{x}^{+}$ and C_8_H${}_{y}^{+}$, is used throughout this discussion to refer to all monocations with two and eight carbon atoms, respectively, where *x* and *y* encapsulate the possible hydrogen atom values for the fragment.

The velocity-map images provide the 2D projection of the velocity distribution of each detected fragment. Because the velocity distributions for all fragments are recorded simultaneously for each laser pulse, a covariance analysis of the data set allows statistical correlations between the velocities of two or more fragments to be investigated^27^. This provides the correlated velocity distributions of two fragments from the data set, which is a powerful tool for determining the fragmentation processes occurring. The covariance-mapping procedure has been described in previous publications^28–31,33^ and is detailed further in the Methods section. To the authors’ knowledge, the PAHs studied here are the largest molecules that recoil-frame covariance map imaging has been applied to. Briefly, in a covariance image, cov(*A*, *B*), one of the two fragments is chosen to be the ‘reference’ ion (*B*), and the covariance map image reveals the velocity distribution of the ion of interest (*A*) relative to the trajectory direction of the reference ion. In the simplest example, for a parent dication undergoing unimolecular dissociation into two monocations, *A* and *B*, the trajectories of *A* and *B* will always be directly opposed due to conservation of momentum. Therefore, assuming no other formation pathways, the appearance of cov(*A*, *B*) would strongly resemble a single point directly opposite the reference direction. Due to rotational freedom of the molecule in and out of the detector plane, signal leading from the point to the center of the covariance image would also be expected.

**Fig. 2:**
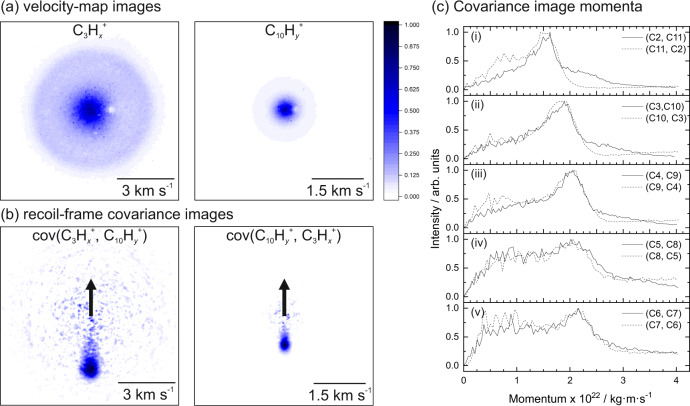
Ion images, covariance images, and momentum comparisons. **a** Velocity-map images and **b** covariance map images for the C_3_H${}_{x}^{+}$ and C_10_H${}_{y}^{+}$ fragment ions arising from dissociative ionization of FLU, C_13_H_10_. Each image is individually normalized. The velocity-map images are presented without symmetrization or Abel-inversion to provide a better comparison to the covariance images. The small areas lacking signal near the center of the velocity-map images are attributed to damage in the MCP/phosphor detector. Black arrows in the covariance images indicate the direction of the reference ion. **c** Momentum profiles of the ion pairs resulting from the two-body dissociative ionization of fluorene. An angular integration is performed on the covariance images, and the spatial coordinates are converted to momentum units. Cov(*A, B*) refers to the covariance of ion *A* with *B* as the reference ion. Discrepancies between the plots in (i) are attributed to physical defects in the detector and background ions affecting the ion images differently.

In this experiment, as hydrogen loss is difficult to resolve using ion imaging (limited by the phosphor screen decay time and the time resolution of the PImMS sensor), the discussion focuses on the fragmentation of the carbon framework. Figure 2a shows the raw velocity-map images recorded for the C_3_H${}_{x}^{+}$ and C_10_H${}_{y}^{+}$ fragments of the dissociative ionization of FLU, C_13_H_10_. The two images potentially contain contributions from the dissociation of singly charged, doubly charged, and more highly charged parent ions. Performing the covariance analysis isolates the fragmentation of the doubly charged parent ion to form two monocations, which corresponds to the higher velocity ring component seen in the velocity-map images. Figure 2b shows the recoil-frame covariance images of C_3_H${}_{x}^{+}$ relative to the C_10_H${}_{y}^{+}$ reference ion (left), and vice versa (right), with black arrows indicating the direction of the reference ion. As expected for a two-body dissociation, the two fragments recoil in opposite directions, i.e., the covariance signal is seen 180^∘^ from the reference direction, due to conservation of momentum, as described above. The relative speeds of the two fragments are determined by their individual masses: the heavier C_10_H${}_{y}^{+}$ recoils more slowly than the lighter C_3_H${}_{x}^{+}$ fragment, yielding a covariance signal closer to the center of the image. Converting the spatial coordinates in the covariance images to momentum units and performing an angular integration gives the momentum profiles shown in Fig. 2(c)(ii). As expected, there is excellent agreement between the momentum profiles obtained from the cov(C_3_H${}_{x}^{+}$,C_10_H${}_{y}^{+}$) and cov(C_10_H${}_{x}^{+}$,C_3_H${}_{y}^{+}$) covariance maps.

**Fig. 3:**
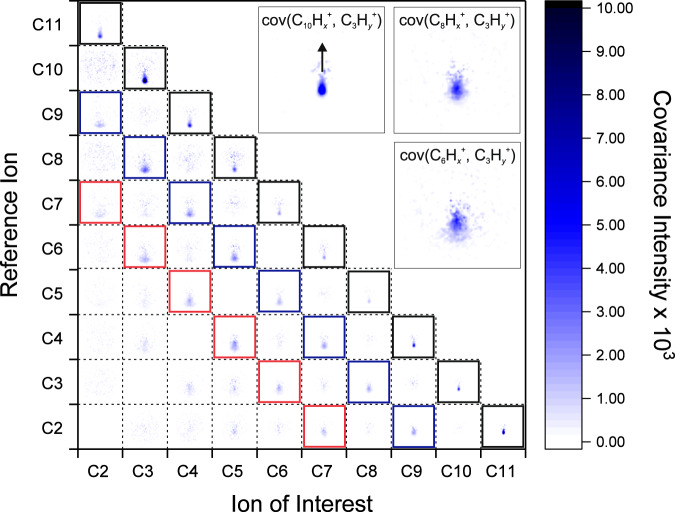
Full recoil-frame covariance map images. The covariance map images between all ion species produced from FLU. Axis labels refer to the number of carbons in the monocationic species and omit the number of hydrogen atoms. Fragment ion pairs that have a total number of carbons greater than that of the parent ion, for example, between C_8_H${}_{x}^{+}$ and C_9_H${}_{x}^{+}$, show no covariance as they cannot be formed as partners from the same parent molecule; these have been omitted for clarity. Any ion species will also always show covariance with itself (autocovariance), and these entries have also been omitted. Notably, signal in the cells along the main diagonal of the matrix (highlighted in black) corresponds to the two-body dissociation pathways from the FLU^2+^ ion. In addition, the cells highlighted in blue and red correspond to the dissociation pathways where two and four carbon atoms, respectively, are lost in neutral co-fragments. Inset: expansions for the maps corresponding to cov(C_10_H${}_{x}^{+}$, C_3_H${}_{y}^{+}$), cov(C_8_H${}_{x}^{+}$, C_3_H${}_{y}^{+}$), cov(C_6_H${}_{x}^{+}$, C_3_H${}_{y}^{+}$) showing increased blurring as more carbon atoms are lost in neutral co-fragments. The black arrow indicates the direction of the reference ion in all covariance maps.

Further to this, the recoil-frame covariance analysis can be performed between all C_*n*_H${}_{x}^{+}$ species for the PAHs. The resulting covariance maps for the FLU fragment ions are plotted as a matrix in Fig. 3, providing a rich basis for elucidating the formation mechanisms for each fragment. The initial focus is on the covariance maps along the main diagonal of the matrix, highlighted by black squares in Fig. 3. These correspond to the two-body dissociations of the doubly charged parent, similar to the reaction FLU^2+^ → C_3_H${}_{x}^{+}$ + C_10_H${}_{y}^{+}$ discussed above. Clear covariance signals can be seen for each of the ion pairs, demonstrating that, except for the loss of a single carbon atom, all two-body dissociation processes of the carbon framework resulting in two monocations are possible from the PAH^2+^ parent. This may seem surprising given the extensive rearrangement of the carbon backbone that must occur for several of the dissociation pathways. Calculated dissociation energies for various channels of PAH^+^ and PAH^2+^, resulting in neutral, singly, and doubly charged fragments, are shown in Supplementary Note 1. These energies were calculated using a rigid approximation for simplicity. Pathways that correspond to a doubly charged parent ion fragmenting into two singly charged ions, for example, C_10_H${}_{7}^{+}$ + C_3_H${}_{3}^{+}$ or C_9_H${}_{6}^{+}$ + C_4_H${}_{4}^{+}$, are predicted to have relatively low dissociation energies (8.3 and 10 eV, respectively), which are energetically consistent with the observation of many such fragmentation channels in the covariance map images. Each of the two-body dissociation processes also shows excellent momentum matching in the covariance maps, as plotted in Fig. 2c(i)-(v).

In addition to seeing covariances between the ion pairs that have masses totaling the molecular mass of the parent ion, prominent covariances are seen between ion pairs that are an even number of carbon units deficient from the parent mass. Ion pairs that are two and four carbon units deficient from the parent mass are highlighted with blue and red squares, respectively, in Fig. 3. The covariance signal between C_3_H${}_{x}^{+}$ and C_8_H${}_{y}^{+}$ in Fig. 3 demonstrates a dissociation pathway that is consistent with the reaction C_13_H${}_{10}^{2+}\,$ → C_3_H${}_{x}^{+}$ + C_8_H${}_{y}^{+}$ + C_2_H_2_, and the velocities of the C_3_H${}_{x}^{+}$ and C_8_H${}_{y}^{+}$ ions match the Coulombic repulsion expected between two monocations. Similarly, the covariance signals between C_3_H${}_{x}^{+}$ and C_6_H${}_{y}^{+}$ demonstrate two-body dissociation from the parent dication with four carbon atoms lost in neutral fragments. Previous PEPIPICO studies investigating the dissociative ionization of PAH molecules at XUV wavelengths have attributed similar observations to the loss of one or more neutral acetylene (C_2_H_2_) molecules^13,14^.

It is observed that as more carbon atoms are lost in neutral molecules, the covariance maps become successively more blurred, e.g., as shown in the insets in Fig. 3 comparing cov(C_10_H${}_{x}^{+}$, C_3_H${}_{y}^{+}$), cov(C_8_H${}_{x}^{+}$, C_3_H${}_{y}^{+}$), and cov(C_6_H${}_{x}^{+}$, C_3_H${}_{y}^{+}$). Assuming that the two carbons are lost as a neutral C_2_H_2_ molecule, this may result either from secondary fragmentation (PAH^2+^ → ${m}_{1}^{+}$ + ${m}_{2}^{+}\,$ → ${m}_{1}^{+}$ + ${m}_{3}^{+}$ + C_2_H_2_) or from deferred charge separation reactions (PAH^2+^ → C_2_H_2_ + ${m}_{1}^{2+}\,$ → C_2_H_2_ +  ${m}_{2}^{+}$ + ${m}_{3}^{+}$). In both cases, blurring in the covariance images would be expected. These pathways would usually be differentiated by observing the gradient of the regression line in an ion-TOF ion-TOF covariance plot^34^, as previously performed for naphthalene-d8, demonstrating deferred charge separation^14^. The ion-TOF ion-TOF partial covariance plots for the PAH molecules in our study are shown in Supplementary Note 2, following correction for the FEL and IR shot-to-shot intensity fluctuations. Some two-body dissociation pathways from the parent dication are visible, but three-body dissociations (two ions and one neutral fragment) cannot be discerned above the noise level, preventing this technique from being applied. There is currently ongoing work exploring the use of the ion images to create an ion-momentum ion-momentum covariance plot to differentiate the dissociation pathways.

The recoil-frame covariance results for PHE and PYR are shown in Supplementary Note 3. These similarly demonstrate a propensity for dissociation pathways from the parent dication into two monocations, with either zero or an even number of carbon atoms lost in neutral co-fragments. The signal levels for PYR^2+^ fragmentation are notably lower than for FLU^2+^ and PHE^2+^, matching previous studies commenting that larger PAH molecules such as PYR tend to form stable parent cations and undergo less dissociative ionization compared to smaller PAHs^35,36^.

### Time-dependent results

**Fig. 4:**
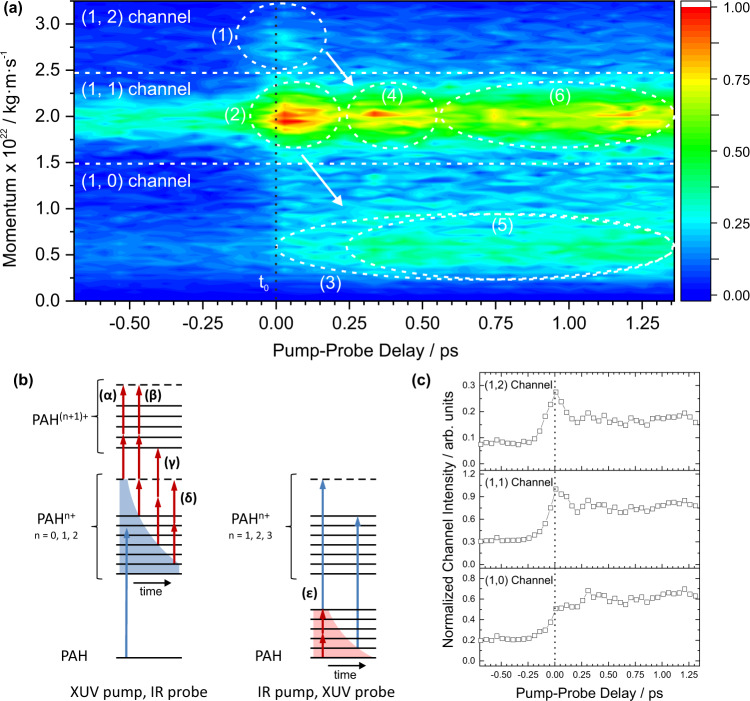
Time-dependent fragmentation channels. **a** The C_3_H${}_{x}^{+}$ momentum profile formed from FLU as a function of XUV-IR laser pulse delay. The IR pulse interacting with the molecular beam before the XUV pulse gives negative pump-probe delay values, whereas for positive pump-probe values, the XUV pulse interacts first. The vertical black dotted line indicates t_0_ determined by the helium electron sidebands. Channels and numbered features are described in detail in the text. Briefly, (1) and (2) show the increase in C_3_H${}_{x}^{+}$ ions formed by dissociative ionization from the PAH^3+^ and PAH^2+^ ion, respectively, around t_0_. (3) shows the increase in C_3_H${}_{x}^{+}$ ion signal after t_0_ due to the XUV pulse creating PAH^+^ ions that are dissociated by the IR pulse. (4) and (5) show the increase in signal in the (1, 1) and (1, 0) channels following relaxation of the PAH^2+^ and PAH^+^ ions, respectively, and dissociation by the IR laser pulse. (6) highlights the general increase in signal after t_0_ compared to before t_0_, even after a long pump-probe delay, in this case, due to the XUV pulse creating PAH^2+^ ions which are dissociated by the IR pulse. White arrows highlight the shift in channel yields from pathway *β* and *γ* to pathway *δ*, as denoted in (**b**). **b** Schematic of the PAH states involved in this experiment. Solid black lines and dashed lines represent bound and dissociative electronic states, respectively. Blue and red arrows represent the effects of the XUV laser and IR laser pulses, respectively. The pump pulses are able to populate a multitude of electronic states, as represented by the blue and red shaded areas for the XUV and IR pulses, respectively. Electronic relaxation of the wavepacket over time is represented by the curvature in the shaded areas. The change in electronic states due to relaxation alters the product of the probe pulse. **c** Integrated plots of the (1, 0), (1, 1), and (1, 2) channels from (**a**). Plots are normalized to the maximum signal detected in the (1, 1) channel, and t_0_ is represented by the dotted black line.

In this section, we present the effect of applying a 30.3 nm XUV pulse and 810 nm pulse at various delays on the three PAHs. This analysis integrates the electron VMI, ion VMI, and ion TOF measurements. Analysis of the helium carrier gas photoelectron sideband lines in the electron images provides an independent and accurate measurement of when the two laser pulses overlap in time (t_0_), outlined in more detail in Supplementary Note 4. For the PAHs in this investigation, there are two major two-color reaction pathways depending on which laser pulse arrives first, as shown in the schematic in Fig. 4b:

- Scenario 1: The XUV pulse interacts with the PAH to produce an electronically excited charged molecule, which can rapidly decay to produce a vibrationally hot parent species. Absorption of one or more IR photons before electronic relaxation can cause electronic excitation and/or promote the molecule to the next charge state. If further ionization is induced, the ejected electron typically has low kinetic energy (LKE), as each IR photon is low in energy. In this analysis, LKE electrons are defined as having a kinetic energy lower than 2 eV, compared to the 1.53 eV of the IR photons. Increasing the delay between the XUV and the IR pulse allows the molecule to electronically relax, thus increasing fragmentation caused by the IR pulse through loss of H, C_2_H_2_ or other neutral or charged species.
- Scenario 2: The IR pulse interacts with the PAH first, producing a vibrationally and electronically excited neutral molecule by multiphoton excitation. In the excited state, absorption of an XUV photon has an increased probability of leading to ionization or dissociative ionization. Evidence for this scenario is demonstrated in the analysis of the time-dependent PAH parent ions described below, where the fits were significantly improved by introducing a channel representing this pathway, i.e., before t_0_. Note that the IR pulse is strong enough to produce PAH^+^ ions directly but in a negligible quantity compared to the XUV pulse. Comparative TOF spectra are shown in Supplementary Note 5.

For all PAHs, the XUV/IR laser regime creates significant quantities of both fragment and parent ions. The delay-dependent yields of these are considered separately in the following sections.

### Fragment Ions

The XUV/IR pump-probe experiments create all possible singly charged ions from the carbon framework with the exception of CH${}_{x}^{+}$, consistent with previous studies^13,14^. For each fragment ion, a velocity-map image was extracted from the PImMS data and Abel-inverted using the polar onion-peeling method^37^. After performing an angular integral, the velocity distribution was converted to momentum and plotted as a function of pump-probe delay. This discussion focuses on the C_3_H${}_{x}^{+}$ ion formation from FLU, shown in Fig. 4a, which had strong signal levels across the three PAHs studied. Momentum plots for C_3_H${}_{x}^{+}$ from PHE and PYR are shown in Supplementary Note 3, and other fragments will be discussed in a follow-up publication. The momentum plot can be divided into three regions:

- high momentum, referred to as the (1, 2) channel, with the C_3_H${}_{x}^{+}$ ion primarily recoiling against a dication.
- medium momentum, referred to as the (1, 1) channel, where C_3_H${}_{x}^{+}$ recoils against a monocation.
- low momentum, referred to as the (1, 0) channel, where there is no charged partner in the dissociation.

The (1, 2) and (1, 1) channels were confirmed by performing a covariance analysis between ion pairs, as described above. The integrated channel yields are plotted in Fig. 4c.

A transient increase in signal around t_0_ is highlighted as feature (1) in Fig. 4a. At negative pump-probe delays, the IR pulse arrives before the XUV pulse, creating vibrationally and electronically excited PAH^*^ molecules. If the pump-probe delay is short (−100 to 0 fs), the PAH^*^ molecules remain in an excited state and the XUV pulse can induce ionization to PAH^*n*+^ (*n* = 1, 2, 3) states, as represented by path *ϵ* in Fig. 4b. Dissociation from the PAH^3+^ states produces fragments in the (1, 2) channel, explaining the increase in signal in feature (1) before t_0_. After t_0_, the XUV pulse precedes the IR pulse, and the XUV-only mass spectrum in Fig. 1 demonstrates that the primary products are PAH^+^ and PAH^2+^ ions. The IR pulse can induce dissociative ionization of the electronically hot PAH^2+*^, producing fragments from PAH^3+^, represented by pathways *α* and *β* in Fig. 4b. This pathway also produces ions in the (1, 2) channel.

Electronic relaxation following the initial laser pulse is represented in Fig. 4b by the curvature in the blue and red shaded areas for the XUV pulse and IR pulse, respectively. Electronically excited PAH^2+*^ molecules produced by the XUV pulse relax during the time of the pump-probe delay; as the delay is increased, more energy is required from the IR pulse to initiate dissociative ionization. Consequently, the (1, 2) channel yield diminishes, and the IR pulse begins to favor dissociating the PAH^2+^ ion instead of promoting it to a PAH^3+^ state. This is represented in Fig. 4b by pathways *α*, *β*, and *γ* converting to pathway *δ* as the molecule relaxes and is similar to the wavepacket evolution identified for the PAH^2+^ ion in a previous HHG study^21^. This causes a signal increase in the (1, 1) channel after ~250 fs, labeled as feature (4) in Fig. 4a.

In the (1, 1) channel, an increase in signal can be seen around t_0_, labeled feature (2). As noted for the (1, 2) channel, prior to t_0_, the IR pulse primarily creates PAH^*^ molecules. Whilst electronically excited, the XUV pulse can more easily promote PAH^*^ to a dissociative PAH^2+^ state, as represented by pathway *ϵ* with *n* = 2. At positive pump-probe delay values, the XUV pulse creates significant quantities of PAH^+^ and PAH^2+^, which are subsequently promoted to a dissociative PAH^2+^ state by the IR pulse. These processes are represented by pathways *β* and *δ* with *n* = 1 and 2, respectively.

Two increases in signal are visible in the (1, 0) channel, one beginning at t_0_, labeled feature (3), and one beginning after ~250 fs, labeled feature (5). Feature (3) is attributed primarily to the XUV pulse creating excited PAH^+*^, which is dissociated by the IR pulse. This is represented by pathway *δ* with *n* = 1. Notably, the signal increase only begins after t_0_, implying that the IR-pump, XUV-probe regime contribution to this channel is negligibly small. Feature (5) has a similar origin to feature (4)—the XUV pulse initially creates an ensemble containing highly excited PAH^+*^ ions, which can be readily promoted to a PAH^2+^ state by the IR laser pulse at short pump-probe delays. However, at longer delay values, the ions electronically relax causing the IR laser to induce dissociation rather than ionization. Again, this can be represented as a shift from pathways *α*, *β*, and *γ* to *δ*.

An overall increase in signal is seen when the IR pulse arrives after the XUV pulse, even for very long pump-probe delays; for example, the signal level in feature (6) compared to the signal before t_0_. This is attributed to the XUV pulse creating excited parent ions PAH^+*^, PAH^2+*^, and PAH^3+*^, which relax to their ground electronic states. The IR pulse induces dissociation in a fraction of the molecules so the PAH^+^, PAH^2+^, and PAH^3+^ ions contribute to the (1, 0), (1, 1), and (1, 2) fragment channels, respectively. This is shown by pathway *δ* in Fig. 4b.

### Parent Ions

**Fig. 5:**
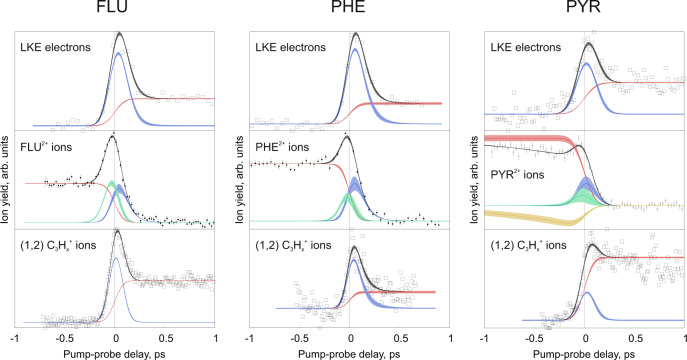
Delay dependent yields and fits. Delay-dependent yields of the LKE electrons, PAH^2+^, and C_3_H${}_{x}^{+}$ ions from the (1, 2) channel formed from FLU, PHE, and PYR. The vertical black line represents t_0_. LKE electrons are defined as having a kinetic energy below 2 eV. Details for the fitting procedure are described in the Methods section. The blue and green peak functions represent the XUV-pump, IR-probe and the IR-pump, XUV-probe laser regimes, respectively. The red sigmoidal curves represent the change in signal due to different PAH ionization states populated by the pump laser. The PYR^2+^ curve additionally contains another peak function (in yellow) to account for slowly changing IR-pump dynamics reproduced over multiple measurements. The black curves show the sum of the peak functions and sigmoidal. Each data point in the PAH^2+^ plots results from averaging 1300 ± 200, 2000 ± 400, and 900 ± 400 measurements for FLU, PHE, and PYR, respectively (variation arises from sorting and filtering according to jitter in the FEL pulse timing).

The delay-dependent formations of all ions can be plotted by integrating the appropriate arrival times of the TOF spectrum recorded from the MCP current. This provides a more accurate quantification of the ion signal compared to the ion images, which are susceptible to saturation, particularly for parent ions, which are always incident in the center of the detector. In addition, the behavior of LKE electrons can be monitored using the electron velocity-map images. Yields for the LKE electrons, the PAH^2+^ parent, and the (1, 2) C_3_H${}_{x}^{+}$ ions extracted from the ion images for FLU, PHE, and PYR are shown in Fig. 5. A Monte Carlo sampling procedure was used to obtain the relaxation times of the excited PAH, PAH^+^, and PAH^2+^ species^38^. The pump pulses are able to populate a multitude of electronic states and the measured relaxation times are those averaged over the populated states following the pump laser pulse that are excited to the relevant ionic or dissociative state by the probe pulse. A summarized description of the analysis is detailed below, and the analysis procedure is described briefly in the Methods section and in full in Supplementary Note 6. The relaxation constants for all PAH species are given in Table 1.

**Table 1:** Electronic decay lifetimes.

Exponential Lifetime/fs				
Charge State	FLU	FLU (Theory)	PHE	PYR
PAH^*^	35 ± 8	54 ± 2	29 ± 17	62 ± 71
PAH^+*^	57 ± 13	42 ± 8	76 ± 14	24 ± 11
PAH^2+*^	17 ± 5	–	74 ± 19	28 ± 10

Exponential electronic decay lifetimes for the various charged parent species. The listed error values refer to the fit errors. The pump laser pulses are expected to populate an ensemble of electronic states, as described and modeled in Supplementary Note 6.

### PAH^+*^ lifetime

When the IR pulse induces ionization, the molecule absorbs IR photons until the ionization energy is reached and the electron is ejected. As each photon is low in energy, the electron typically has LKE, defined in this study as below 2 eV. The relatively low IR intensity (1 × 10^13^ W/cm^2^) prevents any significant contribution from above-threshold ionization, which would produce higher-energy electrons. Figure 1 demonstrates that PAH^+^ is the dominant product after interaction with the XUV pulse. This suggests that the transient increase in the LKE electron yield is primarily due to the ionization of PAH^+*^ to PAH^2+^ by the IR pulse. By fitting the LKE electron yield, the PAH^+*^ lifetime can be extracted for each PAH.

### PAH^*^ lifetime

There are a significant number of possible processes leading to both the creation and depletion of the PAH^2+^ ion. For example, the XUV photon may form PAH^2+*^ directly, which is depleted by further ionization or dissociation by the IR pulse. However, as the yields of the other parent and fragment species are comparatively low, it is reasonable to fit the transient behavior using two major pathways, namely (i) the absorption of an XUV photon to form PAH^+*^ followed by ionization to PAH^2+^ by the IR pulse; and (ii) the IR pulse interacting with the molecule to generate an excited neutral PAH^*^, which is ionized by the XUV pulse to generate PAH^2+^. The transient behavior is therefore dependent on the relaxation times of both the PAH^+*^ and PAH^*^ species. By using the values for t_0_ and the PAH^+*^ relaxation lifetimes determined from the electron data, the fitting procedure can isolate the PAH^*^ relaxation times. The PYR^2+^ trace was found to have a slow decrease in signal at t_0_ (visible in Fig. 5) that was reproduced over multiple scans. An additional peak function was used to fit this trace in order to produce the associated PYR* lifetime.

### PAH^2+*^ lifetime

As discussed above in the Fragment Ions section, the transient signal increase for C_3_H${}_{x}^{+}$ in the (1, 2) channel is dependent upon two pathways. Firstly, before t_0_, the IR pulse creates an excited neutral PAH^*^ species, which undergoes dissociative ionization with an XUV photon to a PAH^3+^ state. Secondly, after t_0_, the XUV laser pulse induces the formation of PAH^2+*^, which the IR laser pulse promotes to a dissociative PAH^3+*^ state. The time dependence of the C_3_H${}_{x}^{+}$ ions in the (1, 2) channel is therefore governed by the lifetimes of the PAH^*^ and PAH^2+*^ states, which can be determined from the fitting procedure. The transient increase showed a good fit using a single Gaussian representing XUV-pump, IR-probe (centered after t_0_), indicating the IR-pump, XUV-probe contribution to be small. Therefore, the (1, 2) channel for C_3_H${}_{x}^{+}$ was fitted using only the XUV-pump, IR-probe channel, allowing the PAH^2+*^ lifetime to be extracted.

### Theoretical model

Extensive theoretical calculations were performed to support the experimental results. These are compared in Table 1. Due to the computational requirements, these studies were restricted to calculating the FLU^*^ and FLU^+*^ electronic lifetimes. The approaches are described in the Methods section and in more detail in Supplementary Note 8.

To calculate the FLU^*^ lifetime, the effect of the IR laser pulse on an ensemble of molecules in various orientations was simulated explicitly. Following this, DFT-based trajectory surface hopping molecular dynamics (TSH-MD) calculations were used to track the dynamics of the states formed over 400 fs. The photoionization cross section of each electronic state was calculated to simulate the XUV laser pulse probing these states. Combining the population of the states and the photoionization cross section allows the time-dependent change in FLU^+^ yield to be estimated at 54 ± 2 fs.

For the FLU^+^ lifetimes, the effect of the XUV pulse on a ground state FLU molecule was simulated. 480 FLU^+^ electronic states can be populated by an XUV photon, of which 180 are high enough in energy to be further ionized to FLU^2+^ by a 810 nm photon. This matches our experimental conditions where the experimental cross-correlation times and electron images indicate that single-photon processes are dominant for the IR probe pulse. Given the high number of states and the complicated potential energy surface topology, a linear vibronic coupling (LVC) model was used to perform TSH-MD simulations over 50 fs. The population that can be ionized by a 810 nm photon decreases as the population decays over time, thus giving rise to a decay constant of 42 ± 8 fs for the FLU^+^ ion.

## Discussion

The PAHs studied are different sizes with different levels of bonding between the carbon rings; nonetheless, ultrafast electronic relaxation in the range 10–100 fs was observed for all molecules in all charge states, indicating that electronic relaxation on this timescale is likely to be ubiquitous across PAHs. Theoretical calculations show that with the high range of accessible electronic states, the populations resulting from IR or XUV excitation undergo ultrafast relaxation within this time range. Our value of 24 ± 11 fs for the PYR^+*^ lifetime (Table 1) roughly matches the measurement of 37 ± 3 fs by Marciniak et al.^21^, with discrepancies primarily attributed to the difference in XUV photon energy (16–20 eV compared to 40.9 eV in this study).

Overall, our results provide a comprehensive view of the electronic and fragmentation dynamics of PAH molecules under irradiation of 30.3 nm photons, corresponding to the He II emission line. The propensity for molecules to fragment with neutral fragments containing zero or an even number of carbon atoms provides valuable information for modeling the ISM, both in terms of the species that might be detected and potential reagents for interstellar chemistry. The abundance of accessible dissociation pathways consistent with the loss of one or more neutral C_2_H_2_ echoes the widely accepted model for PAH growth called the hydrogen abstraction C_2_H_2_ addition (HACA) model^39–41^. Our work showcases the potential for the novel technique of recoil-frame covariance map imaging to unravel the dynamics of large molecules with multiple competing fragmentation channels. Furthermore, the ubiquity of ultrafast relaxation times determined experimentally and theoretically indicates that electronically excited PAHs are generally short lived and such molecules in the ISM can be assumed to be in the electronic ground state. This greatly simplifies interstellar chemical modeling involving PAHs by reducing the number of accessible reaction pathways. Our work demonstrates how ultrafast XUV experiments can be used to simultaneously investigate the dissociation and ionization dynamics of complex systems and provide a more complete picture of the molecular processes.

## Methods

### Experiment

The time-resolved pump-probe experiments were performed at FLASH^42^ using the CFEL-ASG Multi Purpose (CAMP) endstation^43^ at beamline 1 (BL1). The CAMP endstation houses a double-sided VMI spectrometer for simultaneous measurement of the electron and ion kinetic energy and angular distributions. The FEL provided 30.3 nm (40.9 ± 0.4 eV) XUV pulses, which, from the electron pulse durations, were estimated to be 90 fs FWHM with a pulse energy of 14 μJ. Two aluminum filters, thicknesses 100.9 and 423.4 nm, were used to reduce the FEL pulse energy to ~3.3 μJ. In addition, to perform pump-probe measurements, the present study used 60 fs FWHM IR laser pulses at 810 nm. By measuring the IR laser pulse intensity and the focal spot size before the beam enters the chamber, the intensity of the IR laser pulse is estimated to be 1 × 10^13^ W/cm^2^. The intensity was tuned so that the IR laser pulse was strong enough to induce a small level of single ionization of the parent molecules without causing significant fragmentation or double ionization (see Supplementary Note 5). An overview of the laser settings is given in Supplementary Note 7, and the laser is described by Redlin et al.^44^.

The sample molecules FLU (C_13_H_10_, melting point (mp) = 116 ^∘^C), PHE (C_14_H_10_, mp = 101 ^∘^C), and PYR (C_16_H_10_, mp = 145 ^∘^C) were purchased from Sigma-Aldrich with 98% purity and used without further purification. The structures of these molecules are shown in Fig. 1. The samples were placed in an in-vacuum reservoir and heated to ~220–230 ^∘^C to increase their respective vapor pressures. Using helium as a carrier gas (1.5–2 bar backing pressure), the molecules were then introduced into vacuum via a supersonic expansion produced by an Even-Lavie high-temperature pulsed valve^45^, using valve opening times of a few tens of microseconds. The resulting molecular beam was skimmed twice to yield well-collimated pulses of isolated PAH molecules.

In terms of the laser pulses employed, two experiments were performed: (a) irradiating the molecules with the XUV light only, in order to explore its effect on the molecules and to allow the FEL pulse intensity to be tuned to minimize multi-photon effects by the FEL and (b) time-resolved pump-probe experiments employing both the 30.3 nm FEL and 810 nm laser pulses. In the pump-probe experiments, the two laser pulses interacting with the molecules have a defined, adjustable time delay with respect to each other. The pump-probe delay is derived by subtracting the arrival time of the XUV pulse from the arrival time of the IR pulse; hence a positive pump-probe delay refers to the XUV pulse interacting with the molecules first, while a negative pump-probe delay refers to the IR pulse interacting with the molecules first. All pump-probe data were sorted into delay bins according to the bunch arrival monitors of the FLASH FEL in order to account for the FEL jitter^46^.

Ions were velocity-mapped onto a position-sensitive detector comprising a pair of microchannel plates (MCPs) coupled to a P47 phosphor screen^47,48^. The ion TOF spectra were recorded by coupling out the voltage drop at the back side of the MCP with a 2 GHz ADC (ADQ412AC-4G-MTCA) through a resistor-capacitor circuit. The ion images from the phosphor screen were captured by a Pixel Imaging Mass Spectrometry 2 (PImMS2) multi-mass imaging sensor housed within a PImMS camera^49,50^, enabling images to be acquired for all fragment ions on each laser cycle. Data from the PImMS2 sensor was processed to obtain TOF mass spectra and two-dimensional projections of the three-dimensional momentum distribution for each detected product. These were analyzed to extract their kinetic energy and angular distributions^37^. A conversion curve from spatial coordinates to kinetic energy was created by modeling the instrument using SIMION^51^.

Electrons were velocity-mapped onto a separate position-sensitive detector mounted opposite to the ion detector. This comprised a pair of MCPs and a P20 phosphor screen. Electron images on the phosphor screen were recorded with a CCD camera (PIKE F145B). Following background correction, the 2D projections of the electron momentum distributions were fully symmetrized (top/bottom/left/right), corrected for ellipticity, and Abel-inverted to obtain time-resolved electron kinetic energy spectra^52^.

### Recoil-frame covariance analysis

Details of the covariance-mapping procedure have been provided in previous publications^28–31^. Performing this analysis requires the data for each species of interest to be recorded for each experimental cycle, in this case, using the PImMS sensor to record the ion image for each species for every laser pulse. Applying recoil-frame covariance analysis to the ion-imaging data allows the investigation of channels producing two or more cations. Covariance analysis is optimal for regimes such as FLASH1, where the FEL operates at 10  Hz with each laser pulse producing significant quantities of ions (e.g. >100). Conversely, lasers operating in the 1 kHz or faster regime generally produce better results under coincidence conditions, in which fewer than one event is detected per laser pulse.

Briefly, the covariance between two variables *A* and *B* is defined as the product of their deviations from their mean values:

1 $${{{{{{{\rm{cov}}}}}}}}(A,B)=\langle (A-\langle A\rangle )\times (B-\langle B\rangle )\rangle =\langle AB\rangle -\langle A\rangle \langle B\rangle$$

where 〈〉 indicates a mean. Here, *A* and *B* are the velocities of the two fragments of interest, and 〈*AB*〉 and 〈*A*〉〈*B*〉 are referred to as the ‘coincidence image’ and ‘false covariance image’, respectively. The covariance of a two-dimensional ion image with another two-dimensional ion image produces a four-dimensional covariance dataset; however, such a data set is generally difficult to interpret. In practice, for each laser pulse, the signal for ion *A* is rotated relative to the reference ion *B* and summed into the coincidence term. In the common case where more than one *A* or *B* ion associated with that laser pulse is detected, the process is repeated for each *B* ion and a proportionate signal for *A* is added to the covariance image. The false covariance image is constructed in a similar manner using the mean *A* and *B* ion images from the whole data set, i.e., 〈*A*〉 and 〈*B*〉. For each pixel in the 〈*B*〉 image that contains signal, the 〈*A*〉 image is rotated to the reference direction, and a proportional amount of signal is added to the false covariance image. Subtracting the false covariance image from the coincidence image yields the covariance image. The covariance images shown in this study are referred to as ‘positive covariance images’ where negative values have been set to 0 for clarity.

### Fitting of the delay dependent yields

The equations used to derive the PAH^*n*+*^ relaxation lifetimes are given in Supplementary Note 6. Briefly, the functions used to approximate the LKE electrons, PAH^2+^, and (1, 2) C_3_H${}_{x}^{+}$ ions consist of the following components:

- A sigmoidal curve centered at t_0_. t_0_ is independently determined from the He(1s) photoelectron line, described in more detail in Supplementary Note 4. The sigmoidal curves represent the change in the signal due to the pump pulse altering the molecular charge state; for example, as demonstrated in Fig. 5, the yield of PAH^2+^ for the PAHs studied is always greater at a pump-probe delay of −0.5 ps compared to +0.5 ps. Before t_0_, this is interpreted as the IR pulse primarily causing internal excitation and the XUV pulse promoting the excited neutral PAH to PAH^2+^. After t_0_, the XUV pulse interacts with the PAH first and can generate PAH^2+^. Following electronic relaxation to the PAH^2+^ ground state, the IR pulse can induce dissociation causing the PAH^2+^ yield to be depleted.
- A transient peak function representing electronic relaxation following excitation by the XUV-pump pulse, probed by the IR pulse. Specifically, the XUV pulse is expected to initiate ionization and electronic excitation, and the peak function accounts for the change in IR-probe yield as electronic relaxation takes place. The maximum of this transient appears after t_0_.
- A transient peak function representing electronic relaxation following excitation by the IR-pump pulse, probed by the XUV pulse. The IR pulse primarily creates electronically excited neutral PAH molecules; electronic relaxation will change the effect of the XUV pulse. This maximum of this transient appears before t_0_, and it is only used when fitting PAH^2+^.

The model sampled two types of non-linear parameters: relaxation lifetimes of the intermediate PAH species and cross-correlation time, which accounts for the finite duration of the pump and probe pulses, the number of pump and probe photons involved in creating the observed product, and the jitter between the pulses. The parameter distributions were obtained using Monte-Carlo sampling of the likelihood function with the Metropolis algorithm^53^. The sampling procedure also sampled the determined uncertainty in t_0_, with the initial t_0_ value determined by analysis of the helium photoelectrons (see Supplementary Note 4).

### Dissociation energies computations

For all three PAHs, structural optimizations on the ground electronic states of the neutral species were performed. Based on these geometries, we calculated the vertical ionization potentials (VIP) and estimated bounds for the dissociation energy corresponding to reactions of the types ${}^{{m}_{0}}$PAH${}^{{q}_{0}+}{\to }^{{m}_{1}}$(PAH-X)${}^{{q}_{1}+}{+}^{{m}_{2}}$X${}^{{q}_{2}+}$, where *q* and *m* are the charge and multiplicity of the species, X = H, C_2_H_2_, C_3_H_3_, and C_4_H_4_, and (PAH-X) refers to a fragment where the moiety X has been removed from the PAH. For this purpose, single-point energy calculations of the PAHs, (PAH-X), and X fragments at the geometries of the ground state optimized PAHs were performed. All calculations were performed at the UKS-DFT/def2-TZVPP level using the ORCA 4.0 software^54^ with *ω*B97^55^ and M06-2x^56^ functionals. The latter functionals have been shown to reproduce various properties of PAHs and aromatic allotropes of carbon^57,58^.

### Simulation of the beyond-Born-Oppenheimer internal conversion

TSH-MD simulations^59^ of FLU were done with the SHARC software^60–62^ interfaced with Orca 4^54^. All the simulations were performed with or based on calculations at the PBE/def2-SV(P) level of theory^63,64^. Excited states were calculated with TD-DFT at the chosen level of theory using the Tamm-Dancoff approximation (TDA). To speed up the TD-DFT calculations, the resolution-of-identity (RI) approximation was used^65–67^. Simulations of the FLU^+^ dynamics excited to the correlation bands were done using a LVC model^68^ as implemented in SHARC^69^. More details on the correlation bands can be found in Herve et al.^22^. The initial conditions for trajectories were sampled from the Wigner distribution of the ground vibrational state. A full description of the procedure including the calculation of the photoionization cross-sections is available in Supplementary Note 8.

## Supplementary information

Supplementary Information

## Data Availability

The data that support the plots within this paper and other findings of this study are available from the corresponding author (M.S.) upon reasonable request.

## Code availability

New custom codes were used to fit the experimental data and to generate the theoretical results in this manuscript. The source codes are available in the following link: https://stash.desy.de/projects/CFA/repos/pahscriptsanddata/browse.
