# Supplementary material for: Time-resolved relaxation and fragmentation of polycyclic aromatic hydrocarbons investigated in the ultrafast XUV-IR regime
Source: Nat Commun. 2021 Oct 20;12:6107. doi: 10.1038/s41467-021-26193-z (PMC8528970; doi:10.1038/s41467-021-26193-z)
Supplement: Supplementary file 1 — Supplementary Information [file 41467_2021_26193_MOESM1_ESM.pdf]

Supplementary information

J. W. L. Lee,[†,‡,⊥⊥] D. S. Tikhonov,[†,¶,⊥⊥] P. Chopra,[†,¶] S. Maclot,[§,‖] A. L. Steber,[†,¶,⊥] S. Gruet,[†] F. Allum,[‡] R. Boll,[#] X. Cheng,[†] S. Düsterer,[†] B. Erk,[†] D. Garg,[†,@] L. He,[△] D. Heathcote,[‡] M. Johny,[△] M. M. Kazemi,[†] H. Köckert,[‡] J. Lahl,[§] A. K. Lemmens,[∇,††] D. Loru,[†,¶] R. Mason,[‡] E. Müller,[†] T. Mullins,[△] P. Olshin,[‡‡] C. Passow,[†] J. Peschel,[§] D. Ramm,[†] D. Rompotis,[#,†] N. Schirmel,[†] S. Trippel,[△,⊥] J. Wiese,[△,¶¶] F. Ziaee,[§§] S. Bari,[†] M. Burt,[‡] J. Küpper,[△,⊥,@,¶¶] A. M. Rijs,[∇] D. Rolles,[§§] S. Techert,[†,‖‖] P. Eng-Johnsson,[§] M. Brouard,[‡] C. Vallance,[‡] B. Manschwetus,[*,†] and M. Schnell[*,†,¶]

†*Deutsches Elektronen-Synchrotron DESY, Notkestraße 85, 22607 Hamburg, Germany*

‡*The Chemistry Research Laboratory, University of Oxford, United Kingdom*

¶*Institute of Physical Chemistry, Christians-Albrecht-Universität zu Kiel, Kiel, Germany*

§*Atomic Physics, Department of Physics, Lund University, Sweden*

‖*Physics Department, University of Gothenburg, Sweden*

⊥*Center for Ultrafast Imaging, Universität Hamburg, Germany*

#*European XFEL, Schenefeld, Germany*

@*Department of Physics, Universität Hamburg, Germany*

△*Center for Free-Electron Laser Science, Deutsches Elektronen-Synchrotron DESY, Hamburg, Germany*

∇*Radboud University, FELIX Laboratory, The Netherlands*

††*Van't Hoff Institute for Molecular Sciences, University of Amsterdam*

‡‡*Saint Petersburg State University, Saint Petersburg, Russia*

¶¶*Department of Chemistry, Universität Hamburg, Germany*

§§*J.R. Macdonald Laboratory, Department of Physics, Kansas State University, Manhattan, KS, USA*

‖‖*Institute for X-Ray Physics, Georg-August-Universität, Göttingen, Germany*

⊥⊥*These authors contributed equally to this work.*

E-mail: bastian.manschwetus@desy.de; melanie.schnell@desy.de

Contents

1 Dissociation Energies **4**

1.1 Calculations Description 4

1.2 The Lowest Energy Dissociation Channels 5

1.3 Full Calculated Data 8

2 TOF-TOF Partial Covariance Maps **31**

3 PImMS Analysis **33**

3.1 Ion-Ion Recoil Frame Covariances 33

3.2 $C_3H_x^+$ Ion Momentum Maps 36

4 Photoelectron Spectra and t_0 Estimation **37**

5 IR Pulse Only, XUV Pulse Only, and IR-XUV Mass Spectra Comparison **42**

6 Fitting the Pump-Probe Data **43**

7 Laser Settings **52**

8 Trajectory surface hopping molecular dynamics simulations of fluorene **53**

8.1 General considerations 53

8.2 Simulation of NIR pump – XUV probe channel with FLU* as an intermediate 53

8.2.1 Model background 53

8.2.2 Simulation of the NIR laser excitation 54

8.2.3 Calculation of relative photoionization cross-sections of the FLU* . . 56

8.2.4 Decay dynamics of the FLU* 60

8.3 Simulation of XUV pump – NIR probe channel with $FLU^{+,*}$ as an intermediate 61

8.3.1 Model background 61

8.3.2 Decay dynamics of the $FLU^{+,*}$ 63

9 Basic properties of the investigated PAHs **66**

Supplementary References **67**

Supplementary Note 1: Dissociation Energies

1.1 Calculations Description

- Molecular structures of each of the uncharged PAHs in singlet states (FLU, PHE and PYR) were optimized at chosen levels of theory (ri-UKS-DFT/def2-TZVPP, DFT=ωB97,[1] M06-2x[2]). These functionals were chosen since they were shown to be applicable for reproducing various parameters of PAHs and aromatic allotropic structures of carbon.[3,4] All these calculations were performed using the Orca 4 program suite[5] with the RIJCOSX approximation[6,7] utilizing the def2/J auxiliary basis sets.[8]

- We performed three types of single point energy (SPE) calculations for various dissociation positions on the basis of optimized geometries at the same level of theory.
  - PAH at chosen charge+spin state ($^{m}\mathrm{PAH}^{q+}$, where multiplicity is $m = 1$ @ charge $q = 0$, $m = 2$ @ $q = 1$, and $m = 1, 3$ @ $q = 2$).
  - Fragments PAH−X and X (i.e., the same geometry with the removed X fragment and restorer of the molecule X). Charge/spin states combination were the same as above.

  The dissociation energy was computed as

$$E_{\mathrm{diss}} = E_{\mathrm{spe}}(^{m_1}(\mathrm{PAH} - \mathrm{X})^{q_1}) + E_{\mathrm{spe}}(^{m_2}\mathrm{X}^{q_2}) - E_{\mathrm{spe}}(^{m}\mathrm{PAH}^{q}) \ ,$$

  where $q = q_1 + q_2$.

- The given values account only for the total dissociation energy, thus the influence of a possible dissociation barrier is ignored. This may lower the value of the calculated dissociation energies, which should be treated qualitatively rather than quantitatively.

1.2 The Lowest Energy Dissociation Channels

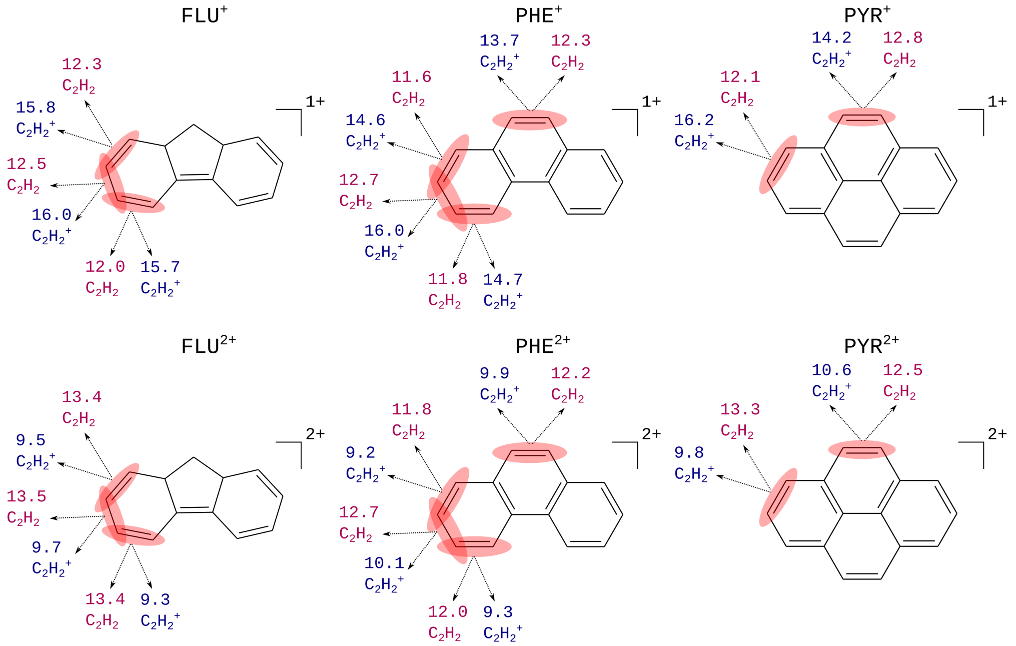

Supplementary Figure 1: Theoretical dissociation energies $E_{\rm diss}$ for acetylene loss from PAH^{n+} (n=1,2) @ ri-UKS-ωB97/def2-TZVPP (all values in eV). All the values were obtained without geometry relaxation.

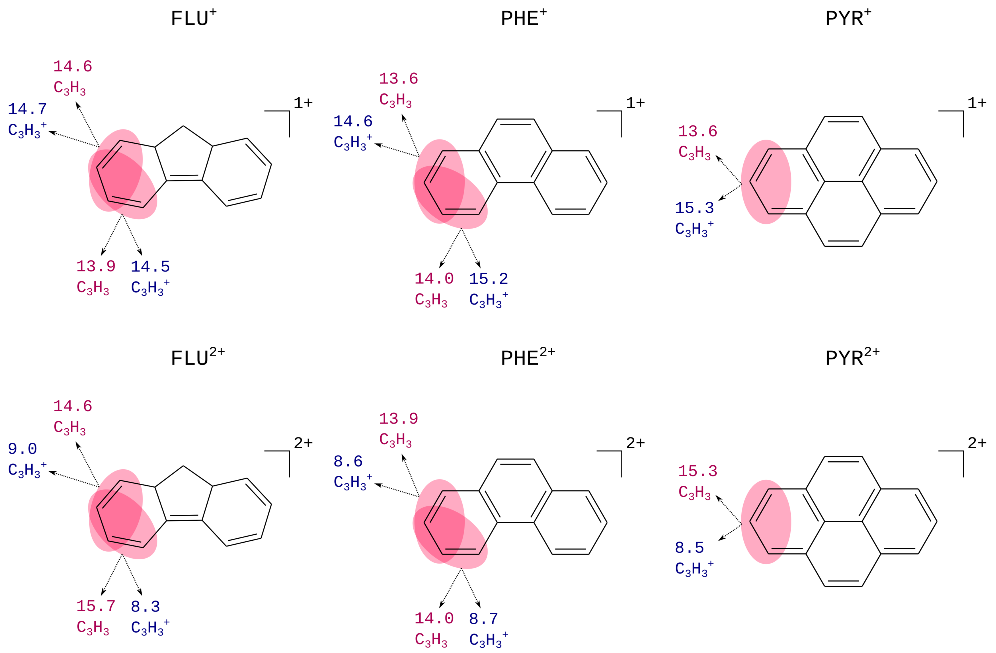

Supplementary Figure 2: Theoretical dissociation energies E_{diss} for C_3H_3 loss from PAH^{n+} (n=1,2) @ ri-UKS-ωB97/def2-TZVPP (all values in eV). All the values were obtained without geometry relaxation.

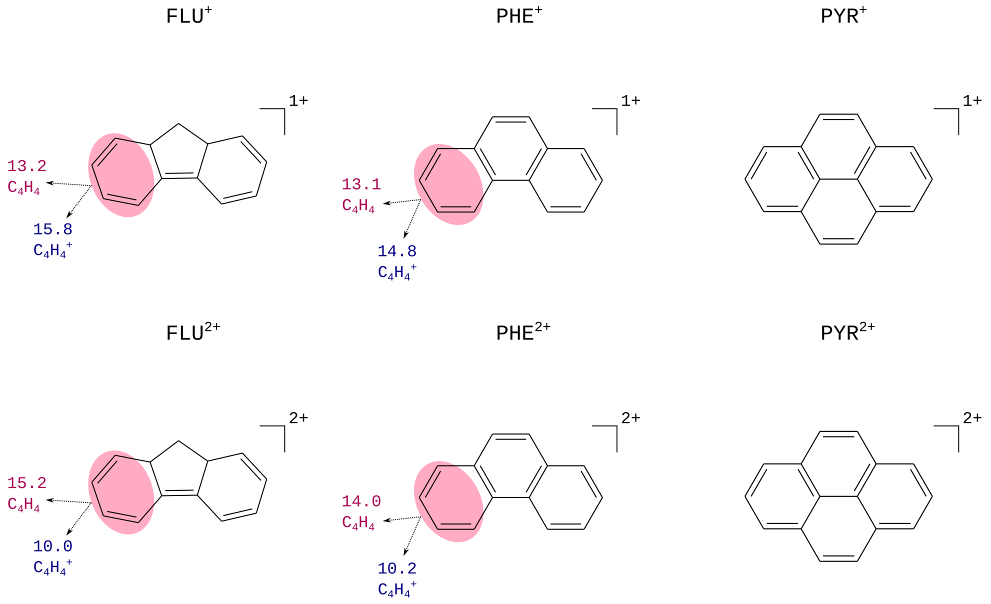

Supplementary Figure 3: Theoretical dissociation energies E_{diss} for C_4H_4 loss from PAH^{n+} (n=1,2) @ ri-UKS-ωB97/def2-TZVPP (all values in eV). All the values were obtained without geometry relaxation.

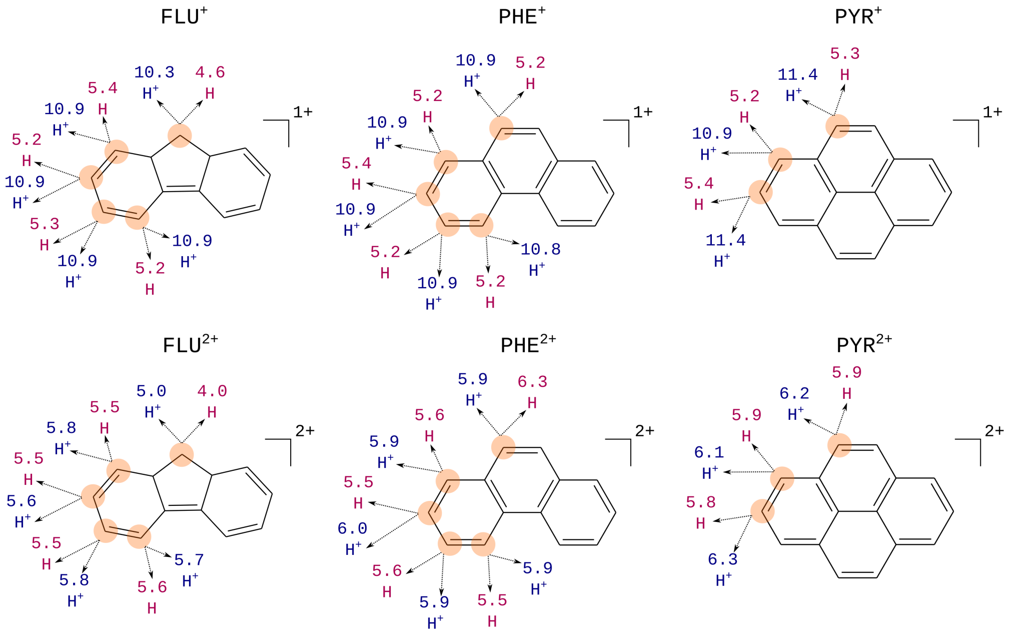

Supplementary Figure 4: Theoretical dissociation energies E_{diss} for proton or hydrogen atom loss from PAH^{n+} (n=1,2) @ ri-UKS-ωB97/def2-TZVPP (all values in eV). All the values were obtained without geometry relaxation.

1.3 Full Calculated Data

Supplementary Table S1: Dissociation energies of acetylene loss from position #1 in FLU @ UKS-DFT/def2-TZVPP level of theory. All values were obtained without geometry relaxation. Bold text highlights the lowest energy dissociation path for a particular PAH charge state and spin state.

Position	Reaction	E_{diss}, eV	
		ωB97	M062x
	$^{1}\mathrm{PAH}^{0+} \rightarrow {}^{1}(\mathrm{PAH}-\mathrm{C_2H_2})^{0+} + {}^{1}\mathrm{C_2H_2^{0+}}$	14.4	13.9
	**$^{1}\mathrm{PAH}^{0+} \rightarrow {}^{3}(\mathrm{PAH}-\mathrm{C_2H_2})^{0+} + {}^{1}\mathrm{C_2H_2^{0+}}$**	**13.3**	**13.2**
	$^{1}\mathrm{PAH}^{0+} \rightarrow {}^{1}(\mathrm{PAH}-\mathrm{C_2H_2})^{0+} + {}^{3}\mathrm{C_2H_2^{0+}}$	14.9	14.5
	$^{1}\mathrm{PAH}^{0+} \rightarrow {}^{3}(\mathrm{PAH}-\mathrm{C_2H_2})^{0+} + {}^{3}\mathrm{C_2H_2^{0+}}$	13.8	13.8
	**$^{2}\mathrm{PAH}^{1+} \rightarrow {}^{2}(\mathrm{PAH}-\mathrm{C_2H_2})^{1+} + {}^{1}\mathrm{C_2H_2^{0+}}$**	**12.3**	**11.9**
	$^{2}\mathrm{PAH}^{1+} \rightarrow {}^{2}(\mathrm{PAH}-\mathrm{C_2H_2})^{1+} + {}^{3}\mathrm{C_2H_2^{0+}}$	12.8	12.6
	$^{2}\mathrm{PAH}^{1+} \rightarrow {}^{1}(\mathrm{PAH}-\mathrm{C_2H_2})^{0+} + {}^{2}\mathrm{C_2H_2^{1+}}$	16.9	16.4
	$^{2}\mathrm{PAH}^{1+} \rightarrow {}^{3}(\mathrm{PAH}-\mathrm{C_2H_2})^{0+} + {}^{2}\mathrm{C_2H_2^{1+}}$	15.8	15.7
	$^{1}\mathrm{PAH}^{2+} \rightarrow {}^{1}(\mathrm{PAH}-\mathrm{C_2H_2})^{2+} + {}^{1}\mathrm{C_2H_2^{0+}}$	14.1	13.7
	$^{1}\mathrm{PAH}^{2+} \rightarrow {}^{3}(\mathrm{PAH}-\mathrm{C_2H_2})^{2+} + {}^{1}\mathrm{C_2H_2^{0+}}$	13.4	12.0
	$^{1}\mathrm{PAH}^{2+} \rightarrow {}^{1}(\mathrm{PAH}-\mathrm{C_2H_2})^{2+} + {}^{3}\mathrm{C_2H_2^{0+}}$	14.7	14.3
	$^{1}\mathrm{PAH}^{2+} \rightarrow {}^{3}(\mathrm{PAH}-\mathrm{C_2H_2})^{2+} + {}^{3}\mathrm{C_2H_2^{0+}}$	13.9	12.7
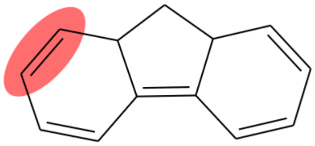	**$^{1}\mathrm{PAH}^{2+} \rightarrow {}^{2}(\mathrm{PAH}-\mathrm{C_2H_2})^{1+} + {}^{2}\mathrm{C_2H_2^{1+}}$**	**9.5**	**9.4**
	$^{1}\mathrm{PAH}^{2+} \rightarrow {}^{1}(\mathrm{PAH}-\mathrm{C_2H_2})^{0+} + {}^{1}\mathrm{C_2H_2^{2+}}$	24.5	24.2
	$^{1}\mathrm{PAH}^{2+} \rightarrow {}^{3}(\mathrm{PAH}-\mathrm{C_2H_2})^{0+} + {}^{1}\mathrm{C_2H_2^{2+}}$	23.3	23.6
	$^{1}\mathrm{PAH}^{2+} \rightarrow {}^{1}(\mathrm{PAH}-\mathrm{C_2H_2})^{0+} + {}^{3}\mathrm{C_2H_2^{2+}}$	23.2	22.6
	$^{1}\mathrm{PAH}^{2+} \rightarrow {}^{3}(\mathrm{PAH}-\mathrm{C_2H_2})^{0+} + {}^{3}\mathrm{C_2H_2^{2+}}$	22.0	21.9
	$^{3}\mathrm{PAH}^{2+} \rightarrow {}^{1}(\mathrm{PAH}-\mathrm{C_2H_2})^{2+} + {}^{1}\mathrm{C_2H_2^{0+}}$	14.3	13.6
	$^{3}\mathrm{PAH}^{2+} \rightarrow {}^{3}(\mathrm{PAH}-\mathrm{C_2H_2})^{2+} + {}^{1}\mathrm{C_2H_2^{0+}}$	13.6	11.9
	$^{3}\mathrm{PAH}^{2+} \rightarrow {}^{1}(\mathrm{PAH}-\mathrm{C_2H_2})^{2+} + {}^{3}\mathrm{C_2H_2^{0+}}$	14.8	14.2
	$^{3}\mathrm{PAH}^{2+} \rightarrow {}^{3}(\mathrm{PAH}-\mathrm{C_2H_2})^{2+} + {}^{3}\mathrm{C_2H_2^{0+}}$	14.1	12.6
	**$^{3}\mathrm{PAH}^{2+} \rightarrow {}^{2}(\mathrm{PAH}-\mathrm{C_2H_2})^{1+} + {}^{2}\mathrm{C_2H_2^{1+}}$**	**9.7**	**9.3**
	$^{3}\mathrm{PAH}^{2+} \rightarrow {}^{1}(\mathrm{PAH}-\mathrm{C_2H_2})^{0+} + {}^{1}\mathrm{C_2H_2^{2+}}$	24.6	24.1
	$^{3}\mathrm{PAH}^{2+} \rightarrow {}^{3}(\mathrm{PAH}-\mathrm{C_2H_2})^{0+} + {}^{1}\mathrm{C_2H_2^{2+}}$	23.5	23.4
	$^{3}\mathrm{PAH}^{2+} \rightarrow {}^{1}(\mathrm{PAH}-\mathrm{C_2H_2})^{0+} + {}^{3}\mathrm{C_2H_2^{2+}}$	23.3	22.5
	$^{3}\mathrm{PAH}^{2+} \rightarrow {}^{3}(\mathrm{PAH}-\mathrm{C_2H_2})^{0+} + {}^{3}\mathrm{C_2H_2^{2+}}$	22.2	21.8

Supplementary Table S2: Dissociation energies of acetylene loss from position #2 in FLU @ UKS-DFT/def2-TZVPP level of theory. All values were obtained without geometry relaxation. Bold text highlights the lowest energy dissociation path for a particular PAH charge state and spin state.

Position	Reaction	E_{diss}, eV	
		ωB97	M062x
	$^1PAH^{0+} \rightarrow {}^1(PAH - C_2H_2)^{0+} + {}^1C_2H_2^{0+}$	14.6	14.1
	**$^1PAH^{0+} \rightarrow {}^3(PAH - C_2H_2)^{0+} + {}^1C_2H_2^{0+}$**	**13.5**	**13.5**
	$^1PAH^{0+} \rightarrow {}^1(PAH - C_2H_2)^{0+} + {}^3C_2H_2^{0+}$	15.1	14.8
	$^1PAH^{0+} \rightarrow {}^3(PAH - C_2H_2)^{0+} + {}^3C_2H_2^{0+}$	14.0	14.2
	**$^2PAH^{1+} \rightarrow {}^2(PAH - C_2H_2)^{1+} + {}^1C_2H_2^{0+}$**	**12.5**	**12.1**
	$^2PAH^{1+} \rightarrow {}^2(PAH - C_2H_2)^{1+} + {}^3C_2H_2^{0+}$	13.0	12.7
	$^2PAH^{1+} \rightarrow {}^1(PAH - C_2H_2)^{0+} + {}^2C_2H_2^{1+}$	17.2	16.6
	$^2PAH^{1+} \rightarrow {}^3(PAH - C_2H_2)^{0+} + {}^2C_2H_2^{1+}$	16.0	16.0
	$^1PAH^{2+} \rightarrow {}^1(PAH - C_2H_2)^{2+} + {}^1C_2H_2^{0+}$	14.8	14.4
	$^1PAH^{2+} \rightarrow {}^3(PAH - C_2H_2)^{2+} + {}^1C_2H_2^{0+}$	13.5	12.0
	$^1PAH^{2+} \rightarrow {}^1(PAH - C_2H_2)^{2+} + {}^3C_2H_2^{0+}$	15.3	15.0
	$^1PAH^{2+} \rightarrow {}^3(PAH - C_2H_2)^{2+} + {}^3C_2H_2^{0+}$	14.0	12.6
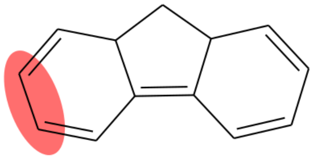	**$^1PAH^{2+} \rightarrow {}^2(PAH - C_2H_2)^{1+} + {}^2C_2H_2^{1+}$**	**9.7**	**9.5**
	$^1PAH^{2+} \rightarrow {}^1(PAH - C_2H_2)^{0+} + {}^1C_2H_2^{2+}$	24.7	24.5
	$^1PAH^{2+} \rightarrow {}^3(PAH - C_2H_2)^{0+} + {}^1C_2H_2^{2+}$	23.6	23.9
	$^1PAH^{2+} \rightarrow {}^1(PAH - C_2H_2)^{0+} + {}^3C_2H_2^{2+}$	23.4	22.8
	$^1PAH^{2+} \rightarrow {}^3(PAH - C_2H_2)^{0+} + {}^3C_2H_2^{2+}$	22.3	22.2
	$^3PAH^{2+} \rightarrow {}^1(PAH - C_2H_2)^{2+} + {}^1C_2H_2^{0+}$	15.0	14.3
	$^3PAH^{2+} \rightarrow {}^3(PAH - C_2H_2)^{2+} + {}^1C_2H_2^{0+}$	13.6	11.9
	$^3PAH^{2+} \rightarrow {}^1(PAH - C_2H_2)^{2+} + {}^3C_2H_2^{0+}$	15.5	14.9
	$^3PAH^{2+} \rightarrow {}^3(PAH - C_2H_2)^{2+} + {}^3C_2H_2^{0+}$	14.1	12.5
	**$^3PAH^{2+} \rightarrow {}^2(PAH - C_2H_2)^{1+} + {}^2C_2H_2^{1+}$**	**9.9**	**9.4**
	$^3PAH^{2+} \rightarrow {}^1(PAH - C_2H_2)^{0+} + {}^1C_2H_2^{2+}$	24.9	24.4
	$^3PAH^{2+} \rightarrow {}^3(PAH - C_2H_2)^{0+} + {}^1C_2H_2^{2+}$	23.7	23.8
	$^3PAH^{2+} \rightarrow {}^1(PAH - C_2H_2)^{0+} + {}^3C_2H_2^{2+}$	23.6	22.7
	$^3PAH^{2+} \rightarrow {}^3(PAH - C_2H_2)^{0+} + {}^3C_2H_2^{2+}$	22.5	22.1

Supplementary Table S3: Dissociation energies of acetylene loss from position #3 in FLU @ UKS-DFT/def2-TZVPP level of theory. All values were obtained without geometry relaxation. Bold text highlights the lowest energy dissociation path for a particular PAH charge state and spin state.

Position	Reaction	E_{diss}, eV	
		ωB97	M062x
	$^{1}\mathrm{PAH}^{0+} \rightarrow {}^{1}(\mathrm{PAH}-\mathrm{C_2H_2})^{0+} + {}^{1}\mathrm{C_2H_2^{0+}}$	14.3	13.8
	**$^{1}\mathrm{PAH}^{0+} \rightarrow {}^{3}(\mathrm{PAH}-\mathrm{C_2H_2})^{0+} + {}^{1}\mathrm{C_2H_2^{0+}}$**	**13.1**	**13.1**
	$^{1}\mathrm{PAH}^{0+} \rightarrow {}^{1}(\mathrm{PAH}-\mathrm{C_2H_2})^{0+} + {}^{3}\mathrm{C_2H_2^{0+}}$	14.9	14.4
	$^{1}\mathrm{PAH}^{0+} \rightarrow {}^{3}(\mathrm{PAH}-\mathrm{C_2H_2})^{0+} + {}^{3}\mathrm{C_2H_2^{0+}}$	13.7	13.8
	**$^{2}\mathrm{PAH}^{1+} \rightarrow {}^{2}(\mathrm{PAH}-\mathrm{C_2H_2})^{1+} + {}^{1}\mathrm{C_2H_2^{0+}}$**	**12.0**	**11.7**
	$^{2}\mathrm{PAH}^{1+} \rightarrow {}^{2}(\mathrm{PAH}-\mathrm{C_2H_2})^{1+} + {}^{3}\mathrm{C_2H_2^{0+}}$	12.6	12.4
	$^{2}\mathrm{PAH}^{1+} \rightarrow {}^{1}(\mathrm{PAH}-\mathrm{C_2H_2})^{0+} + {}^{2}\mathrm{C_2H_2^{1+}}$	16.9	16.3
	$^{2}\mathrm{PAH}^{1+} \rightarrow {}^{3}(\mathrm{PAH}-\mathrm{C_2H_2})^{0+} + {}^{2}\mathrm{C_2H_2^{1+}}$	15.7	15.6
	$^{1}\mathrm{PAH}^{2+} \rightarrow {}^{1}(\mathrm{PAH}-\mathrm{C_2H_2})^{2+} + {}^{1}\mathrm{C_2H_2^{0+}}$	13.7	13.2
	$^{1}\mathrm{PAH}^{2+} \rightarrow {}^{3}(\mathrm{PAH}-\mathrm{C_2H_2})^{2+} + {}^{1}\mathrm{C_2H_2^{0+}}$	13.4	13.2
	$^{1}\mathrm{PAH}^{2+} \rightarrow {}^{1}(\mathrm{PAH}-\mathrm{C_2H_2})^{2+} + {}^{3}\mathrm{C_2H_2^{0+}}$	14.3	13.8
	$^{1}\mathrm{PAH}^{2+} \rightarrow {}^{3}(\mathrm{PAH}-\mathrm{C_2H_2})^{2+} + {}^{3}\mathrm{C_2H_2^{0+}}$	13.9	13.8
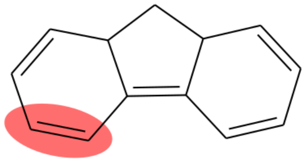	**$^{1}\mathrm{PAH}^{2+} \rightarrow {}^{2}(\mathrm{PAH}-\mathrm{C_2H_2})^{1+} + {}^{2}\mathrm{C_2H_2^{1+}}$**	**9.3**	**9.2**
	$^{1}\mathrm{PAH}^{2+} \rightarrow {}^{1}(\mathrm{PAH}-\mathrm{C_2H_2})^{0+} + {}^{1}\mathrm{C_2H_2^{2+}}$	24.4	24.1
	$^{1}\mathrm{PAH}^{2+} \rightarrow {}^{3}(\mathrm{PAH}-\mathrm{C_2H_2})^{0+} + {}^{1}\mathrm{C_2H_2^{2+}}$	23.2	23.5
	$^{1}\mathrm{PAH}^{2+} \rightarrow {}^{1}(\mathrm{PAH}-\mathrm{C_2H_2})^{0+} + {}^{3}\mathrm{C_2H_2^{2+}}$	23.1	22.5
	$^{1}\mathrm{PAH}^{2+} \rightarrow {}^{3}(\mathrm{PAH}-\mathrm{C_2H_2})^{0+} + {}^{3}\mathrm{C_2H_2^{2+}}$	21.9	21.8
	$^{3}\mathrm{PAH}^{2+} \rightarrow {}^{1}(\mathrm{PAH}-\mathrm{C_2H_2})^{2+} + {}^{1}\mathrm{C_2H_2^{0+}}$	13.9	13.1
	$^{3}\mathrm{PAH}^{2+} \rightarrow {}^{3}(\mathrm{PAH}-\mathrm{C_2H_2})^{2+} + {}^{1}\mathrm{C_2H_2^{0+}}$	13.5	13.0
	$^{3}\mathrm{PAH}^{2+} \rightarrow {}^{1}(\mathrm{PAH}-\mathrm{C_2H_2})^{2+} + {}^{3}\mathrm{C_2H_2^{0+}}$	14.4	13.7
	$^{3}\mathrm{PAH}^{2+} \rightarrow {}^{3}(\mathrm{PAH}-\mathrm{C_2H_2})^{2+} + {}^{3}\mathrm{C_2H_2^{0+}}$	14.1	13.7
	**$^{3}\mathrm{PAH}^{2+} \rightarrow {}^{2}(\mathrm{PAH}-\mathrm{C_2H_2})^{1+} + {}^{2}\mathrm{C_2H_2^{1+}}$**	**9.4**	**9.1**
	$^{3}\mathrm{PAH}^{2+} \rightarrow {}^{1}(\mathrm{PAH}-\mathrm{C_2H_2})^{0+} + {}^{1}\mathrm{C_2H_2^{2+}}$	24.6	24.0
	$^{3}\mathrm{PAH}^{2+} \rightarrow {}^{3}(\mathrm{PAH}-\mathrm{C_2H_2})^{0+} + {}^{1}\mathrm{C_2H_2^{2+}}$	23.4	23.3
	$^{3}\mathrm{PAH}^{2+} \rightarrow {}^{1}(\mathrm{PAH}-\mathrm{C_2H_2})^{0+} + {}^{3}\mathrm{C_2H_2^{2+}}$	23.3	22.4
	$^{3}\mathrm{PAH}^{2+} \rightarrow {}^{3}(\mathrm{PAH}-\mathrm{C_2H_2})^{0+} + {}^{3}\mathrm{C_2H_2^{2+}}$	22.1	21.7

Supplementary Table S4: Dissociation energies of acetylene loss from position #1 in PHE @ UKS-DFT/def2-TZVPP level of theory. All values were obtained without geometry relaxation. Bold text highlights the lowest energy dissociation path for a particular PAH charge state and spin state.

Position	Reaction	E_{diss}, eV	
		ωB97	M062x
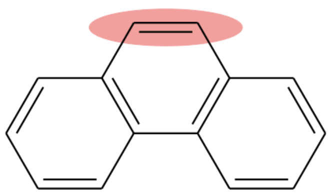	${}^{1}\text{PAH}^{0+} \rightarrow {}^{1}(\text{PAH} - \text{C}_2\text{H}_2)^{0+} + {}^{1}\text{C}_2\text{H}_2^{0+}$	12.6	12.1
	**${}^{1}\text{PAH}^{0+} \rightarrow {}^{3}(\text{PAH} - \text{C}_2\text{H}_2)^{0+} + {}^{1}\text{C}_2\text{H}_2^{0+}$**	**11.0**	**10.9**
	${}^{1}\text{PAH}^{0+} \rightarrow {}^{1}(\text{PAH} - \text{C}_2\text{H}_2)^{0+} + {}^{3}\text{C}_2\text{H}_2^{0+}$	13.5	13.1
	${}^{1}\text{PAH}^{0+} \rightarrow {}^{3}(\text{PAH} - \text{C}_2\text{H}_2)^{0+} + {}^{3}\text{C}_2\text{H}_2^{0+}$	12.0	11.9
	**${}^{2}\text{PAH}^{1+} \rightarrow {}^{2}(\text{PAH} - \text{C}_2\text{H}_2)^{1+} + {}^{1}\text{C}_2\text{H}_2^{0+}$**	**12.3**	**11.8**
	${}^{2}\text{PAH}^{1+} \rightarrow {}^{2}(\text{PAH} - \text{C}_2\text{H}_2)^{1+} + {}^{3}\text{C}_2\text{H}_2^{0+}$	13.2	12.8
	${}^{2}\text{PAH}^{1+} \rightarrow {}^{1}(\text{PAH} - \text{C}_2\text{H}_2)^{0+} + {}^{2}\text{C}_2\text{H}_2^{1+}$	15.2	14.8
	${}^{2}\text{PAH}^{1+} \rightarrow {}^{3}(\text{PAH} - \text{C}_2\text{H}_2)^{0+} + {}^{2}\text{C}_2\text{H}_2^{1+}$	13.7	13.5
	${}^{1}\text{PAH}^{2+} \rightarrow {}^{1}(\text{PAH} - \text{C}_2\text{H}_2)^{2+} + {}^{1}\text{C}_2\text{H}_2^{0+}$	13.0	13.0
	${}^{1}\text{PAH}^{2+} \rightarrow {}^{3}(\text{PAH} - \text{C}_2\text{H}_2)^{2+} + {}^{1}\text{C}_2\text{H}_2^{0+}$	12.2	12.0
	${}^{1}\text{PAH}^{2+} \rightarrow {}^{1}(\text{PAH} - \text{C}_2\text{H}_2)^{2+} + {}^{3}\text{C}_2\text{H}_2^{0+}$	13.9	13.9
	${}^{1}\text{PAH}^{2+} \rightarrow {}^{3}(\text{PAH} - \text{C}_2\text{H}_2)^{2+} + {}^{3}\text{C}_2\text{H}_2^{0+}$	13.1	13.0
	**${}^{1}\text{PAH}^{2+} \rightarrow {}^{2}(\text{PAH} - \text{C}_2\text{H}_2)^{1+} + {}^{2}\text{C}_2\text{H}_2^{1+}$**	**9.9**	**9.7**
	${}^{1}\text{PAH}^{2+} \rightarrow {}^{1}(\text{PAH} - \text{C}_2\text{H}_2)^{0+} + {}^{1}\text{C}_2\text{H}_2^{2+}$	23.2	23.1
	${}^{1}\text{PAH}^{2+} \rightarrow {}^{3}(\text{PAH} - \text{C}_2\text{H}_2)^{0+} + {}^{1}\text{C}_2\text{H}_2^{2+}$	21.7	21.9
	${}^{1}\text{PAH}^{2+} \rightarrow {}^{1}(\text{PAH} - \text{C}_2\text{H}_2)^{0+} + {}^{3}\text{C}_2\text{H}_2^{2+}$	22.0	21.5
	${}^{1}\text{PAH}^{2+} \rightarrow {}^{3}(\text{PAH} - \text{C}_2\text{H}_2)^{0+} + {}^{3}\text{C}_2\text{H}_2^{2+}$	20.5	20.3
	${}^{3}\text{PAH}^{2+} \rightarrow {}^{1}(\text{PAH} - \text{C}_2\text{H}_2)^{2+} + {}^{1}\text{C}_2\text{H}_2^{0+}$	13.5	13.2
	${}^{3}\text{PAH}^{2+} \rightarrow {}^{3}(\text{PAH} - \text{C}_2\text{H}_2)^{2+} + {}^{1}\text{C}_2\text{H}_2^{0+}$	12.8	12.2
	${}^{3}\text{PAH}^{2+} \rightarrow {}^{1}(\text{PAH} - \text{C}_2\text{H}_2)^{2+} + {}^{3}\text{C}_2\text{H}_2^{0+}$	14.4	14.2
	${}^{3}\text{PAH}^{2+} \rightarrow {}^{3}(\text{PAH} - \text{C}_2\text{H}_2)^{2+} + {}^{3}\text{C}_2\text{H}_2^{0+}$	13.7	13.2
	**${}^{3}\text{PAH}^{2+} \rightarrow {}^{2}(\text{PAH} - \text{C}_2\text{H}_2)^{1+} + {}^{2}\text{C}_2\text{H}_2^{1+}$**	**10.4**	**9.9**
	${}^{3}\text{PAH}^{2+} \rightarrow {}^{1}(\text{PAH} - \text{C}_2\text{H}_2)^{0+} + {}^{1}\text{C}_2\text{H}_2^{2+}$	23.7	23.3
	${}^{3}\text{PAH}^{2+} \rightarrow {}^{3}(\text{PAH} - \text{C}_2\text{H}_2)^{0+} + {}^{1}\text{C}_2\text{H}_2^{2+}$	22.2	22.1
	${}^{3}\text{PAH}^{2+} \rightarrow {}^{1}(\text{PAH} - \text{C}_2\text{H}_2)^{0+} + {}^{3}\text{C}_2\text{H}_2^{2+}$	22.5	21.8
	${}^{3}\text{PAH}^{2+} \rightarrow {}^{3}(\text{PAH} - \text{C}_2\text{H}_2)^{0+} + {}^{3}\text{C}_2\text{H}_2^{2+}$	21.0	20.6

Supplementary Table S5: Dissociation energies of acetylene loss from position #2 in PHE @ UKS-DFT/def2-TZVPP level of theory. All values were obtained without geometry relaxation. Bold text highlights the lowest energy dissociation path for a particular PAH charge state and spin state.

Position	Reaction	E_{diss}, eV	
		ωB97	M062x
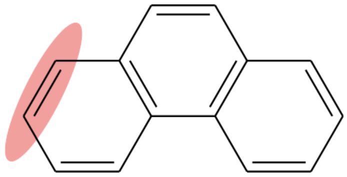	$^1PAH^{0+} \rightarrow {}^1(PAH - C_2H_2)^{0+} + {}^1C_2H_2^{0+}$	13.6	13.1
	$\mathbf{^1PAH^{0+} \rightarrow {}^3(PAH - C_2H_2)^{0+} + {}^1C_2H_2^{0+}}$	**12.1**	**11.8**
	$^1PAH^{0+} \rightarrow {}^1(PAH - C_2H_2)^{0+} + {}^3C_2H_2^{0+}$	14.3	13.9
	$^1PAH^{0+} \rightarrow {}^3(PAH - C_2H_2)^{0+} + {}^3C_2H_2^{0+}$	12.8	12.6
	$\mathbf{^2PAH^{1+} \rightarrow {}^2(PAH - C_2H_2)^{1+} + {}^1C_2H_2^{0+}}$	**11.6**	**11.8**
	$^2PAH^{1+} \rightarrow {}^2(PAH - C_2H_2)^{1+} + {}^3C_2H_2^{0+}$	12.3	12.6
	$^2PAH^{1+} \rightarrow {}^1(PAH - C_2H_2)^{0+} + {}^2C_2H_2^{1+}$	16.2	15.7
	$^2PAH^{1+} \rightarrow {}^3(PAH - C_2H_2)^{0+} + {}^2C_2H_2^{1+}$	14.6	14.4
	$^1PAH^{2+} \rightarrow {}^1(PAH - C_2H_2)^{2+} + {}^1C_2H_2^{0+}$	13.6	13.2
	$^1PAH^{2+} \rightarrow {}^3(PAH - C_2H_2)^{2+} + {}^1C_2H_2^{0+}$	11.8	11.3
	$^1PAH^{2+} \rightarrow {}^1(PAH - C_2H_2)^{2+} + {}^3C_2H_2^{0+}$	14.3	14.0
	$^1PAH^{2+} \rightarrow {}^3(PAH - C_2H_2)^{2+} + {}^3C_2H_2^{0+}$	12.5	12.1
	$\mathbf{^1PAH^{2+} \rightarrow {}^2(PAH - C_2H_2)^{1+} + {}^2C_2H_2^{1+}}$	**9.2**	**9.6**
	$^1PAH^{2+} \rightarrow {}^1(PAH - C_2H_2)^{0+} + {}^1C_2H_2^{2+}$	24.1	23.9
	$^1PAH^{2+} \rightarrow {}^3(PAH - C_2H_2)^{0+} + {}^1C_2H_2^{2+}$	22.5	22.6
	$^1PAH^{2+} \rightarrow {}^1(PAH - C_2H_2)^{0+} + {}^3C_2H_2^{2+}$	22.8	22.3
	$^1PAH^{2+} \rightarrow {}^3(PAH - C_2H_2)^{0+} + {}^3C_2H_2^{2+}$	21.3	21.0
	$^3PAH^{2+} \rightarrow {}^1(PAH - C_2H_2)^{2+} + {}^1C_2H_2^{0+}$	14.2	13.4
	$^3PAH^{2+} \rightarrow {}^3(PAH - C_2H_2)^{2+} + {}^1C_2H_2^{0+}$	12.4	11.6
	$^3PAH^{2+} \rightarrow {}^1(PAH - C_2H_2)^{2+} + {}^3C_2H_2^{0+}$	14.9	14.2
	$^3PAH^{2+} \rightarrow {}^3(PAH - C_2H_2)^{2+} + {}^3C_2H_2^{0+}$	13.1	12.4
	$\mathbf{^3PAH^{2+} \rightarrow {}^2(PAH - C_2H_2)^{1+} + {}^2C_2H_2^{1+}}$	**9.7**	**9.9**
	$^3PAH^{2+} \rightarrow {}^1(PAH - C_2H_2)^{0+} + {}^1C_2H_2^{2+}$	24.6	24.2
	$^3PAH^{2+} \rightarrow {}^3(PAH - C_2H_2)^{0+} + {}^1C_2H_2^{2+}$	23.1	22.9
	$^3PAH^{2+} \rightarrow {}^1(PAH - C_2H_2)^{0+} + {}^3C_2H_2^{2+}$	23.3	22.6
	$^3PAH^{2+} \rightarrow {}^3(PAH - C_2H_2)^{0+} + {}^3C_2H_2^{2+}$	21.8	21.3

Supplementary Table S6: Dissociation energies of acetylene loss from position #3 in PHE @ UKS-DFT/def2-TZVPP level of theory. All values were obtained without geometry relaxation. Bold text highlights the lowest energy dissociation path for a particular PAH charge state and spin state.

Position	Reaction	E_{diss}, eV	
		ωB97	M062x
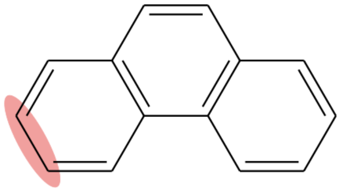	${}^{1}\mathrm{PAH}^{0+} \rightarrow {}^{1}(\mathrm{PAH} - \mathrm{C_2H_2})^{0+} + {}^{1}\mathrm{C_2H_2^{0+}}$	15.0	14.5
	**${}^{1}\mathrm{PAH}^{0+} \rightarrow {}^{3}(\mathrm{PAH} - \mathrm{C_2H_2})^{0+} + {}^{1}\mathrm{C_2H_2^{0+}}$**	**13.5**	**13.7**
	${}^{1}\mathrm{PAH}^{0+} \rightarrow {}^{1}(\mathrm{PAH} - \mathrm{C_2H_2})^{0+} + {}^{3}\mathrm{C_2H_2^{0+}}$	15.5	15.1
	${}^{1}\mathrm{PAH}^{0+} \rightarrow {}^{3}(\mathrm{PAH} - \mathrm{C_2H_2})^{0+} + {}^{3}\mathrm{C_2H_2^{0+}}$	13.9	14.2
	**${}^{2}\mathrm{PAH}^{1+} \rightarrow {}^{2}(\mathrm{PAH} - \mathrm{C_2H_2})^{1+} + {}^{1}\mathrm{C_2H_2^{0+}}$**	**12.7**	**14.0**
	${}^{2}\mathrm{PAH}^{1+} \rightarrow {}^{2}(\mathrm{PAH} - \mathrm{C_2H_2})^{1+} + {}^{3}\mathrm{C_2H_2^{0+}}$	13.1	14.5
	${}^{2}\mathrm{PAH}^{1+} \rightarrow {}^{1}(\mathrm{PAH} - \mathrm{C_2H_2})^{0+} + {}^{2}\mathrm{C_2H_2^{1+}}$	17.5	17.0
	${}^{2}\mathrm{PAH}^{1+} \rightarrow {}^{3}(\mathrm{PAH} - \mathrm{C_2H_2})^{0+} + {}^{2}\mathrm{C_2H_2^{1+}}$	16.0	16.2
	${}^{1}\mathrm{PAH}^{2+} \rightarrow {}^{1}(\mathrm{PAH} - \mathrm{C_2H_2})^{2+} + {}^{1}\mathrm{C_2H_2^{0+}}$	14.5	13.9
	${}^{1}\mathrm{PAH}^{2+} \rightarrow {}^{3}(\mathrm{PAH} - \mathrm{C_2H_2})^{2+} + {}^{1}\mathrm{C_2H_2^{0+}}$	12.7	12.1
	${}^{1}\mathrm{PAH}^{2+} \rightarrow {}^{1}(\mathrm{PAH} - \mathrm{C_2H_2})^{2+} + {}^{3}\mathrm{C_2H_2^{0+}}$	14.9	14.5
	${}^{1}\mathrm{PAH}^{2+} \rightarrow {}^{3}(\mathrm{PAH} - \mathrm{C_2H_2})^{2+} + {}^{3}\mathrm{C_2H_2^{0+}}$	13.1	12.6
	**${}^{1}\mathrm{PAH}^{2+} \rightarrow {}^{2}(\mathrm{PAH} - \mathrm{C_2H_2})^{1+} + {}^{2}\mathrm{C_2H_2^{1+}}$**	**10.1**	**11.7**
	${}^{1}\mathrm{PAH}^{2+} \rightarrow {}^{1}(\mathrm{PAH} - \mathrm{C_2H_2})^{0+} + {}^{1}\mathrm{C_2H_2^{2+}}$	25.3	25.1
	${}^{1}\mathrm{PAH}^{2+} \rightarrow {}^{3}(\mathrm{PAH} - \mathrm{C_2H_2})^{0+} + {}^{1}\mathrm{C_2H_2^{2+}}$	23.7	24.3
	${}^{1}\mathrm{PAH}^{2+} \rightarrow {}^{1}(\mathrm{PAH} - \mathrm{C_2H_2})^{0+} + {}^{3}\mathrm{C_2H_2^{2+}}$	24.0	23.5
	${}^{1}\mathrm{PAH}^{2+} \rightarrow {}^{3}(\mathrm{PAH} - \mathrm{C_2H_2})^{0+} + {}^{3}\mathrm{C_2H_2^{2+}}$	22.4	22.6
	${}^{3}\mathrm{PAH}^{2+} \rightarrow {}^{1}(\mathrm{PAH} - \mathrm{C_2H_2})^{2+} + {}^{1}\mathrm{C_2H_2^{0+}}$	15.0	14.2
	${}^{3}\mathrm{PAH}^{2+} \rightarrow {}^{3}(\mathrm{PAH} - \mathrm{C_2H_2})^{2+} + {}^{1}\mathrm{C_2H_2^{0+}}$	13.2	12.3
	${}^{3}\mathrm{PAH}^{2+} \rightarrow {}^{1}(\mathrm{PAH} - \mathrm{C_2H_2})^{2+} + {}^{3}\mathrm{C_2H_2^{0+}}$	15.5	14.8
	${}^{3}\mathrm{PAH}^{2+} \rightarrow {}^{3}(\mathrm{PAH} - \mathrm{C_2H_2})^{2+} + {}^{3}\mathrm{C_2H_2^{0+}}$	13.6	12.9
	**${}^{3}\mathrm{PAH}^{2+} \rightarrow {}^{2}(\mathrm{PAH} - \mathrm{C_2H_2})^{1+} + {}^{2}\mathrm{C_2H_2^{1+}}$**	**10.7**	**11.9**
	${}^{3}\mathrm{PAH}^{2+} \rightarrow {}^{1}(\mathrm{PAH} - \mathrm{C_2H_2})^{0+} + {}^{1}\mathrm{C_2H_2^{2+}}$	25.8	25.4
	${}^{3}\mathrm{PAH}^{2+} \rightarrow {}^{3}(\mathrm{PAH} - \mathrm{C_2H_2})^{0+} + {}^{1}\mathrm{C_2H_2^{2+}}$	24.2	24.6
	${}^{3}\mathrm{PAH}^{2+} \rightarrow {}^{1}(\mathrm{PAH} - \mathrm{C_2H_2})^{0+} + {}^{3}\mathrm{C_2H_2^{2+}}$	24.5	23.7
	${}^{3}\mathrm{PAH}^{2+} \rightarrow {}^{3}(\mathrm{PAH} - \mathrm{C_2H_2})^{0+} + {}^{3}\mathrm{C_2H_2^{2+}}$	22.9	22.9

Supplementary Table S7: Dissociation energies of acetylene loss from position #4 in PHE @ UKS-DFT/def2-TZVPP level of theory. All values were obtained without geometry relaxation. Bold text highlights the lowest energy dissociation path for a particular PAH charge state and spin state.

Position	Reaction	E_{diss}, eV	
		ωB97	M062x
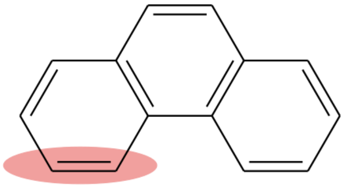	$^1PAH^{0+} \rightarrow {}^1(PAH - C_2H_2)^{0+} + {}^1C_2H_2^{0+}$	13.8	13.3
	$\mathbf{^1PAH^{0+} \rightarrow {}^3(PAH - C_2H_2)^{0+} + {}^1C_2H_2^{0+}}$	**12.1**	**11.9**
	$^1PAH^{0+} \rightarrow {}^1(PAH - C_2H_2)^{0+} + {}^3C_2H_2^{0+}$	14.4	14.0
	$^1PAH^{0+} \rightarrow {}^3(PAH - C_2H_2)^{0+} + {}^3C_2H_2^{0+}$	12.8	12.6
	$\mathbf{^2PAH^{1+} \rightarrow {}^2(PAH - C_2H_2)^{1+} + {}^1C_2H_2^{0+}}$	**11.8**	**12.0**
	$^2PAH^{1+} \rightarrow {}^2(PAH - C_2H_2)^{1+} + {}^3C_2H_2^{0+}$	12.4	12.7
	$^2PAH^{1+} \rightarrow {}^1(PAH - C_2H_2)^{0+} + {}^2C_2H_2^{1+}$	16.3	15.9
	$^2PAH^{1+} \rightarrow {}^3(PAH - C_2H_2)^{0+} + {}^2C_2H_2^{1+}$	14.7	14.5
	$^1PAH^{2+} \rightarrow {}^1(PAH - C_2H_2)^{2+} + {}^1C_2H_2^{0+}$	13.7	13.3
	$^1PAH^{2+} \rightarrow {}^3(PAH - C_2H_2)^{2+} + {}^1C_2H_2^{0+}$	12.0	11.8
	$^1PAH^{2+} \rightarrow {}^1(PAH - C_2H_2)^{2+} + {}^3C_2H_2^{0+}$	14.3	14.0
	$^1PAH^{2+} \rightarrow {}^3(PAH - C_2H_2)^{2+} + {}^3C_2H_2^{0+}$	12.7	12.5
	$\mathbf{^1PAH^{2+} \rightarrow {}^2(PAH - C_2H_2)^{1+} + {}^2C_2H_2^{1+}}$	**9.3**	**9.7**
	$^1PAH^{2+} \rightarrow {}^1(PAH - C_2H_2)^{0+} + {}^1C_2H_2^{2+}$	24.2	24.1
	$^1PAH^{2+} \rightarrow {}^3(PAH - C_2H_2)^{0+} + {}^1C_2H_2^{2+}$	22.6	22.7
	$^1PAH^{2+} \rightarrow {}^1(PAH - C_2H_2)^{0+} + {}^3C_2H_2^{2+}$	23.0	22.5
	$^1PAH^{2+} \rightarrow {}^3(PAH - C_2H_2)^{0+} + {}^3C_2H_2^{2+}$	21.3	21.1
	$^3PAH^{2+} \rightarrow {}^1(PAH - C_2H_2)^{2+} + {}^1C_2H_2^{0+}$	14.2	13.5
	$^3PAH^{2+} \rightarrow {}^3(PAH - C_2H_2)^{2+} + {}^1C_2H_2^{0+}$	12.6	12.0
	$^3PAH^{2+} \rightarrow {}^1(PAH - C_2H_2)^{2+} + {}^3C_2H_2^{0+}$	14.8	14.3
	$^3PAH^{2+} \rightarrow {}^3(PAH - C_2H_2)^{2+} + {}^3C_2H_2^{0+}$	13.2	12.8
	$\mathbf{^3PAH^{2+} \rightarrow {}^2(PAH - C_2H_2)^{1+} + {}^2C_2H_2^{1+}}$	**9.8**	**10.0**
	$^3PAH^{2+} \rightarrow {}^1(PAH - C_2H_2)^{0+} + {}^1C_2H_2^{2+}$	24.8	24.4
	$^3PAH^{2+} \rightarrow {}^3(PAH - C_2H_2)^{0+} + {}^1C_2H_2^{2+}$	23.1	23.0
	$^3PAH^{2+} \rightarrow {}^1(PAH - C_2H_2)^{0+} + {}^3C_2H_2^{2+}$	23.5	22.7
	$^3PAH^{2+} \rightarrow {}^3(PAH - C_2H_2)^{0+} + {}^3C_2H_2^{2+}$	21.9	21.4

Supplementary Table S8: Dissociation energies of acetylene loss from position #1 in PYR @ UKS-DFT/def2-TZVPP level of theory. All values were obtained without geometry relaxation. Bold text highlights the lowest energy dissociation path for a particular PAH charge state and spin state.

Position	Reaction	E_{diss}, eV	
		ωB97	M062x
	$^1PAH^{0+} \rightarrow {}^1(PAH - C_2H_4)^{0+} + {}^1C_2H_4^{0+}$	12.6	12.2
	**$^1PAH^{0+} \rightarrow {}^3(PAH - C_2H_4)^{0+} + {}^1C_2H_4^{0+}$**	**11.0**	**10.9**
	$^1PAH^{0+} \rightarrow {}^1(PAH - C_2H_4)^{0+} + {}^3C_2H_4^{0+}$	13.5	13.1
	$^1PAH^{0+} \rightarrow {}^3(PAH - C_2H_4)^{0+} + {}^3C_2H_4^{0+}$	12.0	11.9
	**$^2PAH^{1+} \rightarrow {}^2(PAH - C_2H_4)^{1+} + {}^1C_2H_4^{0+}$**	**12.8**	**12.4**
	$^2PAH^{1+} \rightarrow {}^2(PAH - C_2H_4)^{1+} + {}^3C_2H_4^{0+}$	13.7	13.3
	$^2PAH^{1+} \rightarrow {}^1(PAH - C_2H_4)^{0+} + {}^2C_2H_4^{1+}$	15.8	15.3
	$^2PAH^{1+} \rightarrow {}^3(PAH - C_2H_4)^{0+} + {}^2C_2H_4^{1+}$	14.2	14.1
	$^1PAH^{2+} \rightarrow {}^1(PAH - C_2H_4)^{2+} + {}^1C_2H_4^{0+}$	14.1	14.1
	$^1PAH^{2+} \rightarrow {}^3(PAH - C_2H_4)^{2+} + {}^1C_2H_4^{0+}$	13.0	12.8
	$^1PAH^{2+} \rightarrow {}^1(PAH - C_2H_4)^{2+} + {}^3C_2H_4^{0+}$	15.0	15.0
	$^1PAH^{2+} \rightarrow {}^3(PAH - C_2H_4)^{2+} + {}^3C_2H_4^{0+}$	13.9	13.8
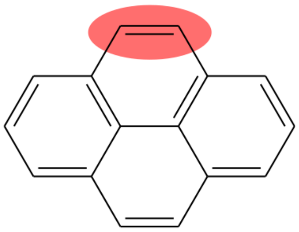	**$^1PAH^{2+} \rightarrow {}^2(PAH - C_2H_4)^{1+} + {}^2C_2H_4^{1+}$**	**11.1**	**10.9**
	$^1PAH^{2+} \rightarrow {}^1(PAH - C_2H_4)^{0+} + {}^1C_2H_4^{2+}$	24.5	24.3
	$^1PAH^{2+} \rightarrow {}^3(PAH - C_2H_4)^{0+} + {}^1C_2H_4^{2+}$	22.9	23.1
	$^1PAH^{2+} \rightarrow {}^1(PAH - C_2H_4)^{0+} + {}^3C_2H_4^{2+}$	23.2	22.8
	$^1PAH^{2+} \rightarrow {}^3(PAH - C_2H_4)^{0+} + {}^3C_2H_4^{2+}$	21.7	21.5
	$^3PAH^{2+} \rightarrow {}^1(PAH - C_2H_4)^{2+} + {}^1C_2H_4^{0+}$	13.6	13.5
	$^3PAH^{2+} \rightarrow {}^3(PAH - C_2H_4)^{2+} + {}^1C_2H_4^{0+}$	12.5	12.2
	$^3PAH^{2+} \rightarrow {}^1(PAH - C_2H_4)^{2+} + {}^3C_2H_4^{0+}$	14.5	14.5
	$^3PAH^{2+} \rightarrow {}^3(PAH - C_2H_4)^{2+} + {}^3C_2H_4^{0+}$	13.4	13.2
	**$^3PAH^{2+} \rightarrow {}^2(PAH - C_2H_4)^{1+} + {}^2C_2H_4^{1+}$**	**10.6**	**10.3**
	$^3PAH^{2+} \rightarrow {}^1(PAH - C_2H_4)^{0+} + {}^1C_2H_4^{2+}$	24.0	23.8
	$^3PAH^{2+} \rightarrow {}^3(PAH - C_2H_4)^{0+} + {}^1C_2H_4^{2+}$	22.4	22.5
	$^3PAH^{2+} \rightarrow {}^1(PAH - C_2H_4)^{0+} + {}^3C_2H_4^{2+}$	22.8	22.2
	$^3PAH^{2+} \rightarrow {}^3(PAH - C_2H_4)^{0+} + {}^3C_2H_4^{2+}$	21.2	20.9

Supplementary Table S9: Dissociation energies of acetylene loss from position #2 in PYR @ UKS-DFT/def2-TZVPP level of theory. All values were obtained without geometry relaxation. Bold text highlights the lowest energy dissociation path for a particular PAH charge state and spin state.

Position	Reaction	E_{diss}, eV	
		ωB97	M062x
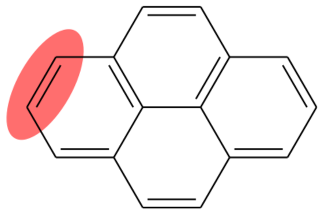	$^1PAH^{0+} \rightarrow {}^1(PAH - C_2H_4)^{0+} + {}^1C_2H_4^{0+}$	14.3	13.7
	$\mathbf{^1PAH^{0+} \rightarrow {}^3(PAH - C_2H_4)^{0+} + {}^1C_2H_4^{0+}}$	**13.1**	**13.1**
	$^1PAH^{0+} \rightarrow {}^1(PAH - C_2H_4)^{0+} + {}^3C_2H_4^{0+}$	14.8	14.4
	$^1PAH^{0+} \rightarrow {}^3(PAH - C_2H_4)^{0+} + {}^3C_2H_4^{0+}$	13.7	13.8
	$\mathbf{^2PAH^{1+} \rightarrow {}^2(PAH - C_2H_4)^{1+} + {}^1C_2H_4^{0+}}$	**12.1**	**11.7**
	$^2PAH^{1+} \rightarrow {}^2(PAH - C_2H_4)^{1+} + {}^3C_2H_4^{0+}$	12.6	12.4
	$^2PAH^{1+} \rightarrow {}^1(PAH - C_2H_4)^{0+} + {}^2C_2H_4^{1+}$	17.3	16.8
	$^2PAH^{1+} \rightarrow {}^3(PAH - C_2H_4)^{0+} + {}^2C_2H_4^{1+}$	16.2	16.2
	$^1PAH^{2+} \rightarrow {}^1(PAH - C_2H_4)^{2+} + {}^1C_2H_4^{0+}$	14.0	13.6
	$^1PAH^{2+} \rightarrow {}^3(PAH - C_2H_4)^{2+} + {}^1C_2H_4^{0+}$	13.7	13.5
	$^1PAH^{2+} \rightarrow {}^1(PAH - C_2H_4)^{2+} + {}^3C_2H_4^{0+}$	14.6	14.2
	$^1PAH^{2+} \rightarrow {}^3(PAH - C_2H_4)^{2+} + {}^3C_2H_4^{0+}$	14.3	14.2
	$\mathbf{^1PAH^{2+} \rightarrow {}^2(PAH - C_2H_4)^{1+} + {}^2C_2H_4^{1+}}$	**10.3**	**10.2**
	$^1PAH^{2+} \rightarrow {}^1(PAH - C_2H_4)^{0+} + {}^1C_2H_4^{2+}$	25.8	25.7
	$^1PAH^{2+} \rightarrow {}^3(PAH - C_2H_4)^{0+} + {}^1C_2H_4^{2+}$	24.7	25.1
	$^1PAH^{2+} \rightarrow {}^1(PAH - C_2H_4)^{0+} + {}^3C_2H_4^{2+}$	24.6	24.0
	$^1PAH^{2+} \rightarrow {}^3(PAH - C_2H_4)^{0+} + {}^3C_2H_4^{2+}$	23.4	23.4
	$^3PAH^{2+} \rightarrow {}^1(PAH - C_2H_4)^{2+} + {}^1C_2H_4^{0+}$	13.6	13.0
	$^3PAH^{2+} \rightarrow {}^3(PAH - C_2H_4)^{2+} + {}^1C_2H_4^{0+}$	13.3	12.9
	$^3PAH^{2+} \rightarrow {}^1(PAH - C_2H_4)^{2+} + {}^3C_2H_4^{0+}$	14.1	13.7
	$^3PAH^{2+} \rightarrow {}^3(PAH - C_2H_4)^{2+} + {}^3C_2H_4^{0+}$	13.8	13.6
	$\mathbf{^3PAH^{2+} \rightarrow {}^2(PAH - C_2H_4)^{1+} + {}^2C_2H_4^{1+}}$	**9.8**	**9.6**
	$^3PAH^{2+} \rightarrow {}^1(PAH - C_2H_4)^{0+} + {}^1C_2H_4^{2+}$	25.4	25.1
	$^3PAH^{2+} \rightarrow {}^3(PAH - C_2H_4)^{0+} + {}^1C_2H_4^{2+}$	24.2	24.5
	$^3PAH^{2+} \rightarrow {}^1(PAH - C_2H_4)^{0+} + {}^3C_2H_4^{2+}$	24.1	23.5
	$^3PAH^{2+} \rightarrow {}^3(PAH - C_2H_4)^{0+} + {}^3C_2H_4^{2+}$	22.9	22.8

Supplementary Table S10: Dissociation energies of $C_3H_x^+$ loss from position #1 in FLU @ UKS-DFT/def2-TZVPP level of theory. All values were obtained without geometry relaxation. Bold text highlights the lowest energy dissociation path for a particular PAH charge state and spin state.

Position	Reaction	E_{diss}, eV	
		ωB97	M062x
	**$^1PAH^{0+} \rightarrow {}^2(PAH - C_3H_3)^{0+} + {}^2C_3H_3^{0+}$**	**15.1**	**15.0**
	$^2PAH^{1+} \rightarrow {}^1(PAH - C_3H_3)^{1+} + {}^2C_3H_3^{0+}$	16.9	16.4
	**$^2PAH^{1+} \rightarrow {}^3(PAH - C_3H_3)^{1+} + {}^2C_3H_3^{0+}$**	**14.6**	**14.4**
	$^2PAH^{1+} \rightarrow {}^2(PAH - C_3H_3)^{0+} + {}^1C_3H_3^{1+}$	16.9	16.5
	$^2PAH^{1+} \rightarrow {}^2(PAH - C_3H_3)^{0+} + {}^3C_3H_3^{1+}$	14.7	14.6
	$^1PAH^{2+} \rightarrow {}^2(PAH - C_3H_3)^{2+} + {}^2C_3H_3^{0+}$	14.6	14.5
	$^1PAH^{2+} \rightarrow {}^1(PAH - C_3H_3)^{1+} + {}^1C_3H_3^{1+}$	13.5	12.8
	$^1PAH^{2+} \rightarrow {}^3(PAH - C_3H_3)^{1+} + {}^1C_3H_3^{1+}$	11.1	10.8
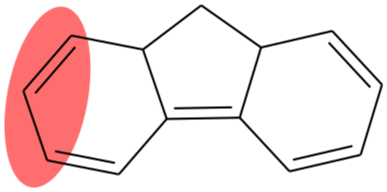	$^1PAH^{2+} \rightarrow {}^1(PAH - C_3H_3)^{1+} + {}^3C_3H_3^{1+}$	11.3	10.9
	**$^1PAH^{2+} \rightarrow {}^3(PAH - C_3H_3)^{1+} + {}^3C_3H_3^{1+}$**	**9.0**	**8.9**
	$^1PAH^{2+} \rightarrow {}^2(PAH - C_3H_3)^{0+} + {}^2C_3H_3^{2+}$	19.7	19.5
	$^3PAH^{2+} \rightarrow {}^2(PAH - C_3H_3)^{2+} + {}^2C_3H_3^{0+}$	14.8	14.4
	$^3PAH^{2+} \rightarrow {}^1(PAH - C_3H_3)^{1+} + {}^1C_3H_3^{1+}$	13.7	12.7
	$^3PAH^{2+} \rightarrow {}^3(PAH - C_3H_3)^{1+} + {}^1C_3H_3^{1+}$	11.3	10.7
	$^3PAH^{2+} \rightarrow {}^1(PAH - C_3H_3)^{1+} + {}^3C_3H_3^{1+}$	11.5	10.8
	**$^3PAH^{2+} \rightarrow {}^3(PAH - C_3H_3)^{1+} + {}^3C_3H_3^{1+}$**	**9.1**	**8.8**
	$^3PAH^{2+} \rightarrow {}^2(PAH - C_3H_3)^{0+} + {}^2C_3H_3^{2+}$	19.8	19.4

Supplementary Table S11: Dissociation energies of $C_3H_x^+$ loss from position #2 in FLU @ UKS-DFT/def2-TZVPP level of theory. All values were obtained without geometry relaxation. Bold text highlights the lowest energy dissociation path for a particular PAH charge state and spin state.

Position	Reaction	E_{diss}, eV	
		ωB97	M062x
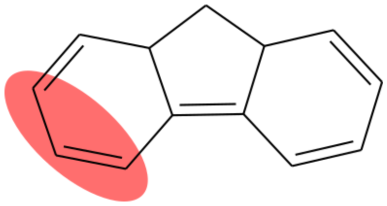	**$^1\mathrm{PAH}^{0+} \rightarrow {}^2(\mathrm{PAH}-\mathrm{C_3H_3})^{0+} + {}^2\mathrm{C_3H_3^{0+}}$**	**14.8**	**14.8**
	$^2\mathrm{PAH}^{1+} \rightarrow {}^1(\mathrm{PAH}-\mathrm{C_3H_3})^{1+} + {}^2\mathrm{C_3H_3^{0+}}$	16.3	15.7
	**$^2\mathrm{PAH}^{1+} \rightarrow {}^3(\mathrm{PAH}-\mathrm{C_3H_3})^{1+} + {}^2\mathrm{C_3H_3^{0+}}$**	**13.9**	**13.6**
	$^2\mathrm{PAH}^{1+} \rightarrow {}^2(\mathrm{PAH}-\mathrm{C_3H_3})^{0+} + {}^1\mathrm{C_3H_3^{1+}}$	16.7	16.3
	$^2\mathrm{PAH}^{1+} \rightarrow {}^2(\mathrm{PAH}-\mathrm{C_3H_3})^{0+} + {}^3\mathrm{C_3H_3^{1+}}$	14.5	14.4
	$^1\mathrm{PAH}^{2+} \rightarrow {}^2(\mathrm{PAH}-\mathrm{C_3H_3})^{2+} + {}^2\mathrm{C_3H_3^{0+}}$	15.7	15.5
	$^1\mathrm{PAH}^{2+} \rightarrow {}^1(\mathrm{PAH}-\mathrm{C_3H_3})^{1+} + {}^1\mathrm{C_3H_3^{1+}}$	12.9	12.1
	$^1\mathrm{PAH}^{2+} \rightarrow {}^3(\mathrm{PAH}-\mathrm{C_3H_3})^{1+} + {}^1\mathrm{C_3H_3^{1+}}$	10.5	10.1
	$^1\mathrm{PAH}^{2+} \rightarrow {}^1(\mathrm{PAH}-\mathrm{C_3H_3})^{1+} + {}^3\mathrm{C_3H_3^{1+}}$	10.7	10.3
	**$^1\mathrm{PAH}^{2+} \rightarrow {}^3(\mathrm{PAH}-\mathrm{C_3H_3})^{1+} + {}^3\mathrm{C_3H_3^{1+}}$**	**8.3**	**8.2**
	$^1\mathrm{PAH}^{2+} \rightarrow {}^2(\mathrm{PAH}-\mathrm{C_3H_3})^{0+} + {}^2\mathrm{C_3H_3^{2+}}$	19.4	19.3
	$^3\mathrm{PAH}^{2+} \rightarrow {}^2(\mathrm{PAH}-\mathrm{C_3H_3})^{2+} + {}^2\mathrm{C_3H_3^{0+}}$	15.8	15.4
	$^3\mathrm{PAH}^{2+} \rightarrow {}^1(\mathrm{PAH}-\mathrm{C_3H_3})^{1+} + {}^1\mathrm{C_3H_3^{1+}}$	13.0	12.0
	$^3\mathrm{PAH}^{2+} \rightarrow {}^3(\mathrm{PAH}-\mathrm{C_3H_3})^{1+} + {}^1\mathrm{C_3H_3^{1+}}$	10.6	9.9
	$^3\mathrm{PAH}^{2+} \rightarrow {}^1(\mathrm{PAH}-\mathrm{C_3H_3})^{1+} + {}^3\mathrm{C_3H_3^{1+}}$	10.9	10.2
	**$^3\mathrm{PAH}^{2+} \rightarrow {}^3(\mathrm{PAH}-\mathrm{C_3H_3})^{1+} + {}^3\mathrm{C_3H_3^{1+}}$**	**8.5**	**8.1**
	$^3\mathrm{PAH}^{2+} \rightarrow {}^2(\mathrm{PAH}-\mathrm{C_3H_3})^{0+} + {}^2\mathrm{C_3H_3^{2+}}$	19.6	19.2

Supplementary Table S12: Dissociation energies of $C_3H_x^+$ loss from position #1 in PHE @ UKS-DFT/def2-TZVPP level of theory. All values were obtained without geometry relaxation. Bold text highlights the lowest energy dissociation path for a particular PAH charge state and spin state.

Position	Reaction	E_{diss}, eV	
		ωB97	M062x
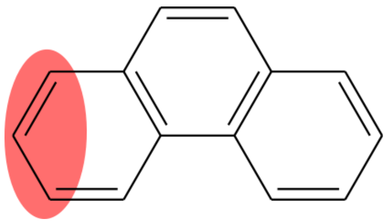	$\mathbf{^1PAH^{0+} \rightarrow {}^2(PAH - C_3H_3)^{0+} + {}^2C_3H_3^{0+}}$	**14.5**	**14.8**
	$^2PAH^{1+} \rightarrow {}^1(PAH - C_3H_3)^{1+} + {}^2C_3H_3^{0+}$	16.4	16.4
	$\mathbf{^2PAH^{1+} \rightarrow {}^3(PAH - C_3H_3)^{1+} + {}^2C_3H_3^{0+}}$	**13.6**	**13.7**
	$^2PAH^{1+} \rightarrow {}^2(PAH - C_3H_3)^{0+} + {}^1C_3H_3^{1+}$	16.8	16.4
	$^2PAH^{1+} \rightarrow {}^2(PAH - C_3H_3)^{0+} + {}^3C_3H_3^{1+}$	14.6	14.5
	$^1PAH^{2+} \rightarrow {}^2(PAH - C_3H_3)^{2+} + {}^2C_3H_3^{0+}$	13.9	14.4
	$^1PAH^{2+} \rightarrow {}^1(PAH - C_3H_3)^{1+} + {}^1C_3H_3^{1+}$	13.7	13.1
	$^1PAH^{2+} \rightarrow {}^3(PAH - C_3H_3)^{1+} + {}^1C_3H_3^{1+}$	10.8	10.5
	$^1PAH^{2+} \rightarrow {}^1(PAH - C_3H_3)^{1+} + {}^3C_3H_3^{1+}$	11.5	11.2
	$\mathbf{^1PAH^{2+} \rightarrow {}^3(PAH - C_3H_3)^{1+} + {}^3C_3H_3^{1+}}$	**8.6**	**8.5**
	$^1PAH^{2+} \rightarrow {}^2(PAH - C_3H_3)^{0+} + {}^2C_3H_3^{2+}$	19.6	19.6
	$^3PAH^{2+} \rightarrow {}^2(PAH - C_3H_3)^{2+} + {}^2C_3H_3^{0+}$	14.4	14.6
	$^3PAH^{2+} \rightarrow {}^1(PAH - C_3H_3)^{1+} + {}^1C_3H_3^{1+}$	14.2	13.4
	$^3PAH^{2+} \rightarrow {}^3(PAH - C_3H_3)^{1+} + {}^1C_3H_3^{1+}$	11.3	10.7
	$^3PAH^{2+} \rightarrow {}^1(PAH - C_3H_3)^{1+} + {}^3C_3H_3^{1+}$	12.0	11.5
	$\mathbf{^3PAH^{2+} \rightarrow {}^3(PAH - C_3H_3)^{1+} + {}^3C_3H_3^{1+}}$	**9.1**	**8.8**
	$^3PAH^{2+} \rightarrow {}^2(PAH - C_3H_3)^{0+} + {}^2C_3H_3^{2+}$	20.1	19.9

Supplementary Table S13: Dissociation energies of $C_3H_x^+$ loss from position #2 in PHE @ UKS-DFT/def2-TZVPP level of theory. All values were obtained without geometry relaxation. Bold text highlights the lowest energy dissociation path for a particular PAH charge state and spin state.

Position	Reaction	E_{diss}, eV	
		ωB97	M062x
	**${}^1\mathrm{PAH}^{0+} \rightarrow {}^2(\mathrm{PAH}-\mathrm{C_3H_3})^{0+} + {}^2\mathrm{C_3H_3^{0+}}$**	**15.5**	**14.9**
	${}^2\mathrm{PAH}^{1+} \rightarrow {}^1(\mathrm{PAH}-\mathrm{C_3H_3})^{1+} + {}^2\mathrm{C_3H_3^{0+}}$	16.4	15.8
	**${}^2\mathrm{PAH}^{1+} \rightarrow {}^3(\mathrm{PAH}-\mathrm{C_3H_3})^{1+} + {}^2\mathrm{C_3H_3^{0+}}$**	**14.0**	**13.8**
	${}^2\mathrm{PAH}^{1+} \rightarrow {}^2(\mathrm{PAH}-\mathrm{C_3H_3})^{0+} + {}^1\mathrm{C_3H_3^{1+}}$	17.4	16.4
	${}^2\mathrm{PAH}^{1+} \rightarrow {}^2(\mathrm{PAH}-\mathrm{C_3H_3})^{0+} + {}^3\mathrm{C_3H_3^{1+}}$	15.2	14.6
	${}^1\mathrm{PAH}^{2+} \rightarrow {}^2(\mathrm{PAH}-\mathrm{C_3H_3})^{2+} + {}^2\mathrm{C_3H_3^{0+}}$	14.0	14.1
	${}^1\mathrm{PAH}^{2+} \rightarrow {}^1(\mathrm{PAH}-\mathrm{C_3H_3})^{1+} + {}^1\mathrm{C_3H_3^{1+}}$	13.2	12.5
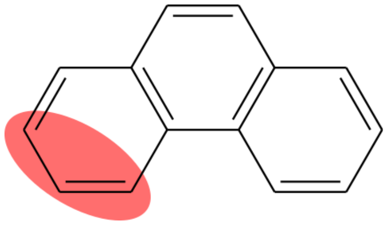	${}^1\mathrm{PAH}^{2+} \rightarrow {}^3(\mathrm{PAH}-\mathrm{C_3H_3})^{1+} + {}^1\mathrm{C_3H_3^{1+}}$	10.9	10.5
	${}^1\mathrm{PAH}^{2+} \rightarrow {}^1(\mathrm{PAH}-\mathrm{C_3H_3})^{1+} + {}^3\mathrm{C_3H_3^{1+}}$	11.0	10.7
	**${}^1\mathrm{PAH}^{2+} \rightarrow {}^3(\mathrm{PAH}-\mathrm{C_3H_3})^{1+} + {}^3\mathrm{C_3H_3^{1+}}$**	**8.7**	**8.6**
	${}^1\mathrm{PAH}^{2+} \rightarrow {}^2(\mathrm{PAH}-\mathrm{C_3H_3})^{0+} + {}^2\mathrm{C_3H_3^{2+}}$	20.4	19.8
	${}^3\mathrm{PAH}^{2+} \rightarrow {}^2(\mathrm{PAH}-\mathrm{C_3H_3})^{2+} + {}^2\mathrm{C_3H_3^{0+}}$	14.5	14.3
	${}^3\mathrm{PAH}^{2+} \rightarrow {}^1(\mathrm{PAH}-\mathrm{C_3H_3})^{1+} + {}^1\mathrm{C_3H_3^{1+}}$	13.7	12.8
	${}^3\mathrm{PAH}^{2+} \rightarrow {}^3(\mathrm{PAH}-\mathrm{C_3H_3})^{1+} + {}^1\mathrm{C_3H_3^{1+}}$	11.4	10.8
	${}^3\mathrm{PAH}^{2+} \rightarrow {}^1(\mathrm{PAH}-\mathrm{C_3H_3})^{1+} + {}^3\mathrm{C_3H_3^{1+}}$	11.5	10.9
	**${}^3\mathrm{PAH}^{2+} \rightarrow {}^3(\mathrm{PAH}-\mathrm{C_3H_3})^{1+} + {}^3\mathrm{C_3H_3^{1+}}$**	**9.2**	**8.9**
	${}^3\mathrm{PAH}^{2+} \rightarrow {}^2(\mathrm{PAH}-\mathrm{C_3H_3})^{0+} + {}^2\mathrm{C_3H_3^{2+}}$	20.9	20.0

Supplementary Table S14: Dissociation energies of $C_3H_x^+$ loss from PYR @ UKS-DFT/def2-TZVPP level of theory. All values were obtained without geometry relaxation. Bold text highlights the lowest energy dissociation path for a particular PAH charge state and spin state.

Position	Reaction	E_{diss}, eV	
		ωB97	M062x
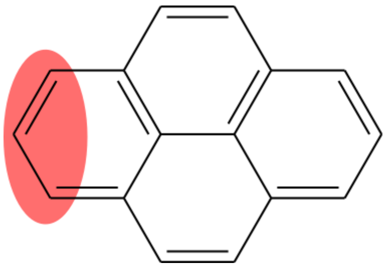	**$^{1}PAH^{0+} \rightarrow {}^{2}(PAH - C_3H_3)^{0+} + {}^{2}C_3H_3^{0+}$**	**15.2**	**14.9**
	$^{2}PAH^{1+} \rightarrow {}^{1}(PAH - C_3H_3)^{1+} + {}^{2}C_3H_3^{0+}$	16.0	15.4
	**$^{2}PAH^{1+} \rightarrow {}^{3}(PAH - C_3H_3)^{1+} + {}^{2}C_3H_3^{0+}$**	**13.6**	**13.4**
	$^{2}PAH^{1+} \rightarrow {}^{2}(PAH - C_3H_3)^{0+} + {}^{1}C_3H_3^{1+}$	17.5	17.0
	$^{2}PAH^{1+} \rightarrow {}^{2}(PAH - C_3H_3)^{0+} + {}^{3}C_3H_3^{1+}$	15.3	15.1
	$^{1}PAH^{2+} \rightarrow {}^{2}(PAH - C_3H_3)^{2+} + {}^{2}C_3H_3^{0+}$	15.8	15.5
	$^{1}PAH^{2+} \rightarrow {}^{1}(PAH - C_3H_3)^{1+} + {}^{1}C_3H_3^{1+}$	13.5	12.8
	$^{1}PAH^{2+} \rightarrow {}^{3}(PAH - C_3H_3)^{1+} + {}^{1}C_3H_3^{1+}$	11.1	10.8
	$^{1}PAH^{2+} \rightarrow {}^{1}(PAH - C_3H_3)^{1+} + {}^{3}C_3H_3^{1+}$	11.3	10.9
	**$^{1}PAH^{2+} \rightarrow {}^{3}(PAH - C_3H_3)^{1+} + {}^{3}C_3H_3^{1+}$**	**8.9**	**8.9**
	$^{1}PAH^{2+} \rightarrow {}^{2}(PAH - C_3H_3)^{0+} + {}^{2}C_3H_3^{2+}$	21.2	21.0
	$^{3}PAH^{2+} \rightarrow {}^{2}(PAH - C_3H_3)^{2+} + {}^{2}C_3H_3^{0+}$	15.3	14.9
	$^{3}PAH^{2+} \rightarrow {}^{1}(PAH - C_3H_3)^{1+} + {}^{1}C_3H_3^{1+}$	13.0	12.2
	$^{3}PAH^{2+} \rightarrow {}^{3}(PAH - C_3H_3)^{1+} + {}^{1}C_3H_3^{1+}$	10.6	10.2
	$^{3}PAH^{2+} \rightarrow {}^{1}(PAH - C_3H_3)^{1+} + {}^{3}C_3H_3^{1+}$	10.8	10.3
	**$^{3}PAH^{2+} \rightarrow {}^{3}(PAH - C_3H_3)^{1+} + {}^{3}C_3H_3^{1+}$**	**8.5**	**8.3**
	$^{3}PAH^{2+} \rightarrow {}^{2}(PAH - C_3H_3)^{0+} + {}^{2}C_3H_3^{2+}$	20.7	20.4

Supplementary Table S15: Dissociation energies of $C_4H_x^+$ loss from FLY @ UKS-DFT/def2-TZVPP level of theory. All values were obtained without geometry relaxation. Bold text highlights the lowest energy dissociation path for a particular PAH charge state and spin state.

Position	Reaction	E_{diss}, eV	
		ωB97	M062x
	${}^1PAH^{0+} \rightarrow {}^1(PAH - C_4H_4)^{0+} + {}^1C_4H_4^{0+}$	15.4	14.8
	${}^1PAH^{0+} \rightarrow {}^3(PAH - C_4H_4)^{0+} + {}^1C_4H_4^{0+}$	15.3	15.1
	$\mathbf{{}^1PAH^{0+} \rightarrow {}^1(PAH - C_4H_4)^{0+} + {}^3C_4H_4^{0+}}$	**13.7**	**13.5**
	$\mathbf{{}^1PAH^{0+} \rightarrow {}^3(PAH - C_4H_4)^{0+} + {}^3C_4H_4^{0+}}$	**13.7**	13.7
	${}^2PAH^{1+} \rightarrow {}^2(PAH - C_4H_4)^{1+} + {}^1C_4H_4^{0+}$	14.8	15.2
	$\mathbf{{}^2PAH^{1+} \rightarrow {}^2(PAH - C_4H_4)^{1+} + {}^3C_4H_4^{0+}}$	**13.2**	**13.8**
	${}^2PAH^{1+} \rightarrow {}^1(PAH - C_4H_4)^{0+} + {}^2C_4H_4^{1+}$	15.9	15.3
	${}^2PAH^{1+} \rightarrow {}^3(PAH - C_4H_4)^{0+} + {}^2C_4H_4^{1+}$	15.8	15.6
	${}^1PAH^{2+} \rightarrow {}^1(PAH - C_4H_4)^{2+} + {}^1C_4H_4^{0+}$	17.0	16.5
	${}^1PAH^{2+} \rightarrow {}^3(PAH - C_4H_4)^{2+} + {}^1C_4H_4^{0+}$	16.8	16.5
	${}^1PAH^{2+} \rightarrow {}^1(PAH - C_4H_4)^{2+} + {}^3C_4H_4^{0+}$	15.4	15.2
	${}^1PAH^{2+} \rightarrow {}^3(PAH - C_4H_4)^{2+} + {}^3C_4H_4^{0+}$	15.2	15.1
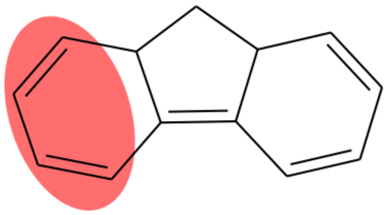	$\mathbf{{}^1PAH^{2+} \rightarrow {}^2(PAH - C_4H_4)^{1+} + {}^2C_4H_4^{1+}}$	**10.0**	**10.6**
	${}^1PAH^{2+} \rightarrow {}^1(PAH - C_4H_4)^{0+} + {}^1C_4H_4^{2+}$	18.8	18.6
	${}^1PAH^{2+} \rightarrow {}^3(PAH - C_4H_4)^{0+} + {}^1C_4H_4^{2+}$	18.7	18.9
	${}^1PAH^{2+} \rightarrow {}^1(PAH - C_4H_4)^{0+} + {}^3C_4H_4^{2+}$	18.0	17.6
	${}^1PAH^{2+} \rightarrow {}^3(PAH - C_4H_4)^{0+} + {}^3C_4H_4^{2+}$	17.9	17.8
	${}^3PAH^{2+} \rightarrow {}^1(PAH - C_4H_4)^{2+} + {}^1C_4H_4^{0+}$	17.2	16.4
	${}^3PAH^{2+} \rightarrow {}^3(PAH - C_4H_4)^{2+} + {}^1C_4H_4^{0+}$	17.0	16.4
	${}^3PAH^{2+} \rightarrow {}^1(PAH - C_4H_4)^{2+} + {}^3C_4H_4^{0+}$	15.6	15.1
	${}^3PAH^{2+} \rightarrow {}^3(PAH - C_4H_4)^{2+} + {}^3C_4H_4^{0+}$	15.4	15.0
	$\mathbf{{}^3PAH^{2+} \rightarrow {}^2(PAH - C_4H_4)^{1+} + {}^2C_4H_4^{1+}}$	**10.2**	**10.5**
	${}^3PAH^{2+} \rightarrow {}^1(PAH - C_4H_4)^{0+} + {}^1C_4H_4^{2+}$	18.9	18.5
	${}^3PAH^{2+} \rightarrow {}^3(PAH - C_4H_4)^{0+} + {}^1C_4H_4^{2+}$	18.9	18.8
	${}^3PAH^{2+} \rightarrow {}^1(PAH - C_4H_4)^{0+} + {}^3C_4H_4^{2+}$	18.1	17.5
	${}^3PAH^{2+} \rightarrow {}^3(PAH - C_4H_4)^{0+} + {}^3C_4H_4^{2+}$	18.1	17.7

Supplementary Table S16: Dissociation energies of $C_4H_x^+$ loss from PHE @ UKS-DFT/def2-TZVPP level of theory. All values were obtained without geometry relaxation. Bold text highlights the lowest energy dissociation path for a particular PAH charge state and spin state.

Position	Reaction	E_{diss}, eV	
		ωB97	M062x
	$^1PAH^{0+} \rightarrow {}^1(PAH - C_4H_4)^{0+} + {}^1C_4H_4^{0+}$	14.2	13.7
	$^1PAH^{0+} \rightarrow {}^3(PAH - C_4H_4)^{0+} + {}^1C_4H_4^{0+}$	14.3	14.8
	$\mathbf{^1PAH^{0+} \rightarrow {}^1(PAH - C_4H_4)^{0+} + {}^3C_4H_4^{0+}}$	**12.7**	**12.4**
	$\mathbf{^1PAH^{0+} \rightarrow {}^3(PAH - C_4H_4)^{0+} + {}^3C_4H_4^{0+}}$	**12.7**	13.5
	$^2PAH^{1+} \rightarrow {}^2(PAH - C_4H_4)^{1+} + {}^1C_4H_4^{0+}$	14.6	14.1
	$\mathbf{^2PAH^{1+} \rightarrow {}^2(PAH - C_4H_4)^{1+} + {}^3C_4H_4^{0+}}$	**13.1**	**12.8**
	$^2PAH^{1+} \rightarrow {}^1(PAH - C_4H_4)^{0+} + {}^2C_4H_4^{1+}$	14.8	14.3
	$^2PAH^{1+} \rightarrow {}^3(PAH - C_4H_4)^{0+} + {}^2C_4H_4^{1+}$	14.8	15.3
	$^1PAH^{2+} \rightarrow {}^1(PAH - C_4H_4)^{2+} + {}^1C_4H_4^{0+}$	15.8	15.4
	$^1PAH^{2+} \rightarrow {}^3(PAH - C_4H_4)^{2+} + {}^1C_4H_4^{0+}$	15.5	15.3
	$^1PAH^{2+} \rightarrow {}^1(PAH - C_4H_4)^{2+} + {}^3C_4H_4^{0+}$	14.2	14.1
	$^1PAH^{2+} \rightarrow {}^3(PAH - C_4H_4)^{2+} + {}^3C_4H_4^{0+}$	14.0	14.0
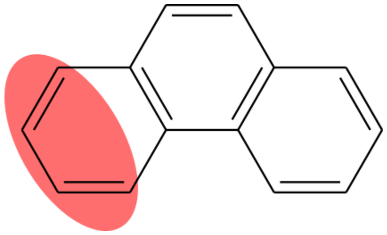	$\mathbf{^1PAH^{2+} \rightarrow {}^2(PAH - C_4H_4)^{1+} + {}^2C_4H_4^{1+}}$	**10.2**	**9.9**
	$^1PAH^{2+} \rightarrow {}^1(PAH - C_4H_4)^{0+} + {}^1C_4H_4^{2+}$	17.9	17.9
	$^1PAH^{2+} \rightarrow {}^3(PAH - C_4H_4)^{0+} + {}^1C_4H_4^{2+}$	18.0	19.0
	$^1PAH^{2+} \rightarrow {}^1(PAH - C_4H_4)^{0+} + {}^3C_4H_4^{2+}$	17.0	16.9
	$^1PAH^{2+} \rightarrow {}^3(PAH - C_4H_4)^{0+} + {}^3C_4H_4^{2+}$	17.1	18.0
	$^3PAH^{2+} \rightarrow {}^1(PAH - C_4H_4)^{2+} + {}^1C_4H_4^{0+}$	16.3	15.6
	$^3PAH^{2+} \rightarrow {}^3(PAH - C_4H_4)^{2+} + {}^1C_4H_4^{0+}$	16.1	15.6
	$^3PAH^{2+} \rightarrow {}^1(PAH - C_4H_4)^{2+} + {}^3C_4H_4^{0+}$	14.8	14.3
	$^3PAH^{2+} \rightarrow {}^3(PAH - C_4H_4)^{2+} + {}^3C_4H_4^{0+}$	14.5	14.3
	$\mathbf{^3PAH^{2+} \rightarrow {}^2(PAH - C_4H_4)^{1+} + {}^2C_4H_4^{1+}}$	**10.7**	**10.2**
	$^3PAH^{2+} \rightarrow {}^1(PAH - C_4H_4)^{0+} + {}^1C_4H_4^{2+}$	18.5	18.2
	$^3PAH^{2+} \rightarrow {}^3(PAH - C_4H_4)^{0+} + {}^1C_4H_4^{2+}$	18.5	19.2
	$^3PAH^{2+} \rightarrow {}^1(PAH - C_4H_4)^{0+} + {}^3C_4H_4^{2+}$	17.5	17.2
	$^3PAH^{2+} \rightarrow {}^3(PAH - C_4H_4)^{0+} + {}^3C_4H_4^{2+}$	17.6	18.3

Supplementary Table S17: Dissociation energies of proton or hydrogen atom loss from position #1 in FLU @ UKS-DFT/def2-TZVPP level of theory. All values were obtained without geometry relaxation. Bold text highlights the lowest energy dissociation path for a particular PAH charge state and spin state.

Position	Reaction	E_{diss}, eV	
		ωB97	M062x
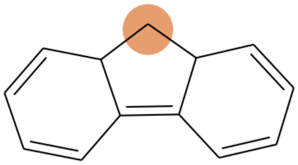	$\mathbf{{}^{1}PAH^{0+} \rightarrow {}^{2}(PAH - H)^{0+} + {}^{2}H^{0+}}$	**4.6**	**4.6**
	$\mathbf{{}^{2}PAH^{1+} \rightarrow {}^{1}(PAH - H)^{1+} + {}^{2}H^{0+}}$	**4.6**	**4.4**
	${}^{2}\mathrm{PAH}^{1+} \rightarrow {}^{3}(\mathrm{PAH} - \mathrm{H})^{1+} + {}^{2}\mathrm{H}^{0+}$	4.8	4.7
	${}^{2}\mathrm{PAH}^{1+} \rightarrow {}^{2}(\mathrm{PAH} - \mathrm{H})^{0+} + {}^{1}\mathrm{H}^{1+}$	10.3	10.1
	$\mathbf{{}^{1}PAH^{2+} \rightarrow {}^{2}(PAH - H)^{2+} + {}^{2}H^{0+}}$	**4.0**	**3.9**
	${}^{1}\mathrm{PAH}^{2+} \rightarrow {}^{1}(\mathrm{PAH} - \mathrm{H})^{1+} + {}^{1}\mathrm{H}^{1+}$	5.0	4.8
	${}^{1}\mathrm{PAH}^{2+} \rightarrow {}^{3}(\mathrm{PAH} - \mathrm{H})^{1+} + {}^{1}\mathrm{H}^{1+}$	5.2	5.1
	$\mathbf{{}^{3}PAH^{2+} \rightarrow {}^{2}(PAH - H)^{2+} + {}^{2}H^{0+}}$	**4.1**	**3.8**
	${}^{3}\mathrm{PAH}^{2+} \rightarrow {}^{1}(\mathrm{PAH} - \mathrm{H})^{1+} + {}^{1}\mathrm{H}^{1+}$	5.2	4.7
	${}^{3}\mathrm{PAH}^{2+} \rightarrow {}^{3}(\mathrm{PAH} - \mathrm{H})^{1+} + {}^{1}\mathrm{H}^{1+}$	5.3	5.0

Supplementary Table S18: Dissociation energies of proton or hydrogen atom loss from position #2 in FLU @ UKS-DFT/def2-TZVPP level of theory. All values were obtained without geometry relaxation. Bold text highlights the lowest energy dissociation path for a particular PAH charge state and spin state.

Position	Reaction	E_{diss}, eV	
		ωB97	M062x
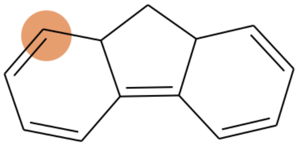	$\mathbf{{}^{1}PAH^{0+} \rightarrow {}^{2}(PAH - H)^{0+} + {}^{2}H^{0+}}$	**5.2**	**5.2**
	${}^{2}\mathrm{PAH}^{1+} \rightarrow {}^{1}(\mathrm{PAH} - \mathrm{H})^{1+} + {}^{2}\mathrm{H}^{0+}$	6.5	6.3
	$\mathbf{{}^{2}PAH^{1+} \rightarrow {}^{3}(PAH - H)^{1+} + {}^{2}H^{0+}}$	**5.4**	**5.3**
	${}^{2}\mathrm{PAH}^{1+} \rightarrow {}^{2}(\mathrm{PAH} - \mathrm{H})^{0+} + {}^{1}\mathrm{H}^{1+}$	10.9	10.6
	$\mathbf{{}^{1}PAH^{2+} \rightarrow {}^{2}(PAH - H)^{2+} + {}^{2}H^{0+}}$	**5.5**	**5.6**
	${}^{1}\mathrm{PAH}^{2+} \rightarrow {}^{1}(\mathrm{PAH} - \mathrm{H})^{1+} + {}^{1}\mathrm{H}^{1+}$	6.9	6.7
	${}^{1}\mathrm{PAH}^{2+} \rightarrow {}^{3}(\mathrm{PAH} - \mathrm{H})^{1+} + {}^{1}\mathrm{H}^{1+}$	5.8	5.7
	$\mathbf{{}^{3}PAH^{2+} \rightarrow {}^{2}(PAH - H)^{2+} + {}^{2}H^{0+}}$	**5.7**	**5.5**
	${}^{3}\mathrm{PAH}^{2+} \rightarrow {}^{1}(\mathrm{PAH} - \mathrm{H})^{1+} + {}^{1}\mathrm{H}^{1+}$	7.1	6.6
	${}^{3}\mathrm{PAH}^{2+} \rightarrow {}^{3}(\mathrm{PAH} - \mathrm{H})^{1+} + {}^{1}\mathrm{H}^{1+}$	5.9	5.6

Supplementary Table S19: Dissociation energies of proton or hydrogen atom loss from position #3 in FLU @ UKS-DFT/def2-TZVPP level of theory. All values were obtained without geometry relaxation. Bold text highlights the lowest energy dissociation path for a particular PAH charge state and spin state.

Position	Reaction	E_{diss}, eV	
		ωB97	M062x
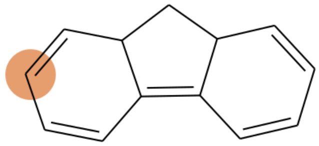	**$^{1}PAH^{0+} \rightarrow {}^{2}(PAH - H)^{0+} + {}^{2}H^{0+}$**	**5.2**	**5.2**
	$^{2}PAH^{1+} \rightarrow {}^{1}(PAH - H)^{1+} + {}^{2}H^{0+}$	6.5	6.4
	**$^{2}PAH^{1+} \rightarrow {}^{3}(PAH - H)^{1+} + {}^{2}H^{0+}$**	**5.2**	**5.2**
	$^{2}PAH^{1+} \rightarrow {}^{2}(PAH - H)^{0+} + {}^{1}H^{1+}$	10.9	10.6
	**$^{1}PAH^{2+} \rightarrow {}^{2}(PAH - H)^{2+} + {}^{2}H^{0+}$**	**5.5**	5.6
	$^{1}PAH^{2+} \rightarrow {}^{1}(PAH - H)^{1+} + {}^{1}H^{1+}$	6.9	6.7
	**$^{1}PAH^{2+} \rightarrow {}^{3}(PAH - H)^{1+} + {}^{1}H^{1+}$**	5.6	**5.5**
	**$^{3}PAH^{2+} \rightarrow {}^{2}(PAH - H)^{2+} + {}^{2}H^{0+}$**	**5.7**	5.5
	$^{3}PAH^{2+} \rightarrow {}^{1}(PAH - H)^{1+} + {}^{1}H^{1+}$	7.1	6.6
	**$^{3}PAH^{2+} \rightarrow {}^{3}(PAH - H)^{1+} + {}^{1}H^{1+}$**	5.8	**5.4**

Supplementary Table S20: Dissociation energies of proton or hydrogen atom loss from position #4 in FLU @ UKS-DFT/def2-TZVPP level of theory. All values were obtained without geometry relaxation. Bold text highlights the lowest energy dissociation path for a particular PAH charge state and spin state.

Position	Reaction	E_{diss}, eV	
		ωB97	M062x
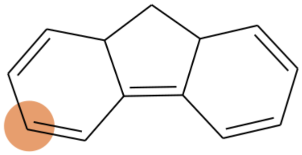	**$^{1}PAH^{0+} \rightarrow {}^{2}(PAH - H)^{0+} + {}^{2}H^{0+}$**	**5.2**	**5.2**
	$^{2}PAH^{1+} \rightarrow {}^{1}(PAH - H)^{1+} + {}^{2}H^{0+}$	6.5	6.4
	**$^{2}PAH^{1+} \rightarrow {}^{3}(PAH - H)^{1+} + {}^{2}H^{0+}$**	**5.3**	**5.3**
	$^{2}PAH^{1+} \rightarrow {}^{2}(PAH - H)^{0+} + {}^{1}H^{1+}$	10.9	10.6
	**$^{1}PAH^{2+} \rightarrow {}^{2}(PAH - H)^{2+} + {}^{2}H^{0+}$**	**5.5**	**5.6**
	$^{1}PAH^{2+} \rightarrow {}^{1}(PAH - H)^{1+} + {}^{1}H^{1+}$	6.9	6.7
	**$^{1}PAH^{2+} \rightarrow {}^{3}(PAH - H)^{1+} + {}^{1}H^{1+}$**	5.8	5.7
	**$^{3}PAH^{2+} \rightarrow {}^{2}(PAH - H)^{2+} + {}^{2}H^{0+}$**	**5.6**	**5.5**
	$^{3}PAH^{2+} \rightarrow {}^{1}(PAH - H)^{1+} + {}^{1}H^{1+}$	7.1	6.6
	$^{3}PAH^{2+} \rightarrow {}^{3}(PAH - H)^{1+} + {}^{1}H^{1+}$	5.9	5.6

Supplementary Table S21: Dissociation energies of proton or hydrogen atom loss from position #5 in FLU @ UKS-DFT/def2-TZVPP level of theory. All values were obtained without geometry relaxation. Bold text highlights the lowest energy dissociation path for a particular PAH charge state and spin state.

Position	Reaction	E_{diss}, eV	
		ωB97	M062x
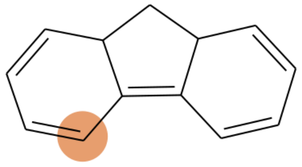	$\mathbf{^{1}PAH^{0+} \rightarrow {}^{2}(PAH - H)^{0+} + {}^{2}H^{0+}}$	**5.2**	**5.2**
	$^{2}PAH^{1+} \rightarrow {}^{1}(PAH - H)^{1+} + {}^{2}H^{0+}$	6.5	6.3
	$\mathbf{^{2}PAH^{1+} \rightarrow {}^{3}(PAH - H)^{1+} + {}^{2}H^{0+}}$	**5.3**	**5.2**
	$^{2}PAH^{1+} \rightarrow {}^{2}(PAH - H)^{0+} + {}^{1}H^{1+}$	10.9	10.6
	$\mathbf{^{1}PAH^{2+} \rightarrow {}^{2}(PAH - H)^{2+} + {}^{2}H^{0+}}$	**5.6**	5.7
	$^{1}PAH^{2+} \rightarrow {}^{1}(PAH - H)^{1+} + {}^{1}H^{1+}$	6.9	6.7
	$\mathbf{^{1}PAH^{2+} \rightarrow {}^{3}(PAH - H)^{1+} + {}^{1}H^{1+}}$	5.7	**5.6**
	$\mathbf{^{3}PAH^{2+} \rightarrow {}^{2}(PAH - H)^{2+} + {}^{2}H^{0+}}$	**5.8**	5.6
	$^{3}PAH^{2+} \rightarrow {}^{1}(PAH - H)^{1+} + {}^{1}H^{1+}$	7.1	6.6
	$\mathbf{^{3}PAH^{2+} \rightarrow {}^{3}(PAH - H)^{1+} + {}^{1}H^{1+}}$	5.8	**5.5**

Supplementary Table S22: Dissociation energies of proton or hydrogen atom loss from position #1 in PHE @ UKS-DFT/def2-TZVPP level of theory. All values were obtained without geometry relaxation. Bold text highlights the lowest energy dissociation path for a particular PAH charge state and spin state.

Position	Reaction	E_{diss}, eV	
		ωB97	M062x
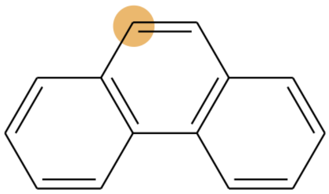	$\mathbf{^{1}PAH^{0+} \rightarrow {}^{2}(PAH - H)^{0+} + {}^{2}H^{0+}}$	**5.2**	**5.2**
	$^{2}PAH^{1+} \rightarrow {}^{1}(PAH - H)^{1+} + {}^{2}H^{0+}$	6.4	6.2
	$\mathbf{^{2}PAH^{1+} \rightarrow {}^{3}(PAH - H)^{1+} + {}^{2}H^{0+}}$	**5.2**	**5.2**
	$^{2}PAH^{1+} \rightarrow {}^{2}(PAH - H)^{0+} + {}^{1}H^{1+}$	10.9	10.7
	$\mathbf{^{1}PAH^{2+} \rightarrow {}^{2}(PAH - H)^{2+} + {}^{2}H^{0+}}$	6.3	**5.2**
	$^{1}PAH^{2+} \rightarrow {}^{1}(PAH - H)^{1+} + {}^{1}H^{1+}$	7.1	6.9
	$\mathbf{^{1}PAH^{2+} \rightarrow {}^{3}(PAH - H)^{1+} + {}^{1}H^{1+}}$	**5.9**	5.8
	$\mathbf{^{3}PAH^{2+} \rightarrow {}^{2}(PAH - H)^{2+} + {}^{2}H^{0+}}$	6.8	**5.5**
	$^{3}PAH^{2+} \rightarrow {}^{1}(PAH - H)^{1+} + {}^{1}H^{1+}$	7.6	7.2
	$\mathbf{^{3}PAH^{2+} \rightarrow {}^{3}(PAH - H)^{1+} + {}^{1}H^{1+}}$	**6.4**	6.1

Supplementary Table S23: Dissociation energies of proton or hydrogen atom loss from position #2 in PHE @ UKS-DFT/def2-TZVPP level of theory. All values were obtained without geometry relaxation. Bold text highlights the lowest energy dissociation path for a particular PAH charge state and spin state.

Position	Reaction	E_{diss}, eV	
		ωB97	M062x
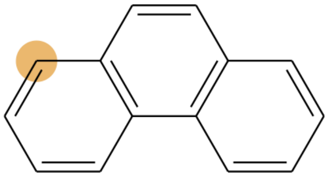	$\mathbf{{}^{1}PAH^{0+} \rightarrow {}^{2}(PAH - H)^{0+} + {}^{2}H^{0+}}$	**5.2**	**5.2**
	${}^{2}\mathrm{PAH}^{1+} \rightarrow {}^{1}(\mathrm{PAH} - \mathrm{H})^{1+} + {}^{2}\mathrm{H}^{0+}$	6.5	6.4
	$\mathbf{{}^{2}PAH^{1+} \rightarrow {}^{3}(PAH - H)^{1+} + {}^{2}H^{0+}}$	**5.2**	**5.2**
	${}^{2}\mathrm{PAH}^{1+} \rightarrow {}^{2}(\mathrm{PAH} - \mathrm{H})^{0+} + {}^{1}\mathrm{H}^{1+}$	10.9	10.7
	$\mathbf{{}^{1}PAH^{2+} \rightarrow {}^{2}(PAH - H)^{2+} + {}^{2}H^{0+}}$	**5.6**	**5.8**
	${}^{1}\mathrm{PAH}^{2+} \rightarrow {}^{1}(\mathrm{PAH} - \mathrm{H})^{1+} + {}^{1}\mathrm{H}^{1+}$	7.2	7.1
	$\mathbf{{}^{1}PAH^{2+} \rightarrow {}^{3}(PAH - H)^{1+} + {}^{1}H^{1+}}$	5.9	**5.8**
	$\mathbf{{}^{3}PAH^{2+} \rightarrow {}^{2}(PAH - H)^{2+} + {}^{2}H^{0+}}$	**6.2**	**6.0**
	${}^{3}\mathrm{PAH}^{2+} \rightarrow {}^{1}(\mathrm{PAH} - \mathrm{H})^{1+} + {}^{1}\mathrm{H}^{1+}$	7.7	7.3
	${}^{3}\mathrm{PAH}^{2+} \rightarrow {}^{3}(\mathrm{PAH} - \mathrm{H})^{1+} + {}^{1}\mathrm{H}^{1+}$	6.4	6.1

Supplementary Table S24: Dissociation energies of proton or hydrogen atom loss from position #3 in PHE @ UKS-DFT/def2-TZVPP level of theory. All values were obtained without geometry relaxation. Bold text highlights the lowest energy dissociation path for a particular PAH charge state and spin state.

Position	Reaction	E_{diss}, eV	
		ωB97	M062x
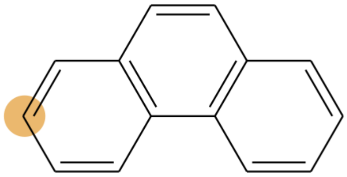	$\mathbf{{}^{1}PAH^{0+} \rightarrow {}^{2}(PAH - H)^{0+} + {}^{2}H^{0+}}$	**5.2**	**5.2**
	${}^{2}\mathrm{PAH}^{1+} \rightarrow {}^{1}(\mathrm{PAH} - \mathrm{H})^{1+} + {}^{2}\mathrm{H}^{0+}$	6.6	6.4
	$\mathbf{{}^{2}PAH^{1+} \rightarrow {}^{3}(PAH - H)^{1+} + {}^{2}H^{0+}}$	**5.4**	**5.3**
	${}^{2}\mathrm{PAH}^{1+} \rightarrow {}^{2}(\mathrm{PAH} - \mathrm{H})^{0+} + {}^{1}\mathrm{H}^{1+}$	10.9	10.7
	$\mathbf{{}^{1}PAH^{2+} \rightarrow {}^{2}(PAH - H)^{2+} + {}^{2}H^{0+}}$	**5.5**	**5.7**
	${}^{1}\mathrm{PAH}^{2+} \rightarrow {}^{1}(\mathrm{PAH} - \mathrm{H})^{1+} + {}^{1}\mathrm{H}^{1+}$	7.2	7.1
	${}^{1}\mathrm{PAH}^{2+} \rightarrow {}^{3}(\mathrm{PAH} - \mathrm{H})^{1+} + {}^{1}\mathrm{H}^{1+}$	6.0	6.0
	$\mathbf{{}^{3}PAH^{2+} \rightarrow {}^{2}(PAH - H)^{2+} + {}^{2}H^{0+}}$	**6.1**	**6.0**
	${}^{3}\mathrm{PAH}^{2+} \rightarrow {}^{1}(\mathrm{PAH} - \mathrm{H})^{1+} + {}^{1}\mathrm{H}^{1+}$	7.8	7.4
	${}^{3}\mathrm{PAH}^{2+} \rightarrow {}^{3}(\mathrm{PAH} - \mathrm{H})^{1+} + {}^{1}\mathrm{H}^{1+}$	6.6	6.3

Supplementary Table S25: Dissociation energies of proton or hydrogen atom loss from position #4 in PHE @ UKS-DFT/def2-TZVPP level of theory. All values were obtained without geometry relaxation. Bold text highlights the lowest energy dissociation path for a particular PAH charge state and spin state.

Position	Reaction	E_{diss}, eV	
		ωB97	M062x
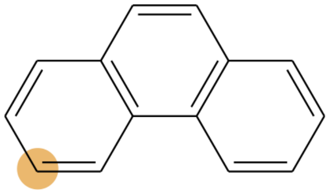	**$^{1}PAH^{0+} \rightarrow {}^{2}(PAH - H)^{0+} + {}^{2}H^{0+}$**	**5.2**	**5.2**
	$^{2}PAH^{1+} \rightarrow {}^{1}(PAH - H)^{1+} + {}^{2}H^{0+}$	6.5	6.4
	**$^{2}PAH^{1+} \rightarrow {}^{3}(PAH - H)^{1+} + {}^{2}H^{0+}$**	**5.2**	**5.2**
	$^{2}PAH^{1+} \rightarrow {}^{2}(PAH - H)^{0+} + {}^{1}H^{1+}$	10.9	10.7
	**$^{1}PAH^{2+} \rightarrow {}^{2}(PAH - H)^{2+} + {}^{2}H^{0+}$**	**5.6**	**5.7**
	$^{1}PAH^{2+} \rightarrow {}^{1}(PAH - H)^{1+} + {}^{1}H^{1+}$	7.2	7.1
	$^{1}PAH^{2+} \rightarrow {}^{3}(PAH - H)^{1+} + {}^{1}H^{1+}$	5.9	5.8
	**$^{3}PAH^{2+} \rightarrow {}^{2}(PAH - H)^{2+} + {}^{2}H^{0+}$**	**6.1**	**6.0**
	$^{3}PAH^{2+} \rightarrow {}^{1}(PAH - H)^{1+} + {}^{1}H^{1+}$	7.7	7.3
	$^{3}PAH^{2+} \rightarrow {}^{3}(PAH - H)^{1+} + {}^{1}H^{1+}$	6.4	6.1

Supplementary Table S26: Dissociation energies of proton or hydrogen atom loss from position #5 in PHE @ UKS-DFT/def2-TZVPP level of theory. All values were obtained without geometry relaxation. Bold text highlights the lowest energy dissociation path for a particular PAH charge state and spin state.

Position	Reaction	E_{diss}, eV	
		ωB97	M062x
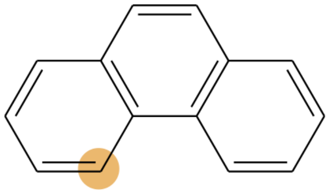	**$^{1}PAH^{0+} \rightarrow {}^{2}(PAH - H)^{0+} + {}^{2}H^{0+}$**	**5.2**	**5.1**
	$^{2}PAH^{1+} \rightarrow {}^{1}(PAH - H)^{1+} + {}^{2}H^{0+}$	6.4	6.3
	**$^{2}PAH^{1+} \rightarrow {}^{3}(PAH - H)^{1+} + {}^{2}H^{0+}$**	**5.2**	**5.2**
	$^{2}PAH^{1+} \rightarrow {}^{2}(PAH - H)^{0+} + {}^{1}H^{1+}$	10.8	10.6
	**$^{1}PAH^{2+} \rightarrow {}^{2}(PAH - H)^{2+} + {}^{2}H^{0+}$**	**5.5**	**5.7**
	$^{1}PAH^{2+} \rightarrow {}^{1}(PAH - H)^{1+} + {}^{1}H^{1+}$	7.1	6.9
	$^{1}PAH^{2+} \rightarrow {}^{3}(PAH - H)^{1+} + {}^{1}H^{1+}$	5.9	5.8
	**$^{3}PAH^{2+} \rightarrow {}^{2}(PAH - H)^{2+} + {}^{2}H^{0+}$**	**6.1**	**5.9**
	$^{3}PAH^{2+} \rightarrow {}^{1}(PAH - H)^{1+} + {}^{1}H^{1+}$	7.6	7.2
	$^{3}PAH^{2+} \rightarrow {}^{3}(PAH - H)^{1+} + {}^{1}H^{1+}$	6.4	6.1

Supplementary Table S27: Dissociation energies of proton or hydrogen atom loss from position #1 in PYR @ UKS-DFT/def2-TZVPP level of theory. All values were obtained without geometry relaxation. Bold text highlights the lowest energy dissociation path for a particular PAH charge state and spin state.

Position	Reaction	E_{diss}, eV	
		ωB97	M062x
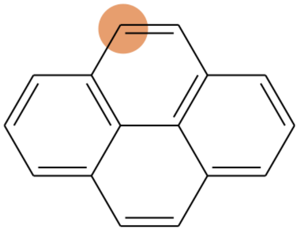	**$^{1}PAH^{0+} \rightarrow {}^{2}(PAH - H)^{0+} + {}^{2}H^{0+}$**	**5.2**	**5.2**
	$^{2}PAH^{1+} \rightarrow {}^{1}(PAH - H)^{1+} + {}^{2}H^{0+}$	7.0	6.8
	**$^{2}PAH^{1+} \rightarrow {}^{3}(PAH - H)^{1+} + {}^{2}H^{0+}$**	**5.3**	**5.2**
	$^{2}PAH^{1+} \rightarrow {}^{2}(PAH - H)^{0+} + {}^{1}H^{1+}$	11.4	11.2
	**$^{1}PAH^{2+} \rightarrow {}^{2}(PAH - H)^{2+} + {}^{2}H^{0+}$**	**6.3**	**6.5**
	$^{1}PAH^{2+} \rightarrow {}^{1}(PAH - H)^{1+} + {}^{1}H^{1+}$	8.3	8.2
	$^{1}PAH^{2+} \rightarrow {}^{3}(PAH - H)^{1+} + {}^{1}H^{1+}$	6.6	6.6
	**$^{3}PAH^{2+} \rightarrow {}^{2}(PAH - H)^{2+} + {}^{2}H^{0+}$**	**5.9**	**5.9**
	$^{3}PAH^{2+} \rightarrow {}^{1}(PAH - H)^{1+} + {}^{1}H^{1+}$	7.8	7.6
	$^{3}PAH^{2+} \rightarrow {}^{3}(PAH - H)^{1+} + {}^{1}H^{1+}$	6.2	6.0

Supplementary Table S28: Dissociation energies of proton or hydrogen atom loss from position #2 in PYR @ UKS-DFT/def2-TZVPP level of theory. All values were obtained without geometry relaxation. Bold text highlights the lowest energy dissociation path for a particular PAH charge state and spin state.

Position	Reaction	E_{diss}, eV	
		ωB97	M062x
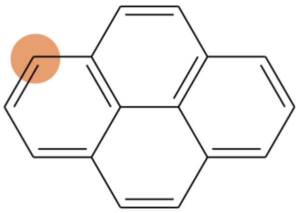	**$^{1}PAH^{0+} \rightarrow {}^{2}(PAH - H)^{0+} + {}^{2}H^{0+}$**	**5.2**	**5.2**
	$^{2}PAH^{1+} \rightarrow {}^{1}(PAH - H)^{1+} + {}^{2}H^{0+}$	6.9	6.8
	**$^{2}PAH^{1+} \rightarrow {}^{3}(PAH - H)^{1+} + {}^{2}H^{0+}$**	**5.2**	**5.2**
	$^{2}PAH^{1+} \rightarrow {}^{2}(PAH - H)^{0+} + {}^{1}H^{1+}$	11.4	11.2
	**$^{1}PAH^{2+} \rightarrow {}^{2}(PAH - H)^{2+} + {}^{2}H^{0+}$**	**6.4**	**6.5**
	$^{1}PAH^{2+} \rightarrow {}^{1}(PAH - H)^{1+} + {}^{1}H^{1+}$	8.3	8.2
	**$^{1}PAH^{2+} \rightarrow {}^{3}(PAH - H)^{1+} + {}^{1}H^{1+}$**	6.6	**6.5**
	**$^{3}PAH^{2+} \rightarrow {}^{2}(PAH - H)^{2+} + {}^{2}H^{0+}$**	**5.9**	**5.9**
	$^{3}PAH^{2+} \rightarrow {}^{1}(PAH - H)^{1+} + {}^{1}H^{1+}$	7.8	7.6
	$^{3}PAH^{2+} \rightarrow {}^{3}(PAH - H)^{1+} + {}^{1}H^{1+}$	6.1	6.0

Supplementary Table S29: Dissociation energies of proton or hydrogen atom loss from position #3 in PYR @ UKS-DFT/def2-TZVPP level of theory. All values were obtained without geometry relaxation. Bold text highlights the lowest energy dissociation path for a particular PAH charge state and spin state.

Position	Reaction	E_{diss}, eV	
		ωB97	M062x
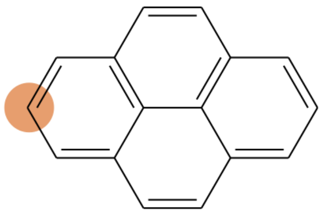	**$^{1}\mathrm{PAH}^{0+} \rightarrow {}^{2}(\mathrm{PAH}-\mathrm{H})^{0+} + {}^{2}\mathrm{H}^{0+}$**	**5.2**	**5.2**
	$^{2}\mathrm{PAH}^{1+} \rightarrow {}^{1}(\mathrm{PAH}-\mathrm{H})^{1+} + {}^{2}\mathrm{H}^{0+}$	7.0	6.8
	**$^{2}\mathrm{PAH}^{1+} \rightarrow {}^{3}(\mathrm{PAH}-\mathrm{H})^{1+} + {}^{2}\mathrm{H}^{0+}$**	**5.4**	**5.3**
	$^{2}\mathrm{PAH}^{1+} \rightarrow {}^{2}(\mathrm{PAH}-\mathrm{H})^{0+} + {}^{1}\mathrm{H}^{1+}$	11.4	11.2
	**$^{1}\mathrm{PAH}^{2+} \rightarrow {}^{2}(\mathrm{PAH}-\mathrm{H})^{2+} + {}^{2}\mathrm{H}^{0+}$**	**6.2**	**6.4**
	$^{1}\mathrm{PAH}^{2+} \rightarrow {}^{1}(\mathrm{PAH}-\mathrm{H})^{1+} + {}^{1}\mathrm{H}^{1+}$	8.3	8.2
	$^{1}\mathrm{PAH}^{2+} \rightarrow {}^{3}(\mathrm{PAH}-\mathrm{H})^{1+} + {}^{1}\mathrm{H}^{1+}$	6.7	6.7
	**$^{3}\mathrm{PAH}^{2+} \rightarrow {}^{2}(\mathrm{PAH}-\mathrm{H})^{2+} + {}^{2}\mathrm{H}^{0+}$**	**5.8**	**5.8**
	$^{3}\mathrm{PAH}^{2+} \rightarrow {}^{1}(\mathrm{PAH}-\mathrm{H})^{1+} + {}^{1}\mathrm{H}^{1+}$	7.8	7.6
	$^{3}\mathrm{PAH}^{2+} \rightarrow {}^{3}(\mathrm{PAH}-\mathrm{H})^{1+} + {}^{1}\mathrm{H}^{1+}$	6.3	6.1

Supplementary Note 2: TOF-TOF Partial Covariance Maps

Experimental TOF-TOF partial covariance maps[9] for FLU (Fig. 5), PHE (Fig. 6), and PYR (Fig. 7). Partial covariance correction accounted for fluctuations of FEL and IR pulse energy. Straight lines with slope of −1 indicate the (1,1) Coulomb explosions[9]

$$\mathrm{PAH^{2+} \rightarrow C_nH_x^+ + (PAH - C_nH_x)^+}$$

with $n = 2, 3, 4$.

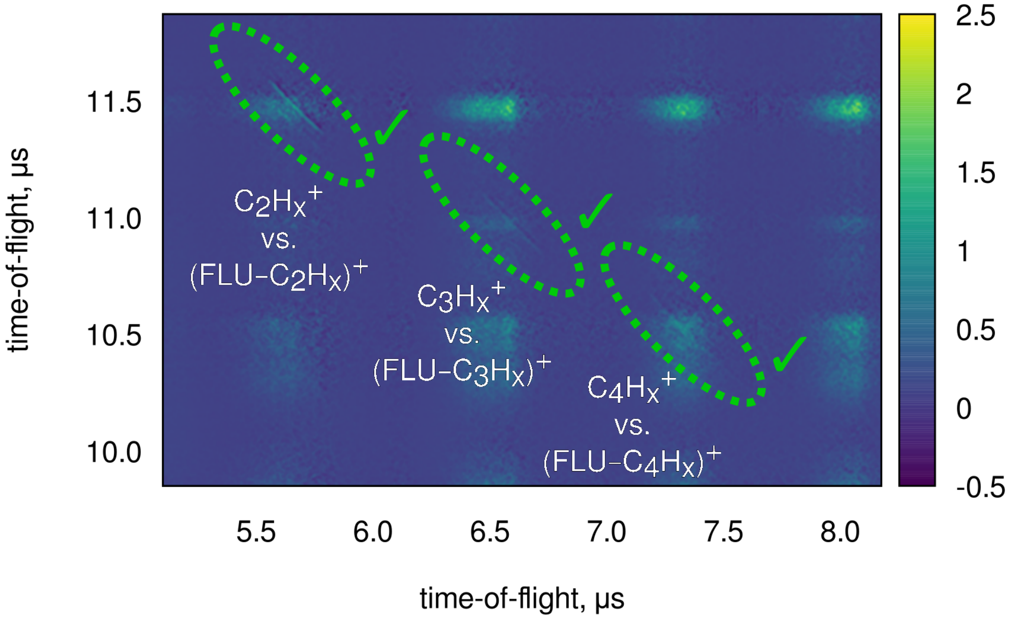

Supplementary Figure 5: FLU partial covariance map.

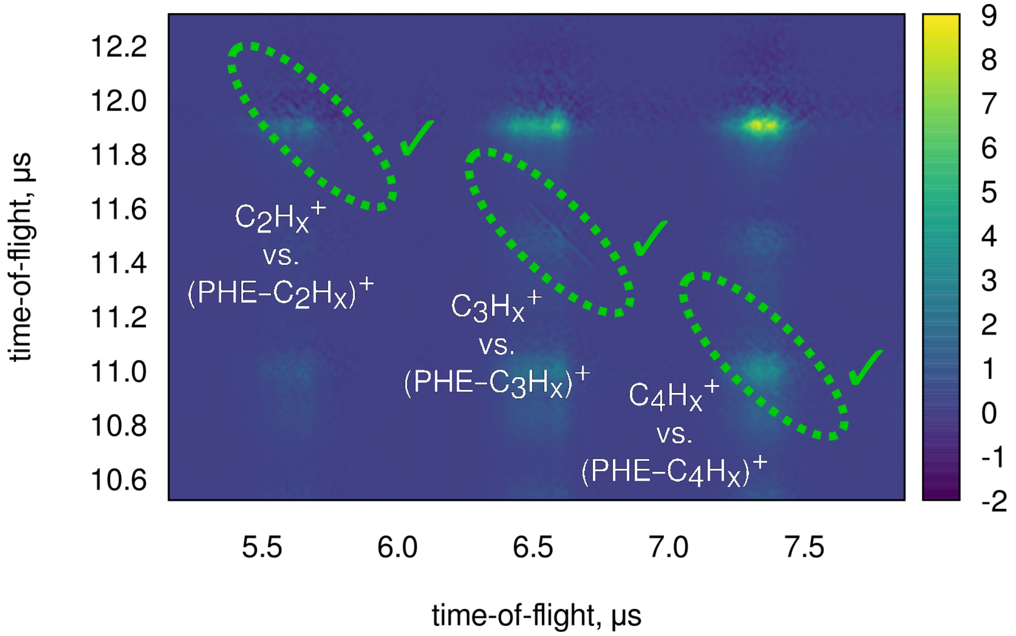

Supplementary Figure 6: PHE partial covariance map.

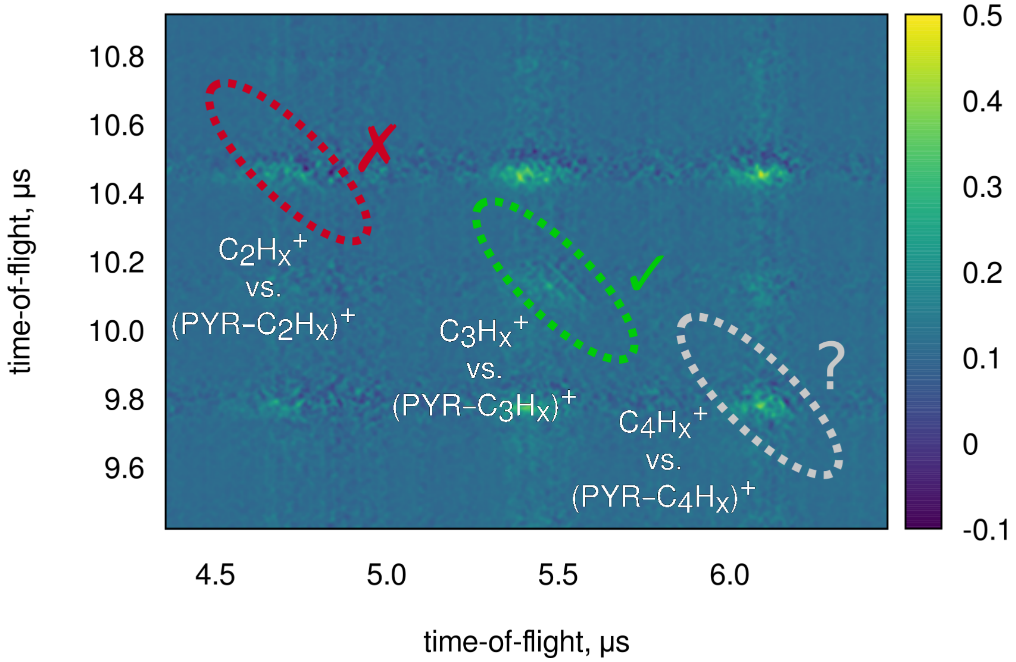

Supplementary Figure 7: PYR partial covariance map.

Supplementary Note 3: PImMS Analysis

3.1 Ion-Ion Recoil Frame Covariances

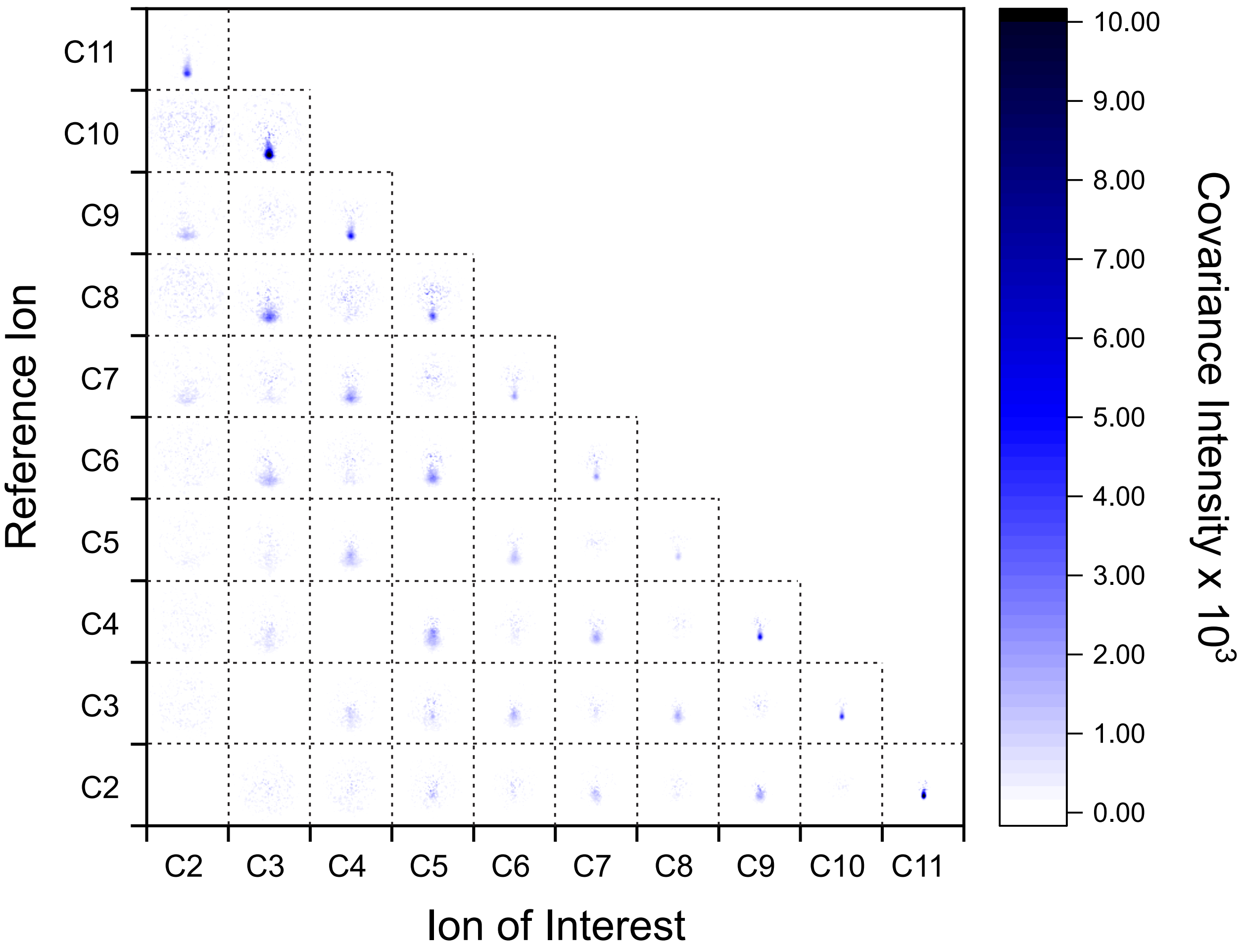

Supplementary Figure 8: Experimental ion-ion recoil frame covariances for all the charged fragments of FLU.

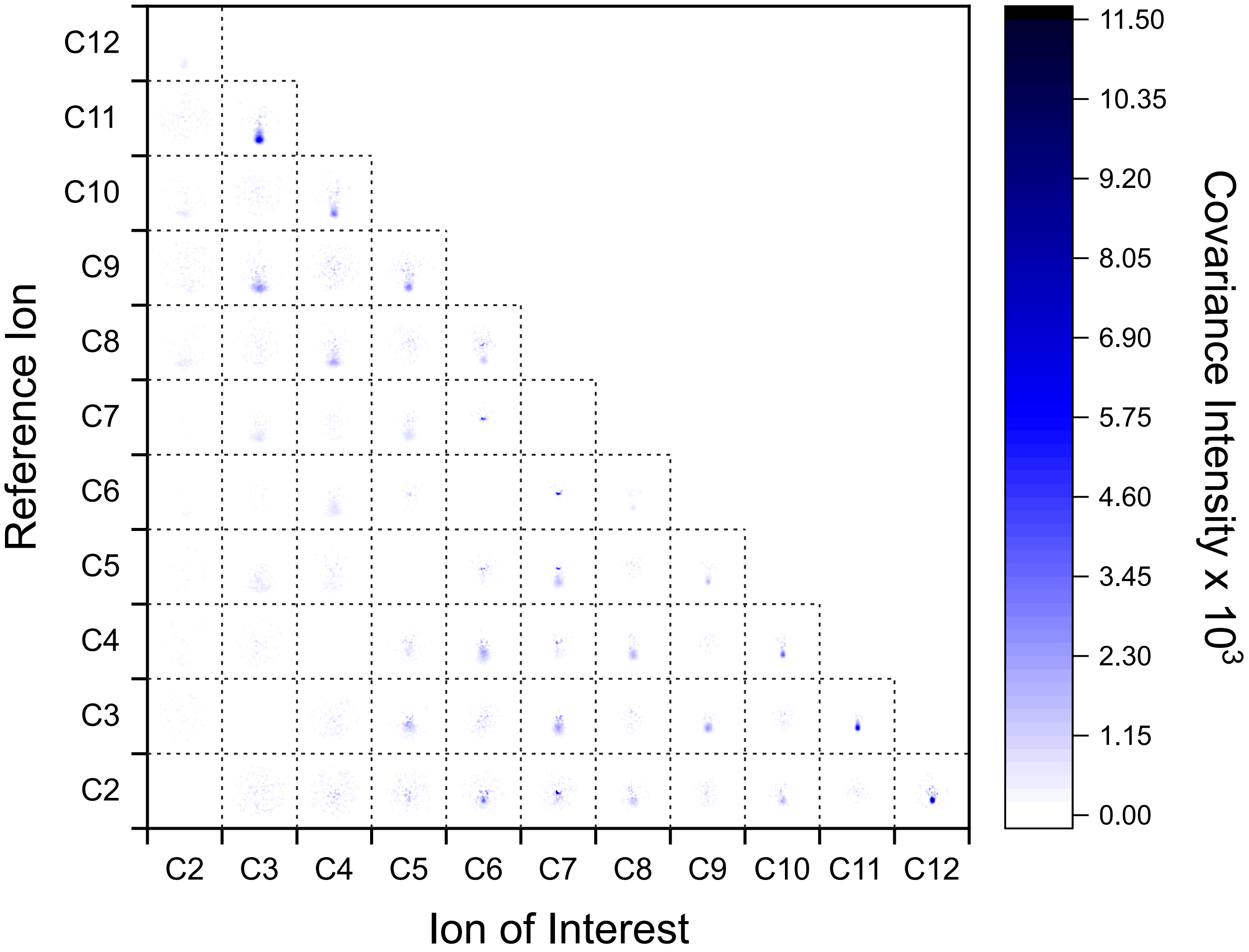

Supplementary Figure 9: Experimental ion-ion recoil frame covariances for all the charged fragments of PHE.

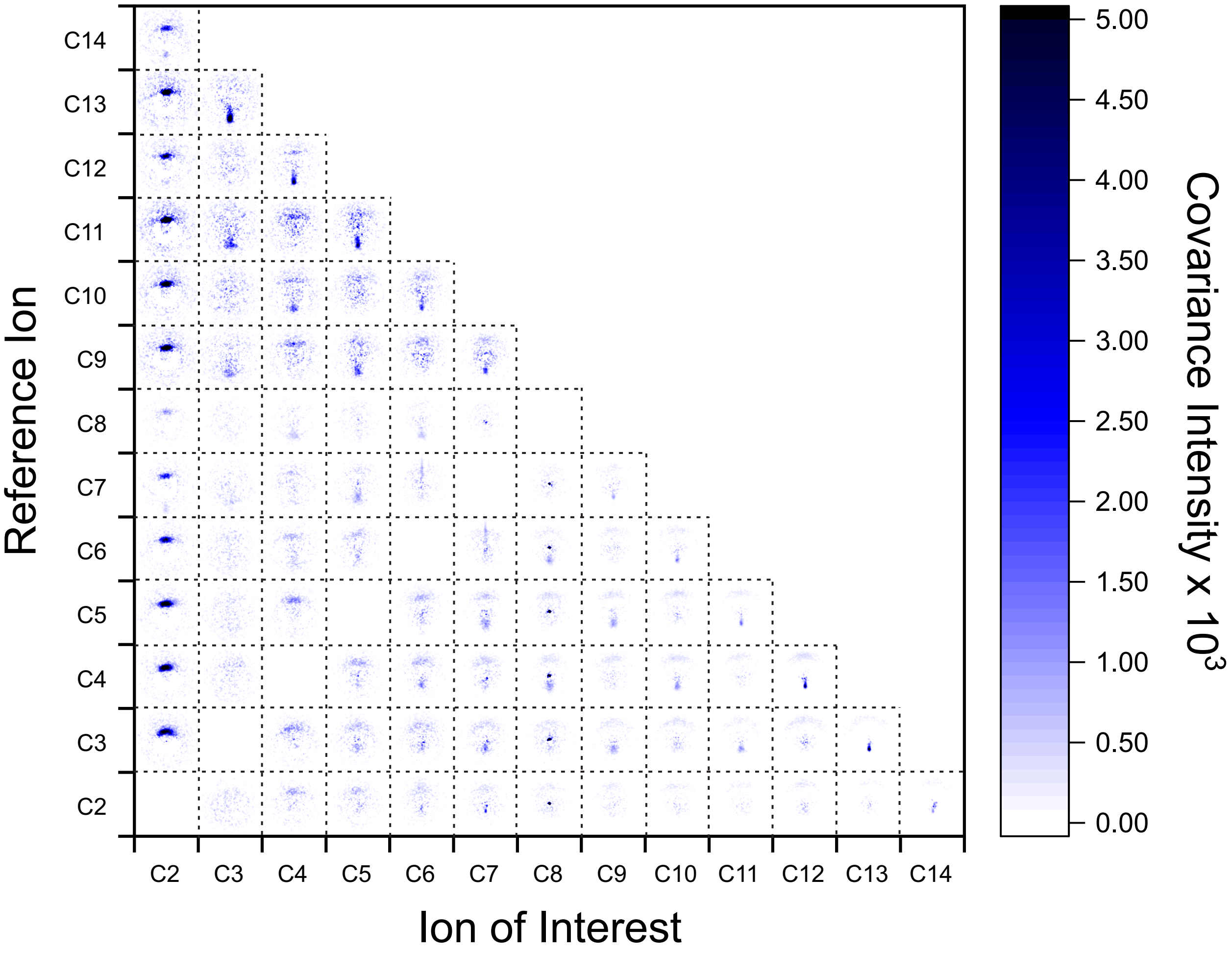

Supplementary Figure 10: Experimental ion-ion recoil frame covariances for all the charged fragments of PYR. Heavy signal in the C2 column is attributed to background N_2^+ ions with a similar value of m/z.

3.2 $C_3H_x^+$ Ion Momentum Maps

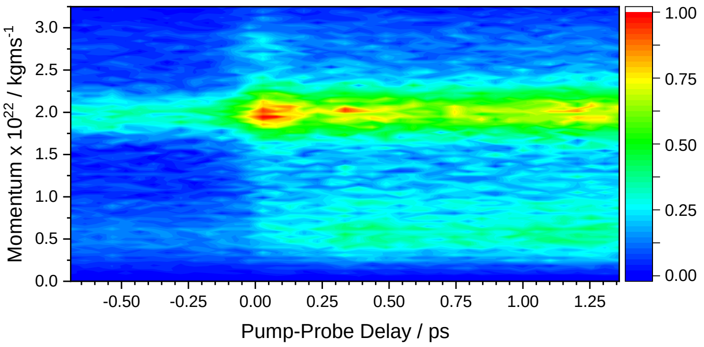

(a) FLU

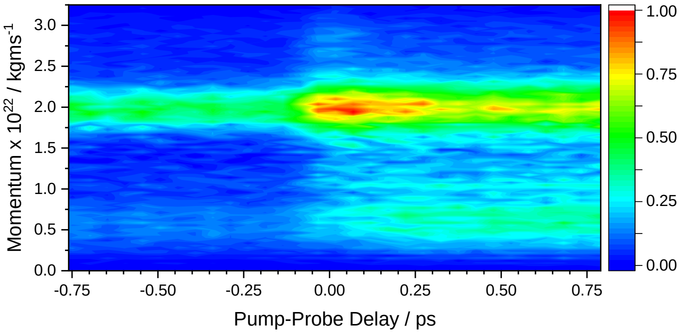

(b) PHE

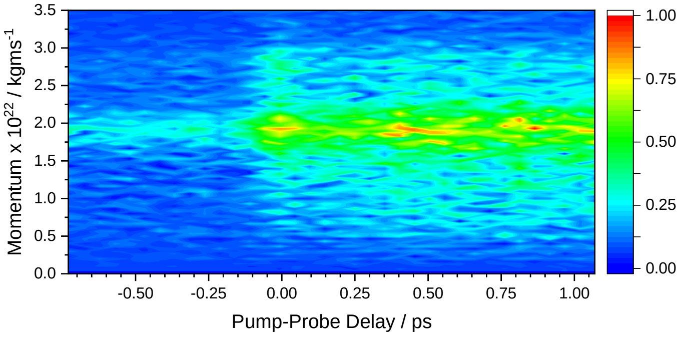

(c) PYR

Supplementary Figure 11: Experimental momentum maps for $C_3H_x^+$ ions formed from the noted parent ions. Ions are recorded using the PImMS sensor, Abel-inverted using the polar-onion peeling method, and an angular integration is performed.

Supplementary Note 4: Photoelectron Spectra and t_0 Estimation

Photoelectron spectra (PES) were recorded for all three molecules using a VMI spectrometer. The results are shown in Fig. 12. Panel (a) contains angularly and delay integrated PES, and panel (b) shows an example of a delay integrated electron VMI image (PHE) following Abel-inversion.[10] The main feature in spectrum is the He (1s) photoelectron line at a kinetic energy of 16.3 eV by ionization of the helium carrier gas used for the molecular beam, the molecular photoelectrons from the singly charged PAHs at higher kinetic energy and molecular photoelectrons from the multiply charged PAHs at lower kinetic energy. The resolution of the electron spectra is estimated to be around 0.5 eV. For further discussion of the pump probe effect, the electron spectrum was divided into several regions: zero kinetic energy electrons, low kinetic energy electrons (LKE e^-), *i.e.* below 2 eV, and the helium photoelectron line with sidebands induced by the IR pulse.

The time t_0, when the pump laser pulse, the probe laser pulses and the molecular beam have spatial and temporal overlap, was estimated by analysis of the He(1s) photoelectron line, and the first He sideband in the photoelectron spectra. At t_0, there is a concurrent depletion of the He(1s) photoelectron line and increase in signal of the He(1s) 1st sideband photoelectron line. The pump-probe behavior was fit with the Gaussian function $\exp\left(-\frac{(t-t_0)^2}{\tau_{\mathrm{cc}}}\right)$, where $\tau_{\mathrm{cc}} = 2\sqrt{\ln(2)}\mathrm{FWHM}_{\mathrm{cc}}$ is the cross-correlation width related to the cross-correlation FWHM as given.

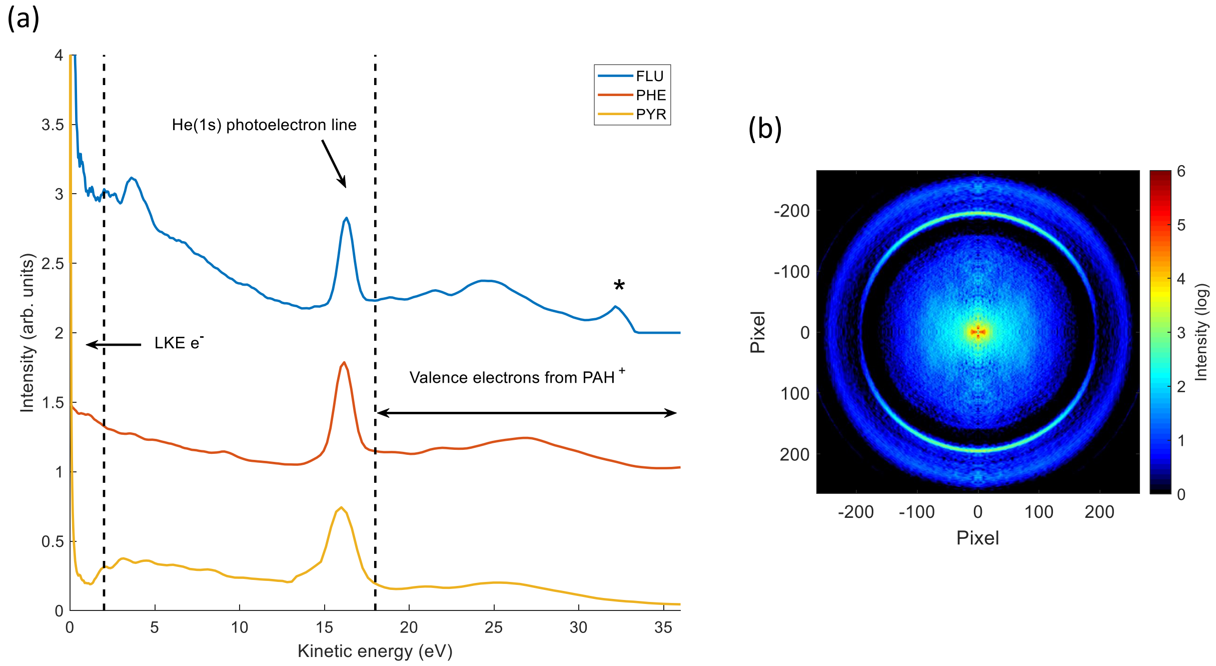

Supplementary Figure 12: (a) The delay integrated photoelectron spectra of FLU (blue), PHE (red) and PYR (yellow). The * symbol indicates the edge of the detector. (b) An example of a delay integrated electron VMI image for PHE following Abel-inversion.

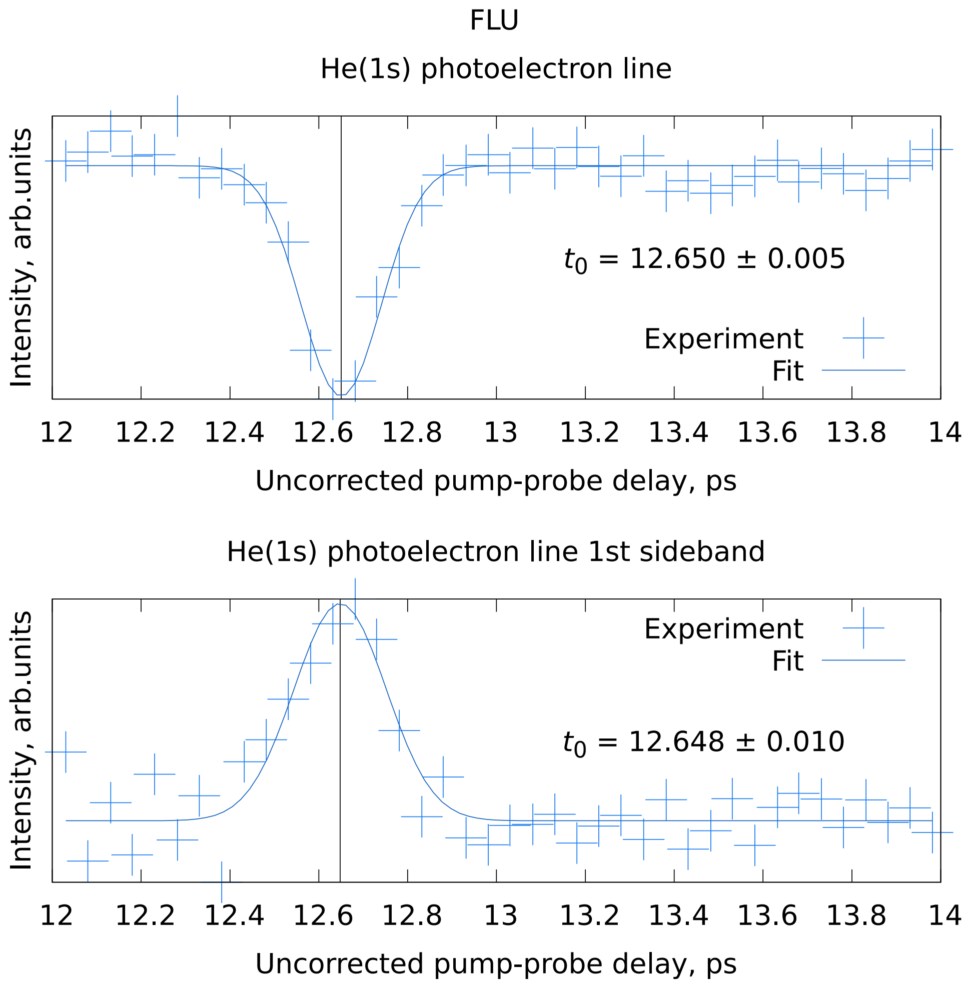

Supplementary Figure 13: t_0 estimation from the Helium (1s) photoelectron line and the first sideband for FLU.

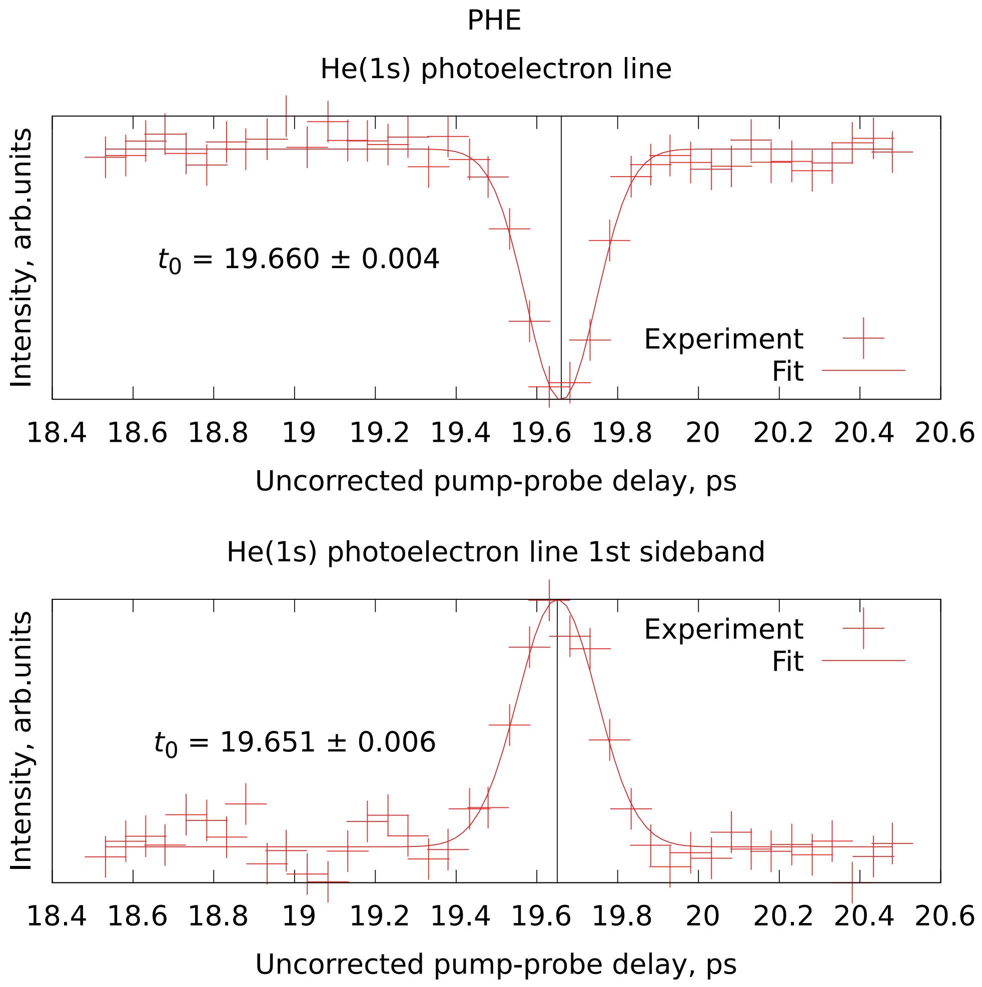

Supplementary Figure 14: t_0 estimation from the Helium (1s) photoelectron line and the first sideband for PHE.

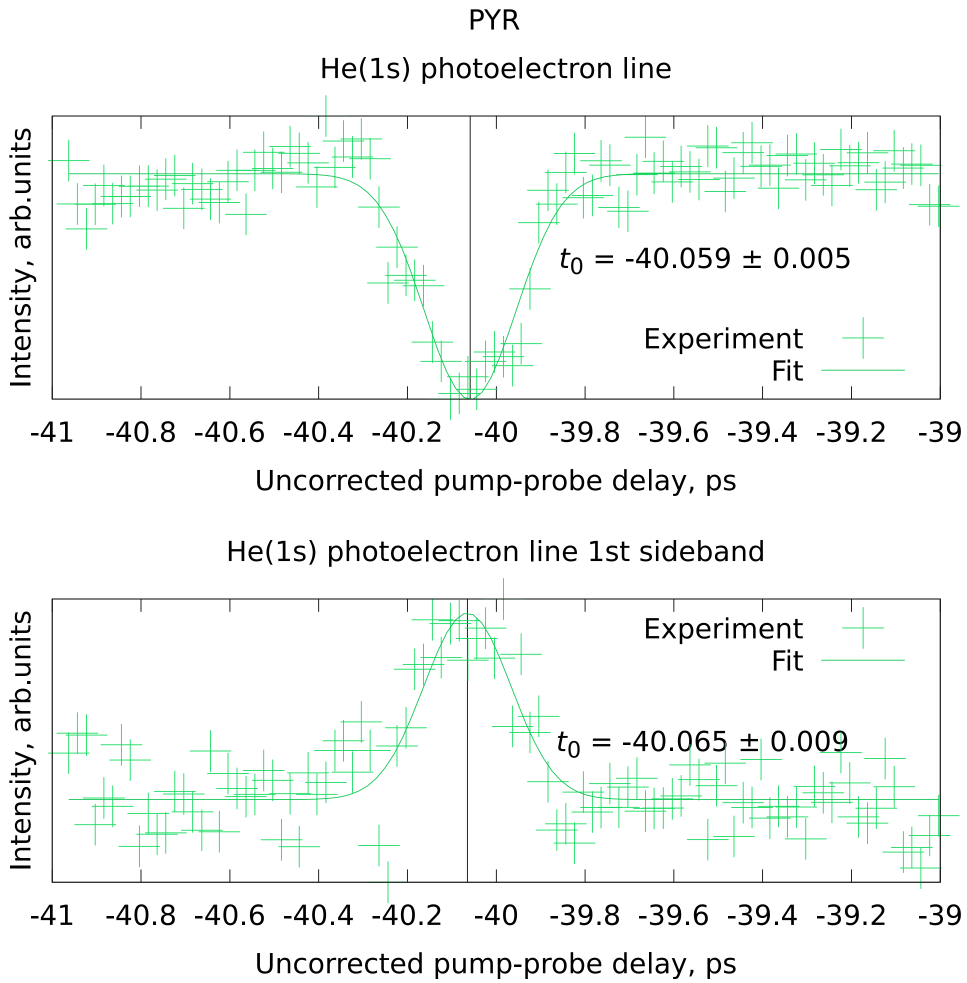

Supplementary Figure 15: t_0 estimation from the Helium (1s) photoelectron line and the first sideband for PYR.

Supplementary Note 5: IR Pulse Only, XUV Pulse Only, and IR-XUV Mass Spectra Comparison

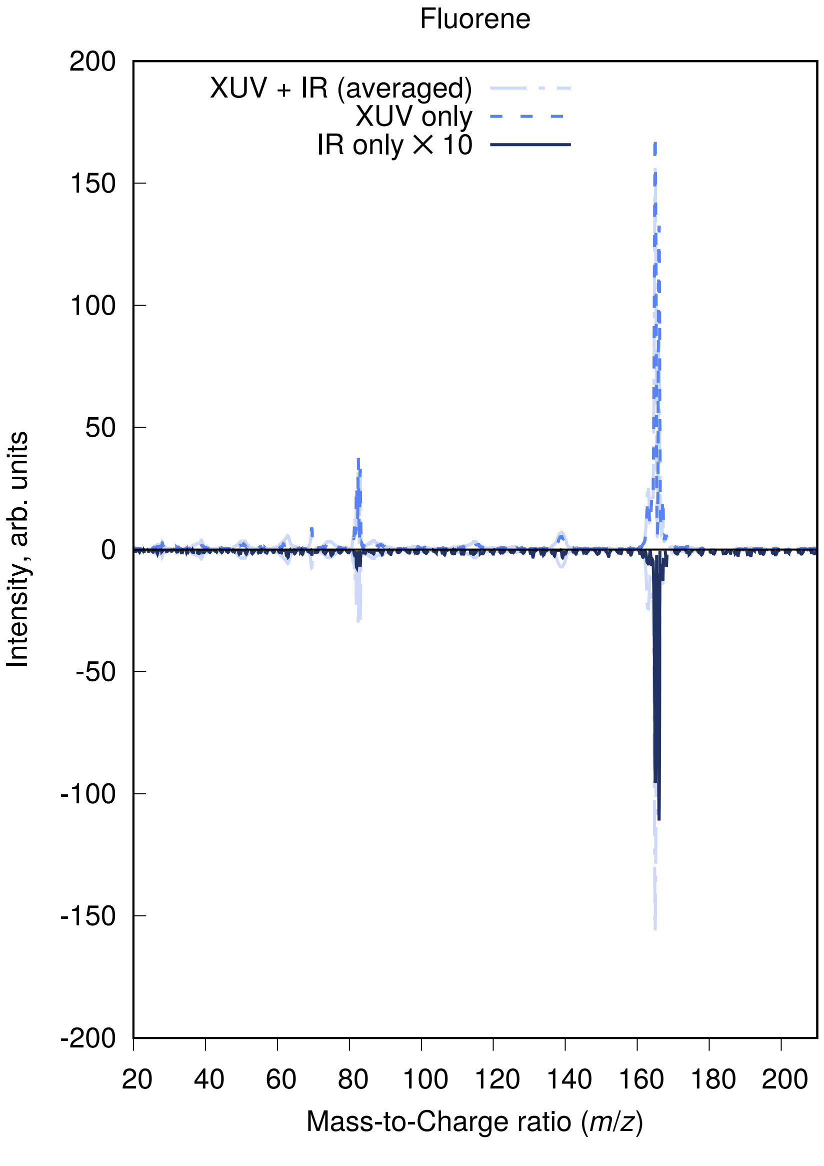

Supplementary Figure 16: Comparison of mass spectra for FLU recorded with the IR pulse only, the XUV pulse only and the average spectrum from the IR/XUV pulse regime.

Supplementary Note 6: Fitting the Pump-Probe Data

The major reaction pathway is for XUV-pump, IR-probe:

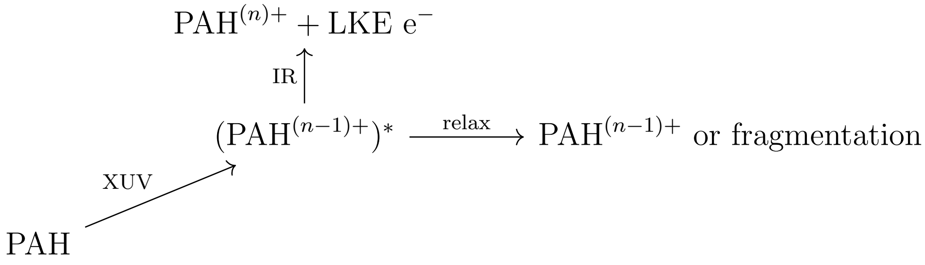

The transient increase in this case[11] will be given as:

$$I_{\mathrm{peak}}(t,\tau_{\mathrm{r}},\tau_{\mathrm{cc}}) = (\theta(t)\cdot\exp\left(-\frac{t}{\tau_{\mathrm{r}}}\right)) * f_{\mathrm{cc}}(t) = \exp\left(\frac{\tau_{\mathrm{cc}}^2}{4\tau_{\mathrm{r}}^2}\right)\cdot\exp(-\frac{t}{\tau_{\mathrm{r}}})\cdot\left[1+\mathrm{erf}\left(\frac{t}{\tau_{\mathrm{cc}}}-\frac{\tau_{\mathrm{cc}}}{2\tau_{\mathrm{r}}}\right)\right],$$

where "*" denotes convolution, $\theta(t) = \begin{cases} 0, t < 0 \\ 1, t \geq 0 \end{cases}$ is the Heaviside step function, and

$$f_{\mathrm{cc}}(t) = \frac{2}{\sqrt{\pi}\tau_{\mathrm{cc}}}\exp\left(-\frac{t^2}{\tau_{\mathrm{cc}}^2}\right)$$

is the cross-correlation function of the two signals, with the cross-correlation time being

$$\tau_{\mathrm{cc}} = \sqrt{\frac{\tau_{\mathrm{XUV}}^2}{n_{\mathrm{XUV}}} + \frac{\tau_{\mathrm{IR}}^2}{n_{\mathrm{IR}}} + \tau_{\mathrm{jitter}}^2}$$

a combination of laser pulse widths (τ_{XUV}, τ_{IR}), instrumental jitter (τ_{jitter}), and the number of photons participating in the process (n_{XUV} and n_{IR}).

The time independent switch of the mechanisms from "pump before probe" and "probe before pump" is given by the function:[11]

$$I_{\mathrm{change}}(t,\tau_{\mathrm{cc}}) = \theta(t) * f_{\mathrm{cc}}(t) = \left[1+\mathrm{erf}\left(\frac{t}{\tau_{\mathrm{cc}}}\right)\right],$$

which leads to an approximation of the signal with an expression:

$$I(\Delta t) = A_0 + A_1 \cdot I_{\text{change}}(\Delta t, \tau_{\text{cc}}) + A_2 \cdot I_{\text{peak}}(\Delta t, \tau_{\text{r}}, \tau_{\text{cc}}) \ ,$$

with $\Delta t = t - t_0$ (corrected pump-probe delay with zero when XUV and IR pulses arrive at the same time), $\{A_n\}_{n=0}^{2}$ are the fitted coefficients. However, this expression does not properly describe the experimental data for dications PAH^{2+} due to a significant contribution from the IR-pump, XUV probe regime:

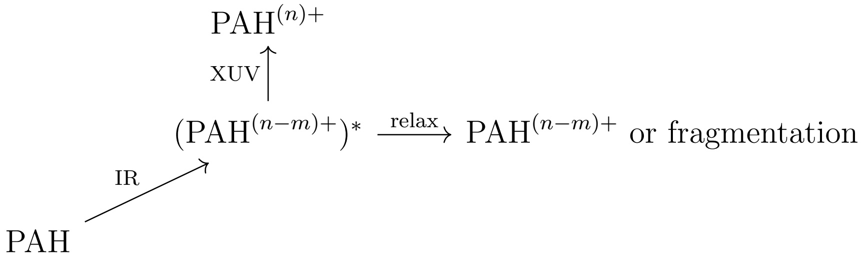

The two pathways should be described by the following parameters:

- $\tau_{\text{r},\rightarrow}$ for the first process (XUV pulse as a pump),
- $\tau_{\text{r},\leftarrow}$ for the second process (IR pulse as a pump).

Therefore the final expression for the PAH^{2+} can be written as:[11,12]

$$I_{\text{theor}}(\Delta t, \tau_{\text{r},\rightarrow}, \tau_{\text{r},\leftarrow}, \tau_{\text{cc}}, \tau_{\text{cc},\leftarrow}) = A_0 + A_1 \cdot I_{\text{change}}(\Delta t, \tau_{\text{cc}}) + A_2 \cdot I_{\text{peak}}(\Delta t, \tau_{\text{r},\rightarrow}, \tau_{\text{cc}}) + B_2 \cdot I_{\text{peak}}(-\Delta t, \tau_{\text{r},\leftarrow}, \tau_{\text{cc}}) \ ,$$

where τ_{cc} is an effective cross-correlation time averaged over all the processes.

However, the least squares (LSQ) fitting of the experimental data is problematic: it has multiple statistically indistinguishable solutions, and for most of these solutions there are large correlations between fitted parameters. Therefore, instead of the LSQ fitting, a Monte-Carlo sampling of the possible solutions was made using the Metropolis algorithm,[13,14] which is a standard approach for such cases.[14–16] The procedure was defined as the following.

- A set of fixed nonlinear parameters $(\tau_{r,\rightarrow}, \tau_{r,\leftarrow}, \tau_{cc}) = \boldsymbol{\tau}$ was randomly generated between lower and upper boundaries. The lower boundary on the decay parameters $\tau_{r,\rightarrow}$ was set to prevent numerical instabilities for I_{peak} function, while the upper one was set to be larger than the experimentally feasible range. The cross-correlation time boundary condition was $\tau_{cc} \in [70, 150]$ (lower boundary given by the XUV pulse duration and jitter, upper boundary by the cross-correlation times measured from the first He sideband, representing a 1 XUV + 1 IR photon process). For this set of nonlinear parameters, a unique set of linear parameters (A_0, A_1, A_2, B_2) was determined from the linear LSQ procedure:[14]

$$\Phi(\boldsymbol{\tau}) = \sum_i \frac{1}{2\sigma_i^2} \left(I_{exp}(\Delta t_i) - I_{theor}(\boldsymbol{\tau})\right)^2 \rightarrow \min ,$$

where $I_{exp}(\Delta t_i)$ is the experimental yield for the i-th pump-probe delay Δt_i, and σ_i is the standard error for this experimental point. The minimal discrepancy functional for the chosen set of parameters is $\min(\Phi(\boldsymbol{\tau})) = \Phi_{min}(\boldsymbol{\tau})$. The probability of the parameters $\boldsymbol{\tau}$ being observed in the experiment was chosen to be the maximum likelihood function:

$$P(\boldsymbol{\tau}) \propto \exp\left(-\Phi_{min}(\boldsymbol{\tau}) - \lambda(\tau_{cc} - \tau_{cc,He})^2 - \frac{\delta t_0^2}{2\sigma_{t_0}^2}\right) , \quad (1)$$

where $\lambda(\tau_{cc} - \tau_{cc,He})^2$ is a regularization term for the cross-correlation time not to deviate too much from the Helium cross-correlation ($\tau_{cc,He}$), $\lambda \geq 0$ is the regularization parameter. δt_0 is a parameter to account for error in the t_0 by substituting t_0 with $t_0 + \delta t_0$. It is sampled from the uniform distribution in the range $\delta t_0 \in [-3\sigma_{t_0}; +3\sigma_{t_0}]$, where σ_{t_0} is a standard deviation for t_0 given by the Least Square fitting procedure given in Section 4.

- The probability for a transition from the parameters $\boldsymbol{\tau}_{old}$ to the new set of parameters

$\boldsymbol{\tau}_{\text{new}}$ is thus given by:

$$P(\boldsymbol{\tau}_{\text{old}} \rightarrow \boldsymbol{\tau}_{\text{new}}) = \min\{1, P(\boldsymbol{\tau}_{\text{new}})/P(\boldsymbol{\tau}_{\text{old}})\} \ .$$

- The length of the simulation was 100,000 points with the first 5,000 points being ignored as an equilibration phase.

The resulting distributions of possible parameters are given in Figs. 17, 18, 19, and expectation values and standard deviations for parameters $\boldsymbol{\tau}$ from these distributions are summarized in Table S30. The comparison of the experimental curves with their possible fits are given in the Fig. 20. Small deviations of the overall ionic/electronic yields (I_{theor}) show that all the solutions give very similar experimental observables. Large deviations in the individual peaks describing the XUV-IR and IR-XUV pump-probe mechanisms for PYR^{2+} in Fig. 19 arise from a strong correlation between two $\leftarrow$ transient functions (see the next paragraph).

The fitting for PAH^{2+}, low kinetic energy electrons (LKE e^-, electrons with kinetic energy below 2 eV), and high kinetic energy (HKE) $C_3H_x^+$ fragments (i.e. (2,1) channel) was performed as follows.

- LKE e^- and HKE $C_3H_x^+$ were fitted using a single channel "XUV pump – IR probe" with parameters $(\tau_{\text{r},\rightarrow}, \tau_{\text{cc}})$.

- PAH^{2+} were fitted with both "XUV pump – IR probe" and "IR pump – XUV probe" channels with parameters $(\tau_{\text{r},\rightarrow}, \tau_{\text{r},\leftarrow,1}, \tau_{\text{cc}})$, the $\tau_{\text{r},\rightarrow}$ was fixed on the value obtained from the LKE e^- fit. The PYR^{2+} ion was fitted with an additional "IR pump – XUV probe" channel described by $\tau_{\text{r},\leftarrow,2}$ constant to account for the slow dynamics in the $t < t_0$ pump-probe range, and with the $\frac{1}{|\tau_{\text{r},\leftarrow,1} - \tau_{\text{r},\leftarrow,2}|}$ regularizing term in Equation 1 to avoid the situations of $\tau_{\text{r},\leftarrow,1} \approx \tau_{\text{r},\leftarrow,2}$ leading to numerical instabilities. The slow dynamics were reproduced over multiple measurements.

Supplementary Table S30: Decay times and cross-correlation times for XUV and IR lasers determined from pump-probe curves for the PAH^{2+}, LKE e^-, and HKE $C_3H_x^+$ (i.e., (2,1) channel). All values are in fs.

Parameter	FLU	PHE	PYR
PAH^{2+}			
$\tau_{r,\leftarrow,1}$	35 ± 8	29 ± 17	62 ± 71
$\tau_{r,\leftarrow,2}$	—	—	900 ± 229
τ_{cc}	97 ± 7	96 ± 12	124 ± 8
LKE e^-			
$\tau_{r,\rightarrow}$	57 ± 13	76 ± 14	24 ± 11
τ_{cc}	113 ± 8	107 ± 7	136 ± 10
HKE $C_3H_x^+$			
$\tau_{r,\rightarrow}$	17 ± 5	74 ± 19	28 ± 10
τ_{cc}	95 ± 2	78 ± 8	103 ± 7

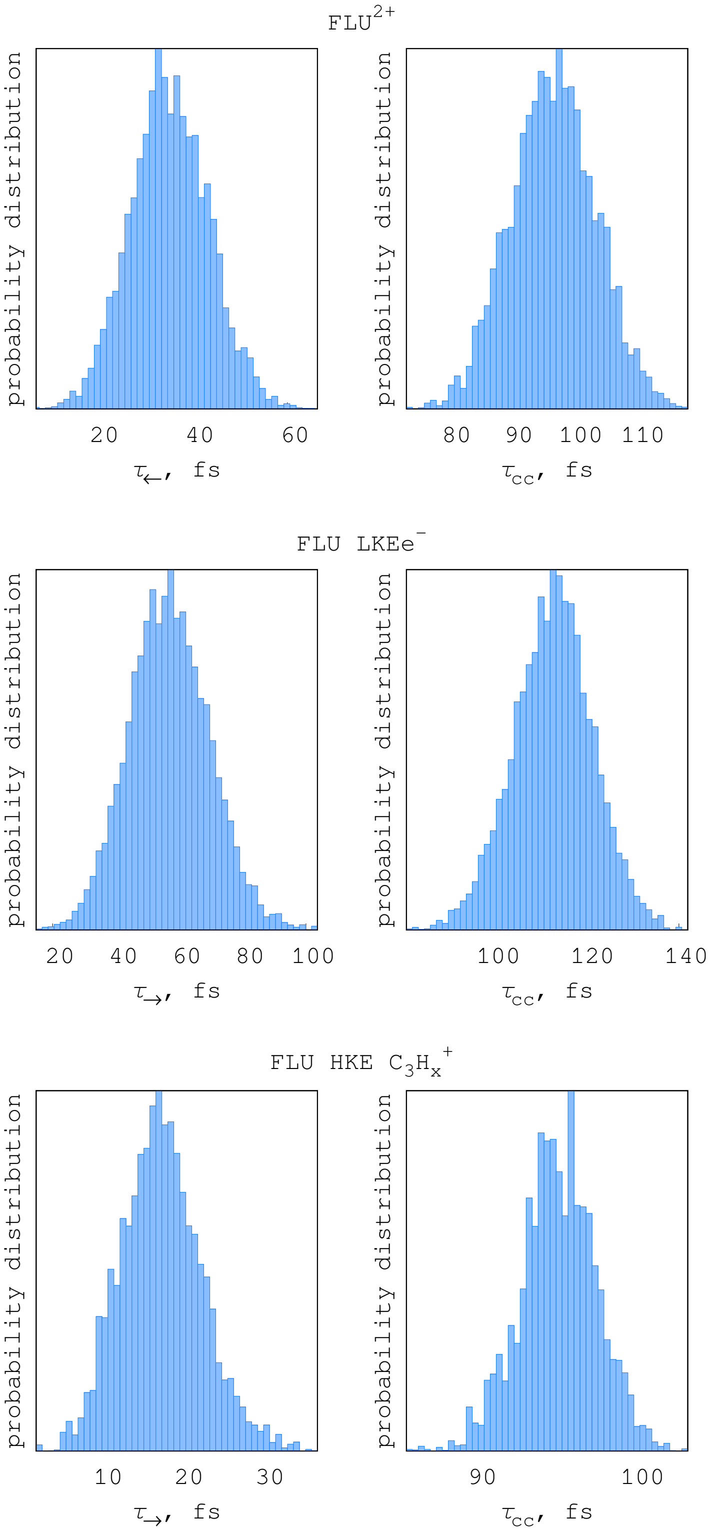

Supplementary Figure 17: Distribution of the model parameters determined with the Metropolis algorithm in the FLU experiment for the PAH^{2+}, LKE e^-, and $C_3H_x^+$ observables.

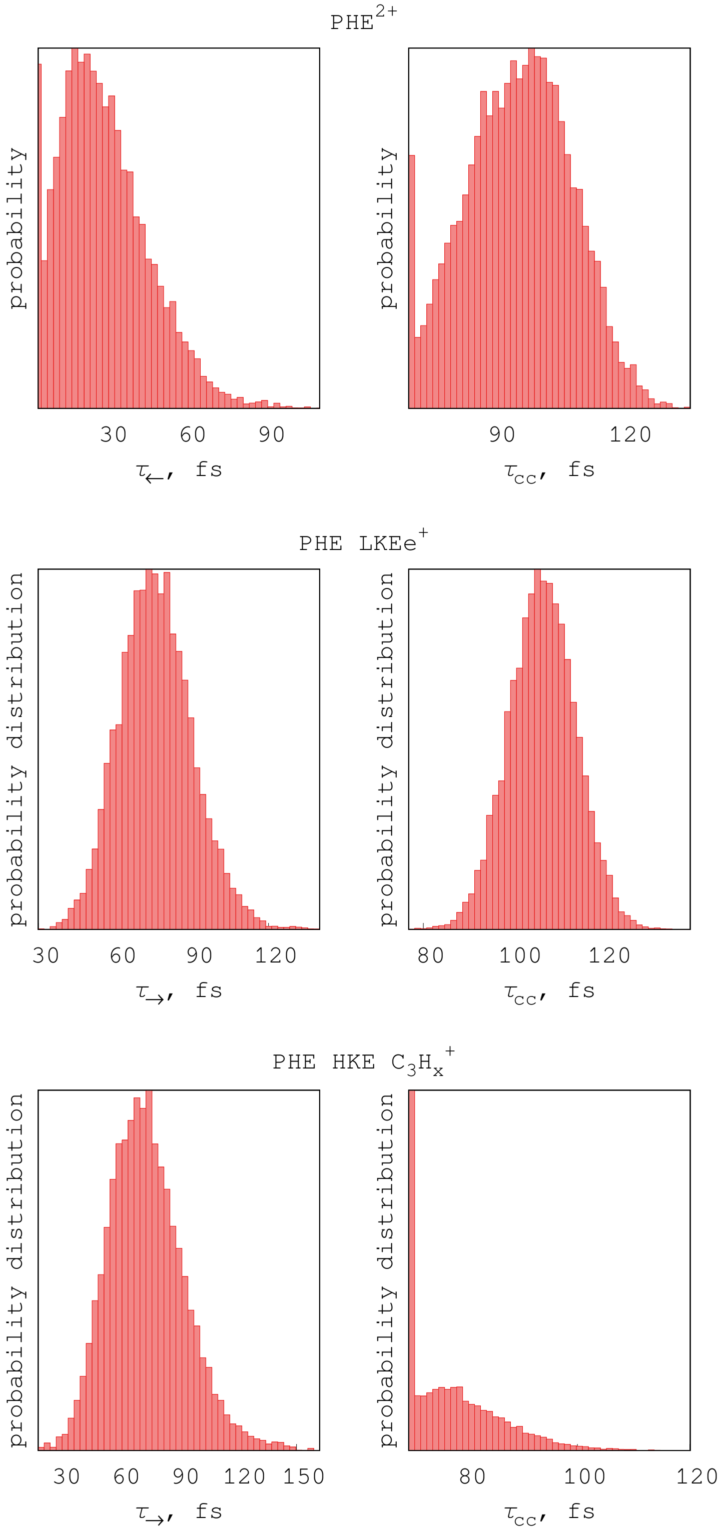

Supplementary Figure 18: Distribution of the model parameters determined with the Metropolis algorithm in the PHE experiment for the PAH^{2+}, LKE e^-, and $C_3H_x^+$ observables.

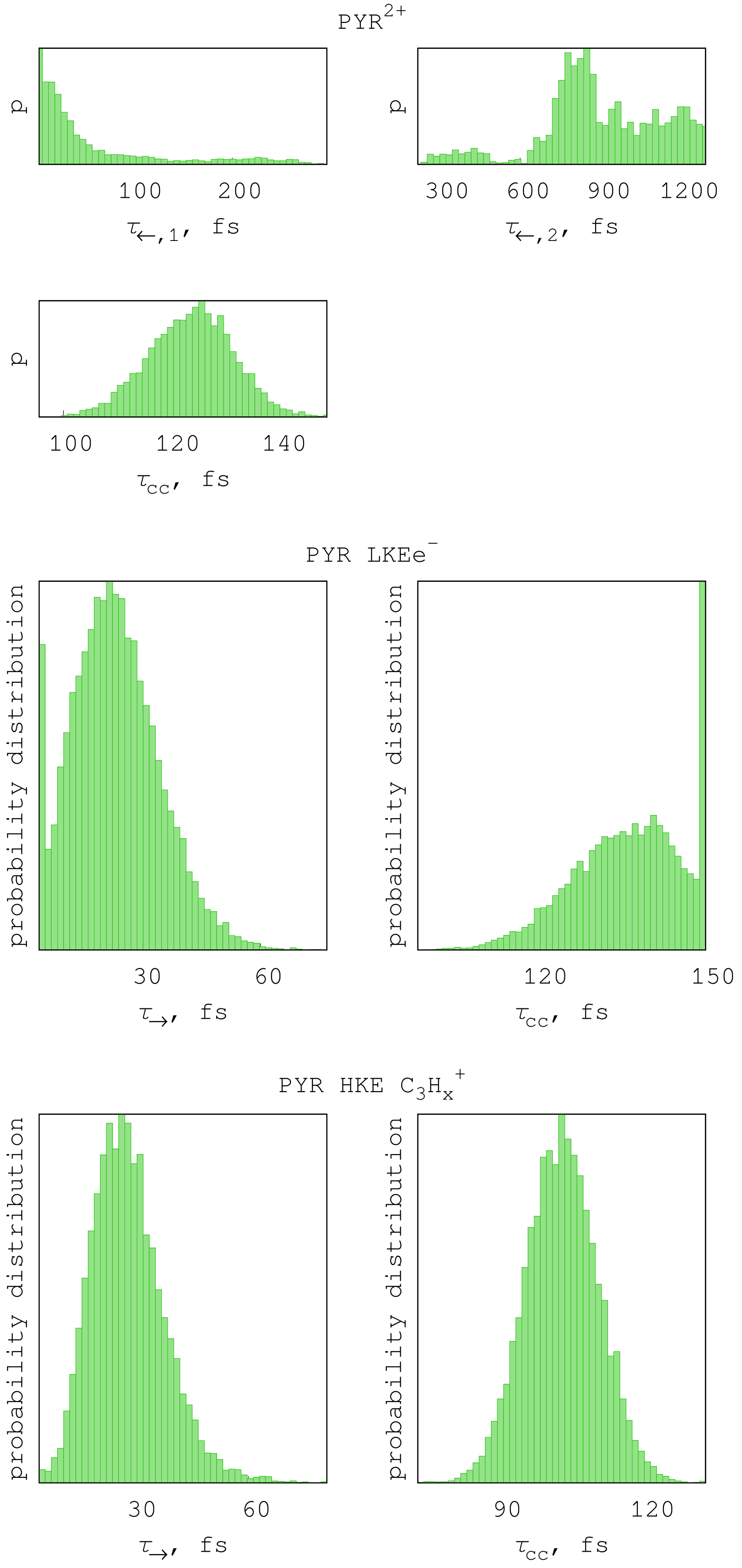

Supplementary Figure 19: Distribution of the model parameters determined with the Metropolis algorithm in the PYR experiment for the PAH^{2+}, LKE e^{-}, and $C_3H_x^+$ observables.

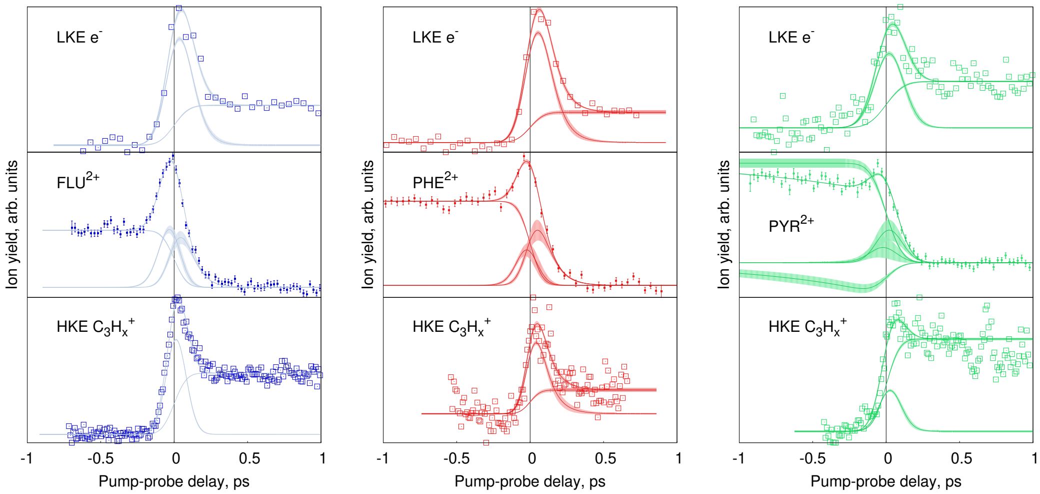

Supplementary Figure 20: Pump-probe signals for PAH^{2+}, LKE e^- and HKE, i.e. (2,1) channel, $C_3H_x^+$. Each pump – probe delay point for PAH^{2+} consists in average of 1300 ± 200, 2000 ± 400, and 900 ± 400 independent data points for FLU, PHE, and PYR, respectively.

Supplementary Note 7: Laser Settings

Supplementary Table S31: Laser settings in the time-resolved pump-probe experiment.

λ_{XUV}	30.3 nm ($h\nu$ = 40.8 eV)
λ_{IR}	810 nm ($h\nu$ = 1.53 eV)
XUV pulse energy	14 μJ, approximately 3.3 μJ after filters
IR pulse energy	1–50 μJ
XUV pulse duration (FWHM)	90 fs
IR pulse duration (FWHM)	60 fs
XUV repetition rate	10 Hz
IR repetition rate	10 Hz

Supplementary Note 8: Trajectory surface hopping molecular dynamics simulations of fluorene

8.1 General considerations

Trajectory surface hopping molecular dynamics (TSH-MD) simulations[17] of fluorene (FLU) were performed using SHARC[18–20] interfaced with Orca 4.[5] All simulations were performed with or based on calculations at the PBE/def2-SV(P) level of theory.[21,22] Excited states were calculated with TD-DFT at the chosen level of theory using the Tamm-Dancoff approximation (TDA). To speed up the (TD-)DFT calculations, the resolution-of-identity (RI) approximation was used.[6–8] Some simulations were done using linear vibronic coupling (LVC) model,[23] as implemented in SHARC.[24] If not explicitly mentioned, the initial conditions for trajectories were sampled from the Wigner distribution of the ground vibrational state of neutral FLU using a harmonic approximation at the PBE/def2-SV(P) level of theory.

8.2 Simulation of NIR pump – XUV probe channel with FLU* as an intermediate

8.2.1 Model background

The first process simulated was the following reaction scheme:

$$\mathrm{FLU} + \mathrm{NIR\ photon} \rightarrow \mathrm{FLU}^*$$

$$\mathrm{FLU}^* \rightarrow \mathrm{FLU}_{\mathrm{hot}}$$

$$\mathrm{FLU}^* + \mathrm{XUV\ photon} \rightarrow \mathrm{FLU}^{n+,*} + n \cdot \mathrm{e}^-$$

The first reaction denotes the interaction of the 810 nm near-IR (NIR) probe laser acting as pump, producing electronically excited FLU*. The second reaction in the scheme shows internal conversion, where the electronic excitation is converted through conical intersections and avoided crossings into vibrational excitation of the ground or a low-lying electronic state. The last reaction shows the FEL XUV pulse probing FLU*, producing the ionized molecular ions $FLU^{n+,*}$ by removal of n electrons.

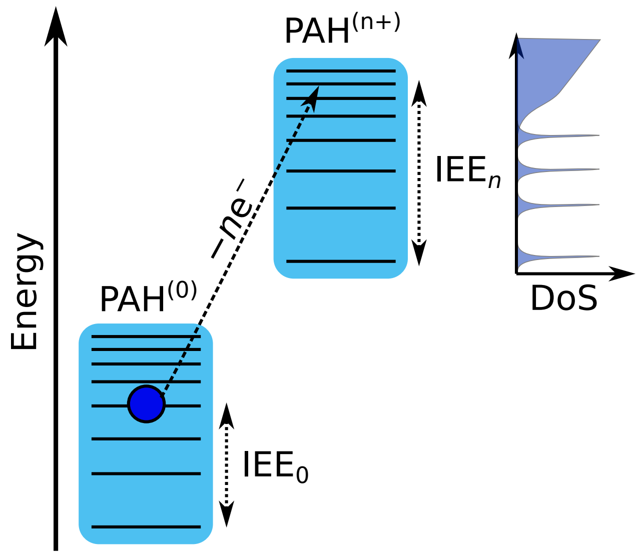

Supplementary Figure 21: Scheme showing the electronically excited neutral PAH formed from the NIR pulse and probed by the XUV pulse to produce the $PAH^{n+,*}$ state.

8.2.2 Simulation of the NIR laser excitation

To simulate the laser excitation, we employed a LVC model of neutral FLU that included 10 excited singlet and 10 excited triplet states. The modeling was performed with the explicit presence of a Gaussian-shaped 810 nm laser pulse (FWHM = 60 fs) with a peak intensity of 10^{13} W/cm^2. Simulations were performed for three possible polarization directions of the laser in the FLU molecular frame (along a, b, and c axis), each polarization had 1000 trajectories. The trajectories were 200 fs long with a time step of 0.5 fs and 25 intermediate steps for electronic wavefunction propagation, the laser pulse was centered at 100 fs. The simulation results are given in Fig. 22. As one can see, the 810 nm laser pulse excites the

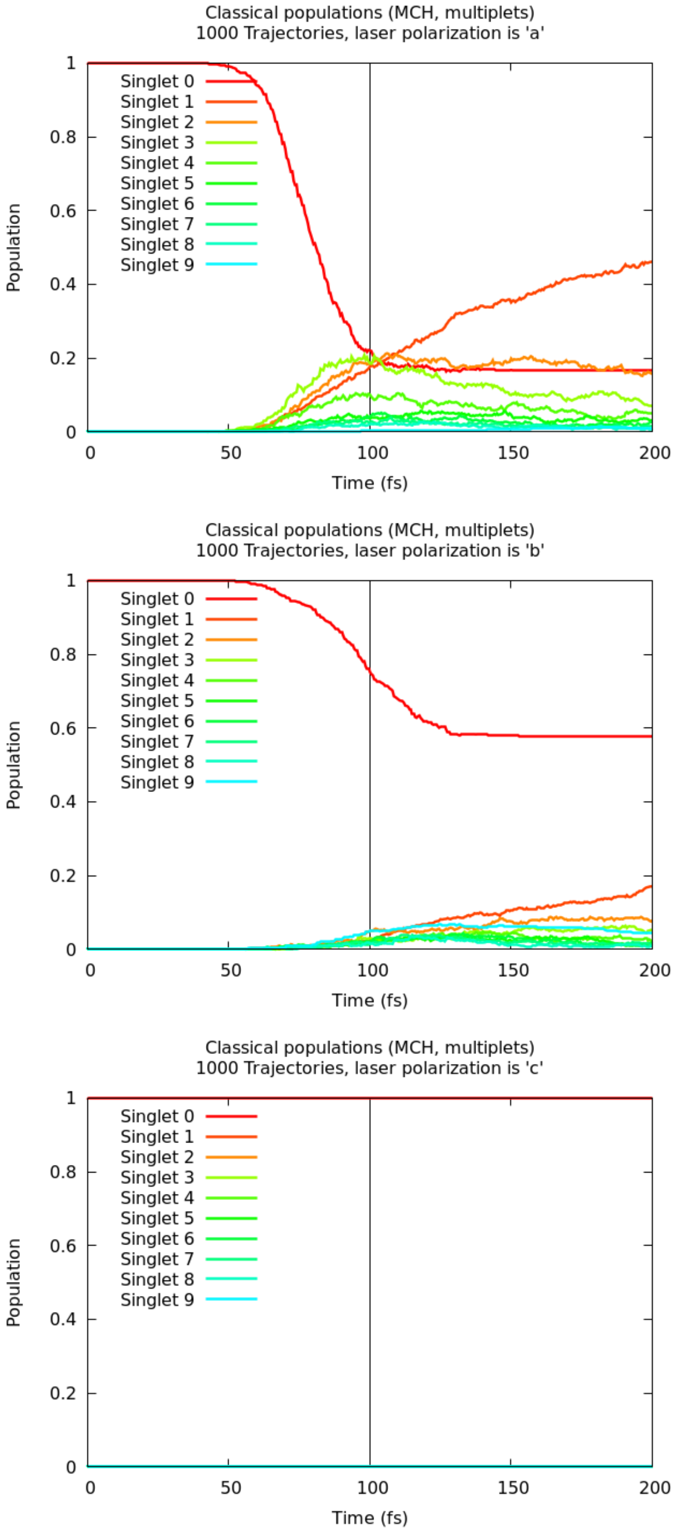

Supplementary Figure 22: TSH-MD simulation results of explicit 810 nm pulse excitation at different polarization axes in the molecular frame. Details are given in the text.

molecule into the lowest singlet excited states, which are of $\pi \rightarrow \pi^*$ character in FLU. This results in excitation anisotropy, which shows maximal excitation efficiency along the a-axis of FLU (where the π-system length is the largest, three fused rings), little excitation efficiency along the b-axis (one fused ring), and no excitation along the c-axis, for which symmetry $\pi \rightarrow \pi^*$ transitions are forbidden. Triplet states were not excited to and not populated afterwards. Further TSH-MD simulations based on on-the-fly TD-DFT calculations for the extraction of the rate constants used the ratio of excited states at 100 fs of simulation. The results are given in Table S32.

Supplementary Table S32: The population of different electronic states resulting from the TSH-MD simulation of an explicit 810 nm pulse excitation at different polarization axes of the molecular frame. Singlet states are denoted as S_n, where n is the state number, S_0 is the ground electronic state. Details are given in the text. All the values are in %.

State	Polarization			
	a	b	c	average
S_0	22	75	100	66
S_1	18	2	0	7
S_2	19	5	0	8
S_3	19	3	0	7
S_4	10	2	0	4
S_5	4	2	0	2
S_6	4	2	0	2
S_7	3	2	0	1
S_8	2	2	0	1
S_9	0	5	0	2

8.2.3 Calculation of relative photoionization cross-sections of the FLU*

In order to calculate the relative photoionization cross-sections of the excited FLU molecules with respect to the ground state FLU, the following assumptions were applied:

- cross-sections for excited states S_1 – S_9 were taken at the equilibrium structure of FLU in the ground state and remained unchanged throughout the TSH-MD simulation;
- Kohn-Sham orbitals (KSO) were treated as Dyson orbitals (i.e. Koopmans' theorem approximation was assumed).

The KSO were taken from PBE/def2-SV(P) single point calculations of FLU in the ground state equilibrium geometry in the form of Gaussian cube files with size $20 \times 20 \times 20$. The increments along different directions corresponded to maximal electron kinetic energies $E_{\text{max}} = \frac{h^2}{2m_e \Delta\alpha^2}$ of 200 ($\alpha = a$), 350 ($\alpha = b$), and 640 ($\alpha = c$) eV. The cubes were padded with 60 additional points from every side to increase the momentum resolution in the fast Fourier transformation (FFT). The transition matrix elements from orbital $|\varphi\rangle$ to continuum motion described by the momentum $\mathbf{p}$ in the dipole approximation:

$$\langle \mathbf{p}| \overbrace{\boldsymbol{\mu}}^{q_e \mathbf{r}} |\varphi\rangle \propto \iiint\limits_{-\infty}^{+\infty} \exp(-i\mathbf{p}\mathbf{r}/\hbar)\mathbf{r}\varphi(\mathbf{r})d\mathbf{r}$$

were calculated by 3D FFT of the padded orbital cube values multiplied by x, y, and z. The absolute cross-section of the photoelectron from orbital $|\varphi\rangle$ in the dipole approximation with momentum $\mathbf{p}$ in the molecular frame ionized by the laser field with polarization $\mathbf{E}_0$ is thus given by the expression:

$$\sigma^{\mathbf{E}_0}_{\varphi \to \mathbf{p}} \propto |\mathbf{E}_0 \cdot \langle \mathbf{p}|\boldsymbol{\mu}|\varphi\rangle|^2 \ .$$

If a randomly oriented ensemble is averaged to account for different directions of $\mathbf{E}_0$ in the molecular frame, this gives the expression:

$$\sigma_{\varphi \to \mathbf{p}} \propto E_0^2 |\langle \mathbf{p}|\boldsymbol{\mu}|\varphi\rangle|^2$$

Since the direction of the photoelectron is not observed in the molecular frame, our observable is an orientation averaged yield of photoelectron with the absolute momentum p:

$$\sigma_{\varphi \to p}(p) \propto \iiint\limits_{-\infty}^{+\infty} \sigma_{\varphi \to \mathbf{p}'} \delta(p - p') d\mathbf{p}' \ . \quad (2)$$

These dependencies for a set of KSO considered are given in Fig. 23.

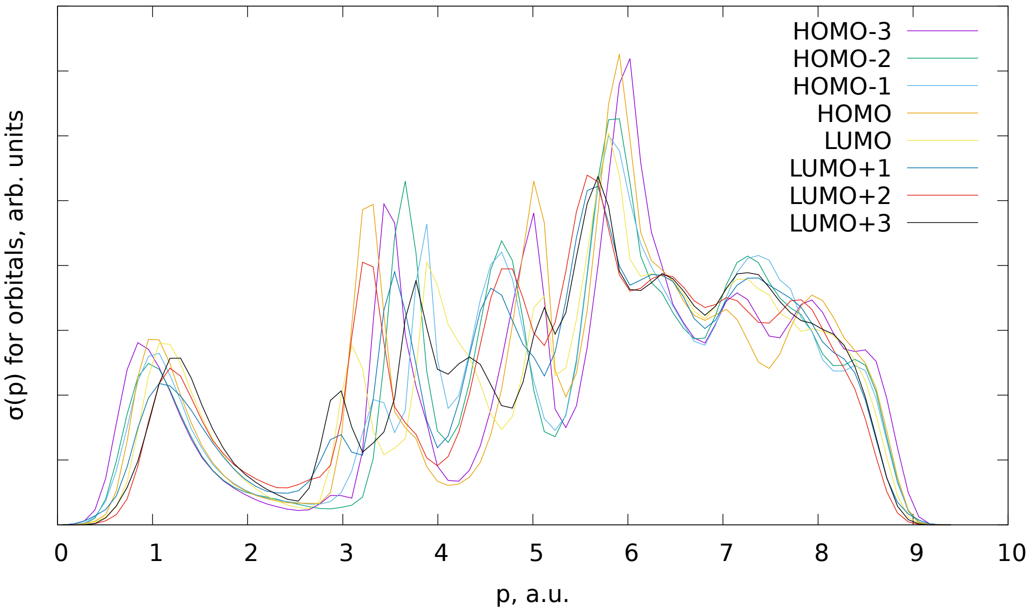

Supplementary Figure 23: Relative cross-sections of the photoelectron yields (Eq. 2) from different Kohn-Sham orbitals at the PBE/def2-SV(P) level of theory.

The photoelectron yields from different orbitals were computed as:

$$\sigma_\varphi \propto \int_0^{+\infty} \exp\left(-\frac{(h\nu - \frac{p^2}{2m_\mathrm{e}} + \varepsilon)^2}{W^2}\right) \sigma(h\nu) dh\nu \ , \tag{3}$$

where $h\nu$ is the photon energy, W is the spectral width of the XUV pulse (0.02 eV), ε is the orbital energy, which is related to vertical ionization potential (vIP) by Koopman's theorem as $\text{vIP} = -\varepsilon$, and $\sigma(h\nu) \propto \sigma_{\varphi\to p}(p = \sqrt{2m_\mathrm{e}(\varepsilon + h\nu)}) \cdot p$, where multiplication by p comes from the Jacobian $dh\nu \propto pdp$. Within the narrow spectral width range, the $\sigma(h\nu)$ for all the KSO were approximately constant, therefore the expression above was replaced by $\sigma_\varphi \approx \sigma(h\nu = 41 \text{ eV})$. The resulting cross-sections, KSO shapes and orbital energies ε can be found in Tables S33 and S34.

The excited states in the TD-DFT were assumed to be expressed as:

$$|S_n\rangle = \sum_{i\in\text{occ}} \sum_{j\in\text{vac}} c_i^j \hat{A}_i^j |S_0\rangle \ ,$$

Supplementary Table S33: Occupied Kohn-Sham orbitals of FLU and the Koopmans' vertical ionization potential/electron affinities (vIP$=-\varepsilon$ with ε being the orbital energy) at the PBE/def2-SV(P) level of theory, and their relative ionization cross-sections σ by a 41 eV photon (normalized to the value of the HOMO).

Orbital	View	vIP [eV]	σ [arb. units]
HOMO − 3	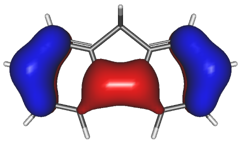	7.0	0.87
HOMO − 2	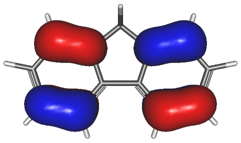	6.2	0.88
HOMO − 1	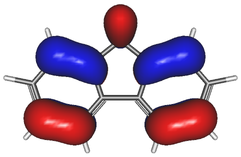	6.0	0.97
HOMO	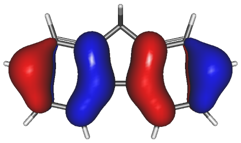	5.4	1.00

where $|S_0\rangle$ is the S_0 Slater determinant, c_i^j are the configuration interaction (CI) coefficients, $\hat{A}_i^j$ are the excitation operators replacing the i-th occupied orbital with j-th vacant. In the scope of this electronic wavefunction, the increase in the photoion yield of the state S_n ($n = 1, 2, \ldots$) with respect to S_0 is approximated as:

$$\sigma(S_n) - \sigma(S_0) \approx \sum_{i \in \text{occ}} \sum_{j \in \text{vac}} |c_i^j|^2 (\sigma_j - \sigma_i) \ , \tag{4}$$

where σ_i/σ_j are the photoionization cross-sections for the KSO given by Eq. 3. The resulting values for states $S_0 - S_9$ are available in Fig. 24.

Supplementary Table S34: Virtual Kohn-Sham orbitals of FLU and their Koopmans' vertical electron affinities (vEA=+ε with ε being the orbital energy) at the PBE/def2-SV(P) level of theory, and their relative ionization cross-sections σ at 41 eV photon energy (normed at HOMO value).

Orbital	View	vIP [eV]	σ [arb. units]
LUMO	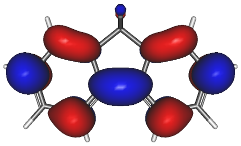	-1.7	1.20
LUMO + 1	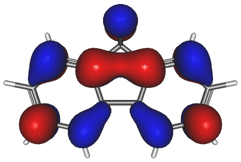	-1.3	1.17
LUMO + 2	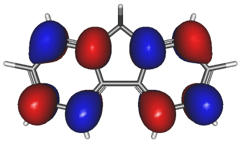	-0.8	1.22
LUMO + 3	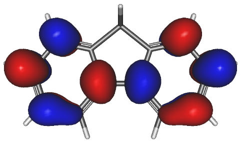	-0.002	1.32

8.2.4 Decay dynamics of the FLU*

Simulations of the electronic excited states relaxation populated by the NIR pulse were performed using TSH-MD methods combined with PBE/def2-SV(P) calculations including ten excited singlet states and one excited triplet state. In total, 100 trajectories were propagated, each of 400 fs in duration with 0.5 fs time step and 25 intermediate steps of electronic wavefunction propagation. The singlet states $S_1 - S_9$ were populated according to Table S32. The resulting populations of the FLU* ensemble produced by the NIR pulse can be seen in the Fig. 25. As one can see, the population from the highly excited states ($S_4 - S_9$) is fully transferred within a short time scale to long-lived states ($S_1 - S_3$) with no further relaxation

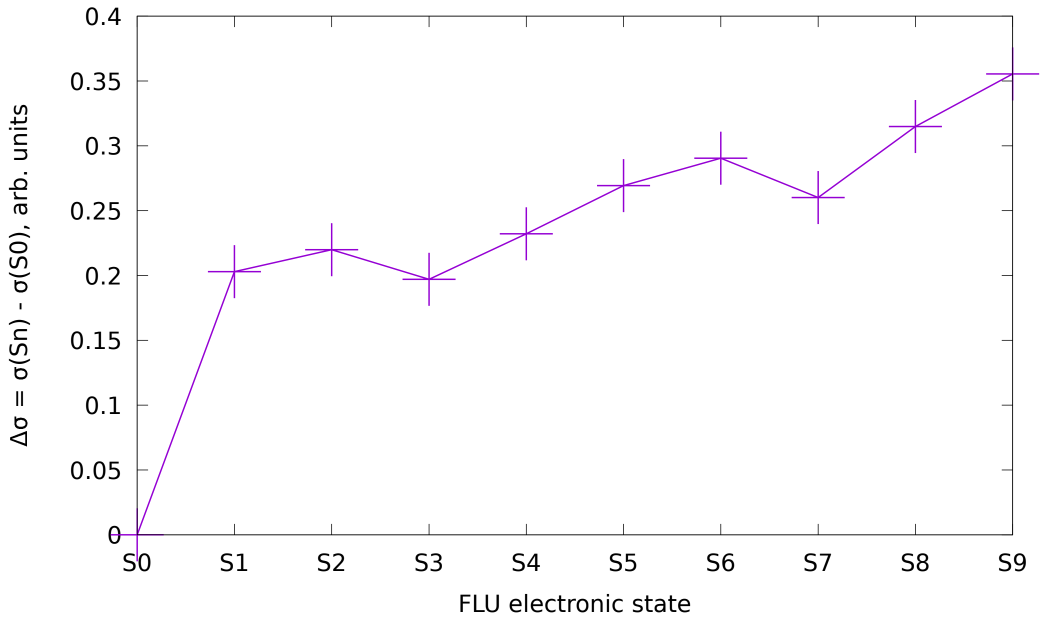

Supplementary Figure 24: Photoionization cross-section increase for states S_0 – S_9 of FLU at the PBE/def2-SV(P) level of theory computed from Eq. 4. See text for details.

to S_0.

In order to produce the time-dependent photoionization yield increase arising from FLU*, we have multiplied each population by the increase in the photoionization cross-section from Fig. 24 and averaged the results for each time frame. The resulting dependence, given in Fig. 26, was fitted by an exponential decay model $\langle \Delta\sigma \rangle(t) \approx a + b \cdot \exp(-t/\tau_\mathrm{d})$, where τ_d is the expected FLU* decay. The resulting value, $\tau_\mathrm{d} = 54 \pm 2$ fs, is in reasonable agreement with our experimentally observed value for FLU (35 ± 8 fs).

8.3 Simulation of XUV pump – NIR probe channel with FLU$^{+,*}$ as an intermediate

8.3.1 Model background

The second channel simulated was the following reaction scheme:

$$\mathrm{FLU} + \mathrm{XUV\ photon} \rightarrow \mathrm{FLU}^{+,*} + \mathrm{e}^-$$

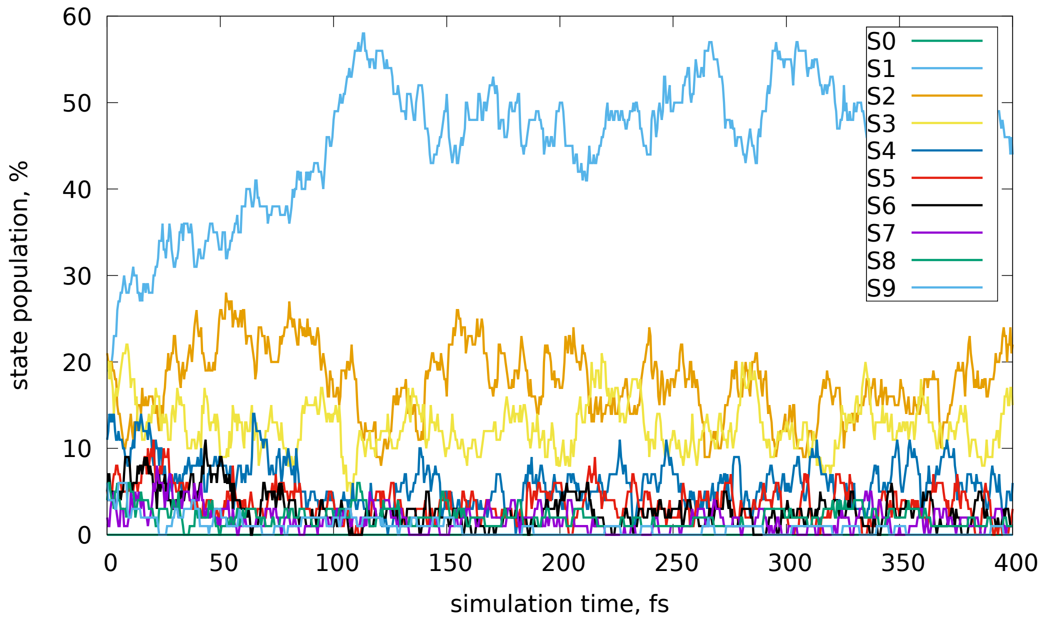

Supplementary Figure 25: Populations of the FLU* ensemble after excitation by the 810 nm laser pulse obtained from TSH-MD simulations. See text for details.

$$\mathrm{FLU}^{+,*} \rightarrow \mathrm{FLU}^{+}_{\mathrm{hot}}$$

$$\mathrm{FLU}^{+,*} + \mathrm{NIR\ photon} \rightarrow \mathrm{FLU}^{2+} + \mathrm{LKE\ e}^{-}$$

The first reaction is the single photon ionization of a neutral FLU molecule by interaction with a high energy XUV photon, leading to the formation of an electronically excited monocation and a high kinetic energy electron. The excited monocation $\mathrm{FLU}^{+,*}$ can convert electronic excitation into the vibrational excitation (second reaction), or interact with a single NIR photon leading to further ionization and production of low kinetic energy (LKE) electrons (with energy $\leq h\nu_{\mathrm{NIR}} = 1.5$ eV). In order to simulate this process, the excited states of the FLU monocation up to the second ionization potential (IP_2) must be considered. In this case, the observable can be extracted from the number of monocations with IEE_1 higher than a threshold ($\mathrm{IP}_2 - h\nu_{\mathrm{NIR}}$). This is shown schematically in Fig. 27.

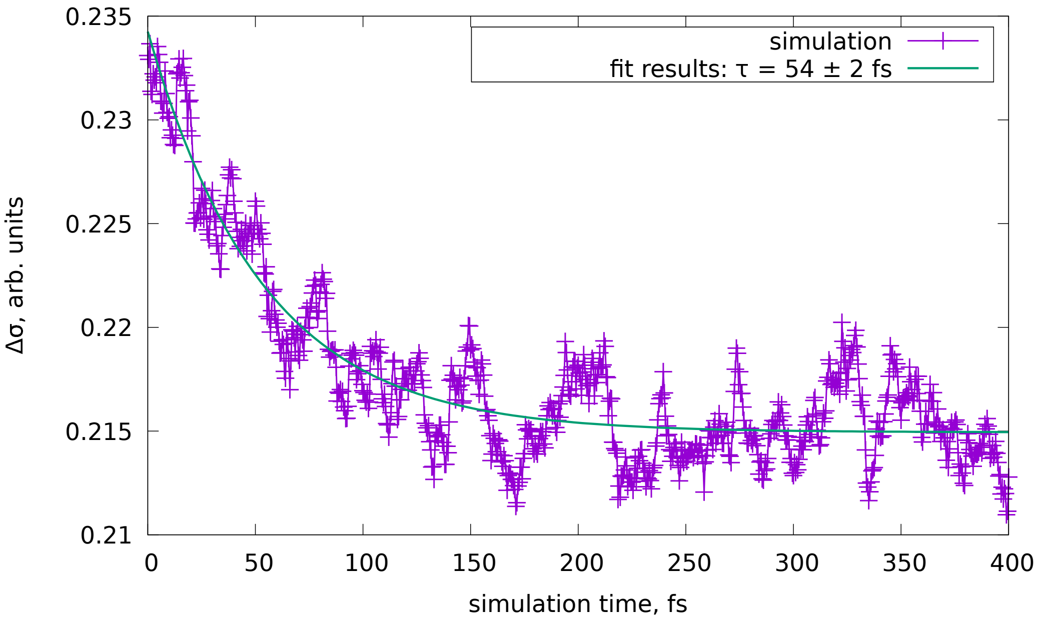

Supplementary Figure 26: The relative increase of the XUV photoionization yield for the FLU* ensemble following excitation with a 810 nm laser pulse obtained from TSH-MD simulations. The extracted lifetime of FLU* is 54 ± 2 fs. See text for details.

8.3.2 Decay dynamics of the $\mathrm{FLU}^{+,*}$

The distribution of $\mathrm{FLU}^{+,*}$ states produced by a single XUV photon can be approximated using the method described by Tikhonov et al.[25]. The population of the excited states of $\mathrm{FLU}^{+,*}$ can be assumed to be given by the following equation:

$$n(\mathrm{IEE}_1) \propto (h\nu_{\mathrm{XUV}} - \mathrm{IP}_1 - \mathrm{IEE}_1)^{1/2} \ ,$$

where n denotes the fraction of molecules in the excited state with energy IEE_1 (internal excess energy, i.e., the energy difference between the ground electronic state of the FLU^+ and current excited state, see Fig. 27), IP_1 is the first vertical ionization potential of neutral FLU, and $h\nu_{\mathrm{XUV}}$ is the XUV photon energy. Since $h\nu_{\mathrm{XUV}} = 41$ eV, $\mathrm{IP}_1 \approx 7.6$ eV, and we are interested in a IEE_1 range of approximately 1.5 eV, we can assume the distribution of the $\mathrm{FLU}^{+,*}$ excited states in the range of interest to be approximately uniform. Single point energy TD-DFT calculations at the FLU equilibrium geometry demonstrate 480 accessible

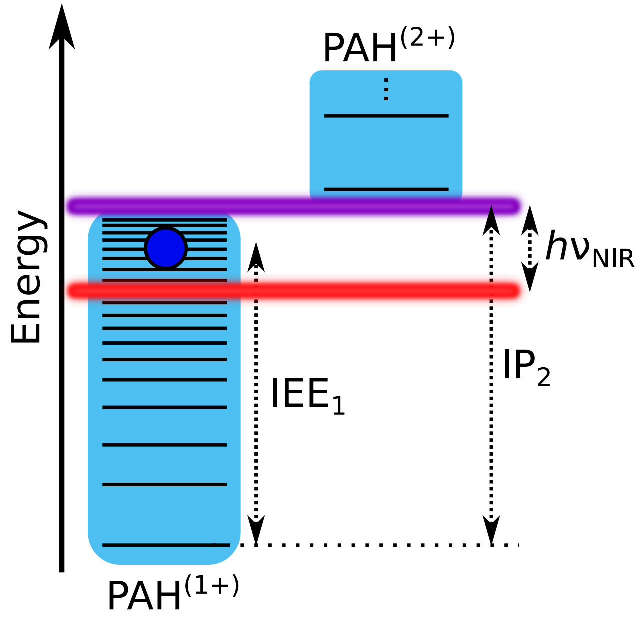

Supplementary Figure 27: Scheme showing the highly electronically excited PAH monocation formed from the XUV pulse and probed by the NIR pulse to produce the PAH dication. The vertical purple line represents the PAH^{2+} ionization threshold and the vertical red line represents the lowest energy from which PAH^{+} can be ionized to PAH^{2+} by a 810 nm photon.

electronic states, of which 180 states fulfill the condition $\mathrm{IP}_2 - h\nu_{\mathrm{NIR}} \leq \mathrm{IEE}_1 \leq \mathrm{IP}_2$, where IP_2 is the second ionization potential (see Fig. 27). Dynamics of so many states cannot be calculated on-the-fly within a reasonable amount of time, therefore an LVC model was constructed for these states. Such electronic excitations most probably have a very complicated potential energy surface topology, therefore TSH-MD simulations are performed for 50 fs to stay within the trusted region of the LVC model.

For each of the 180 initial states, five simulations were performed, giving approximately 900 trajectories in total. Initial conditions for each trajectory were randomly chosen from 100 possible starting states. TSH-MD simulations were driven for 50 fs in total with 0.5 fs time step and 25 intermediate steps of electronic wavefunction propagation. The IEE was computed as a difference between the current electronic energy and the average potential energy from the 100 ground state 100 fs MD trajectories. Initial conditions for the ground state MD of FLU^{+} were sampled from the Wigner distribution corresponding to the ground vibrational state of this particle. The resulting IEE for the trajectory ensemble is given

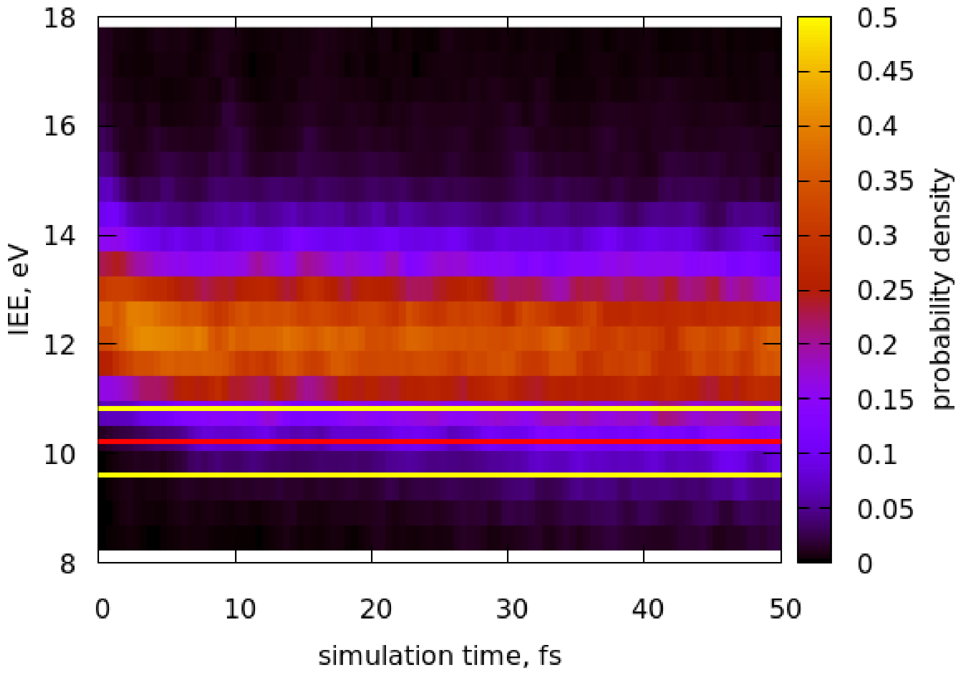

Supplementary Figure 28: $\langle \text{IEE} \rangle (t)$ dependence of $\text{FLU}^{+,*}$ for the 180 states that participate in $\text{FLU}^{+,*} + \text{NIR photon} \rightarrow \text{FLU}^{2+} + \text{LKE e}^-$ channel. The horizontal red line shows a $\text{IP}_2 - h\nu_{\text{NIR}}$ threshold. See text for details.

in Fig. 28, where decay of the electronic excitation on the scale of the simulations can be observed. By considering the number of states able to produce the FLU^{2+} after interaction with a 810 nm photon (i.e. with $\text{IEE}_1 \geq \text{IP}_2 - h\nu_{\text{NIR}}$), the decay shown in Fig. 29 can be extracted. This decay can be fitted to an exponential function $a + b \cdot \exp(-t/\tau_{\text{d}})$, producing an observable decay of $\tau_{\text{d}} = 42 \pm 8$ fs, consistent with our experimental value of 57 ± 13 fs.

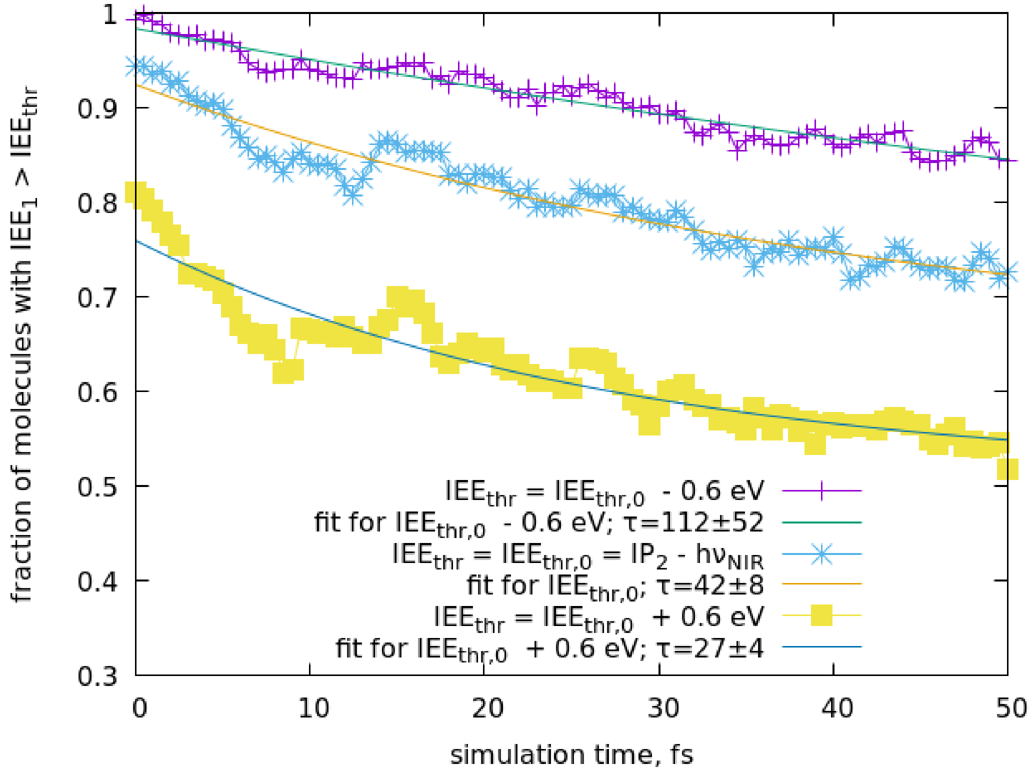

Supplementary Figure 29: Fraction of $FLU^{+,*}$ states that are able to produce the dication via $FLU^{+,*}$ + NIR photon $\rightarrow$ FLU^{2+} + LKE e^- channel. Fitted decay rate constant is 42 ± 8 fs. See text for details.

Supplementary Note 9: Basic properties of the investigated PAHs

Supplementary Table S35: Molecular structures and ionization for the three investigated PAHs. All values are given in eV.

	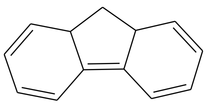 fluorene (FLU) $C_{13}H_{10}$	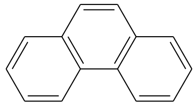 phenanthrene (PHE) $C_{14}H_{10}$	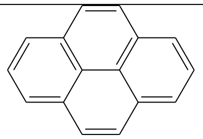 pyrene (PYR) $C_{15}H_{10}$
1st IP[a]	7.88 ± 0.05	7.903	7.426 ± 0.010
2nd IP[b]	13.1	12.7[c]	12.4
3rd IP[d]	17.4	17.2	16.5

[a] – Experimental values from Ref. 26.
[b] – Experimental values from photon impact measurements given in Ref. 27.
[c] – Value for fully deuterated isotopologue ($C_{14}D_{10}$).
[d] – Theoretical values computed at ωB97/def2-TZVPP level of theory.

Supplementary References

(1) Chai, J.-D.; Head-Gordon, M. Systematic optimization of long-range corrected hybrid density functionals. *The Journal of Chemical Physics* **2008**, *128*, 084106.

(2) Zhao, Y.; Truhlar, D. G. The M06 suite of density functionals for main group thermochemistry, thermochemical kinetics, noncovalent interactions, excited states, and transition elements: two new functionals and systematic testing of four M06-class functionals and 12 other functionals. *Theoretical Chemistry Accounts* **2008**, *120*, 215–241.

(3) Rayne, S.; Forest, K. Benchmarking semiempirical, Hartree–Fock, DFT, and MP2 methods against the ionization energies and electron affinities of short- through long-chain [n]acenes and [n]phenacenes. *Canadian Journal of Chemistry* **2016**, *94*, 251–258.

(4) Zhou, B.; Hu, Z.; Jiang, Y.; He, X.; Sun, Z.; Sun, H. Benchmark study of ionization potentials and electron affinities of armchair single-walled carbon nanotubes using density functional theory. *Journal of Physics: Condensed Matter* **2018**, *30*, 215501.

(5) Neese, F. The ORCA program system. *Wiley Interdisciplinary Reviews: Computational Molecular Science* *2*, 73–78.

(6) Neese, F.; Wennmohs, F.; Hansen, A.; Becker, U. Efficient, approximate and parallel Hartree–Fock and hybrid DFT calculations. A 'chain-of-spheres' algorithm for the Hartree–Fock exchange. *Chemical Physics* **2009**, *356*, 98–109, Moving Frontiers in Quantum Chemistry:.

(7) Izsák, R.; Neese, F. An overlap fitted chain of spheres exchange method. *The Journal of Chemical Physics* **2011**, *135*, 144105.

(8) Weigend, F. Accurate Coulomb–fitting basis sets for H to Rn. *Phys. Chem. Chem. Phys.* **2006**, *8*, 1057–1065.

(9) Frasinski, L. J. Covariance mapping techniques. *Journal of Physics B: Atomic, Molecular and Optical Physics* **2016**, *49*, 152004.

(10) Vrakking, M. J. J. An iterative procedure for the inversion of two-dimensional ion/photoelectron imaging experiments. *Review of Scientific Instruments* **2001**, *72*, 4084–4089.

(11) Pedersen, S.; Zewail, A. Femtosecond real time probing of reactions XXII Kinetic description of probe absorption fluorescence depletion and mass spectrometry. *Molecular Physics* **1996**, *89*, 1455–1502.

(12) Shchatsinin, I.; Laarmann, T.; Zhavoronkov, N.; Schulz, C. P.; Hertel, I. V. Ultrafast energy redistribution in C60 fullerenes: A real time study by two-color femtosecond spectroscopy. *The Journal of Chemical Physics* **2008**, *129*, 204308.

(13) Metropolis, N.; Rosenbluth, A. W.; Rosenbluth, M. N.; Teller, A. H.; Teller, E. Equation of State Calculations by Fast Computing Machines. *The Journal of Chemical Physics* **1953**, *21*, 1087–1092.

(14) Press, W. H.; Teukolsky, S. A.; Vetterling, W. T.; Flannery, B. P. *Numerical Recipes*

3rd Edition: The Art of Scientific Computing, 3rd ed.; Cambridge University Press: New York, NY, USA, 2007.

(15) Vishnevskiy, Y. V.; Schwabedissen, J.; Rykov, A. N.; Kuznetsov, V. V.; Makhova, N. N. Conformational and Bonding Properties of 3,3-Dimethyl- and 6,6-Dimethyl-1,5-diazabicyclo[3.1.0]hexane: A Case Study Employing the Monte Carlo Method in Gas Electron Diffraction. *The Journal of Physical Chemistry A* **2015**, *119*, 10871–10881, PMID: 26461037.

(16) Fokin, A. A.; Zhuk, T. S.; Blomeyer, S.; Pérez, C.; Chernish, L. V.; Pashenko, A. E.; Antony, J.; Vishnevskiy, Y. V.; Berger, R. J. F.; Grimme, S.; Logemann, C.; Schnell, M.; Mitzel, N. W.; Schreiner, P. R. Intramolecular London Dispersion Interaction Effects on Gas-Phase and Solid-State Structures of Diamondoid Dimers. *Journal of the American Chemical Society* **2017**, *139*, 16696–16707, PMID: 29037036.

(17) Tully, J. C.; Preston, R. K. Trajectory Surface Hopping Approach to Nonadiabatic Molecular Collisions: The Reaction of H+ with D2. *The Journal of Chemical Physics* **1971**, *55*, 562–572.

(18) Mai, S.; Richter, M.; Heindl, M.; Menger, M. F. S. J.; Atkins, A.; Ruckenbauer, M.; Plasser, F.; Ibele, L. M.; Kropf, S.; Oppel, M.; Marquetand, P.; González, L. SHARC2.1: Surface Hopping Including Arbitrary Couplings — Program Package for Non-Adiabatic Dynamics. sharc-md.org, 2019.

(19) Richter, M.; Marquetand, P.; González-Vázquez, J.; Sola, I.; González, L. SHARC: ab initio Molecular Dynamics with Surface Hopping in the Adiabatic Representation Including Arbitrary Couplings. *J. Chem. Theory Comput.* **2011**, *7*, 1253–1258.

(20) Mai, S.; Marquetand, P.; González, L. Nonadiabatic Dynamics: The SHARC Approach. *WIREs Comput. Mol. Sci.* **2018**, *8*, e1370.

(21) Perdew, J. P.; Burke, K.; Ernzerhof, M. Generalized Gradient Approximation Made Simple. *Phys. Rev. Lett.* **1996**, *77*, 3865–3868.

(22) Weigend, F.; Ahlrichs, R. Balanced basis sets of split valence, triple zeta valence and quadruple zeta valence quality for H to Rn: Design and assessment of accuracy. *Phys. Chem. Chem. Phys.* **2005**, *7*, 3297–3305.

(23) Köuppel, H.; Domcke, W.; Cederbaum, L. S. *Advances in Chemical Physics*; John Wiley & Sons, Ltd, 1984; pp 59–246.

(24) Plasser, F.; Gómez, S.; Menger, M. F. S. J.; Mai, S.; González, L. Highly efficient surface hopping dynamics using a linear vibronic coupling model. *Phys. Chem. Chem. Phys.* **2019**, *21*, 57–69.

(25) Tikhonov, D.; Datta, A.; Chopra, P.; Steber, A.; Manschwetus, B.; Schnell, M. Approaching black-box calculations of pump-probe fragmentation dynamics of polyatomic molecules. *Zeitschrift für Physikalische Chemie* **2020**,

(26) Dabestani, R.; Ivanov, I. N. A Compilation of Physical, Spectroscopic and Photophysical Properties of Polycyclic Aromatic Hydrocarbons. *Photochemistry and Photobiology* **1999**, *70*, 10–34.

(27) Tobita, S.; Leach, S.; Jochims, H. W.; Rühl, E.; Illenberger, E.; Baumgärtel, H. Single- and double-ionization potentials of polycyclic aromatic hydrocarbons and fullerenes by photon and electron impact. *Canadian Journal of Physics* **1994**, *72*, 1060–1069.
